# vizAPA: visualizing dynamics of alternative polyadenylation from bulk and single-cell data

**DOI:** 10.1093/bioinformatics/btae099

**Published:** 2024-03-14

**Authors:** Xingyu Bi, Wenbin Ye, Xin Cheng, Ning Yang, Xiaohui Wu

**Affiliations:** Pasteurien College, Suzhou Medical College of Soochow University, Soochow University, Suzhou 215000, China; Division of Computational Biomedicine, Department of Biological Chemistry, School of Medicine, University of California, Irvine, CA 92697, United States; Pasteurien College, Suzhou Medical College of Soochow University, Soochow University, Suzhou 215000, China; College of Industrial Design, Pukyong National University, Busan 48513, Korea; Pasteurien College, Suzhou Medical College of Soochow University, Soochow University, Suzhou 215000, China

## Abstract

**Motivation:**

Alternative polyadenylation (APA) is a widespread post-transcriptional regulatory mechanism across all eukaryotes. With the accumulation of genome-wide APA sites, especially those with single-cell resolution, it is imperative to develop easy-to-use visualization tools to guide APA analysis.

**Results:**

We developed an R package called vizAPA for visualizing APA dynamics from bulk and single-cell data. vizAPA implements unified data structures for APA data and genome annotations. vizAPA also enables identification of genes with differential APA usage across biological samples and/or cell types. vizAPA provides four unique modules for extensively visualizing APA dynamics across biological samples and at the single-cell level. vizAPA could serve as a plugin in many routine APA analysis pipelines to augment studies for APA dynamics.

**Availability and implementation:**

https://github.com/BMILAB/vizAPA.

## 1 Introduction

Alternative polyadenylation (APA) is an important post-transcriptional regulatory mechanism across all eukaryotes (Mitschka and Mayr 2022). The advent of high throughput sequencing, including 3′ end sequencing and RNA sequencing (RNA-seq), has revealed high tissue and cell-type specificity of APA regulation. More recently, the successful application of single-cell RNA-seq (scRNA-seq) technologies has provided enormous potential to explore APA dynamics across different cell types with single-cell resolution. Accordingly, a myriad of computational tools have been developed for identifying polyadenylation [poly(A)] sites and analyzing APA dynamics from 3′ seq, bulk RNA-seq, and scRNA-seq data (reviewed in Ye *et al.* 2023). In parallel, a variety of visualization tools have been developed to guide bulk and/or single-cell analysis. However, most tools support only gene-level analysis and tools for transcript-level analysis are scarce. VALERIE (Wen *et al.* 2020) and RNA-Scoop (Stephenson *et al.* 2021) can visualize alternative splicing events at the single-cell level, however, they are not applicable to APA events. Millefy (Ozaki *et al.* 2020) is a tool for displaying single-cell read coverage across genomic regions. PolyAMiner-Bulk (Jonnakuti *et al.* 2023), APA-Scan (Fahmi *et al.* 2022), and scDAPA (Ye *et al.* 2019) are tools for detecting APA sites, which also provide functions to plot read coverage in APA genes. However, these three tools only provide bulk-level read coverage visualization, which cannot meet the demands of the growing APA data with larger sample size and higher resolution. Our group previously developed movAPA (Ye *et al.* 2021) for analyzing and visualizing APA dynamics, whereas it cannot visualize read coverage and is not applicable to large single-cell dataset.

We developed an R package called vizAPA for visualizing APA dynamics from bulk or single-cell data. vizAPA implements unified data structures for APA data and genome annotations. vizAPA also enables identification of genes with differential APA usage. Four unique modules are provided in vizAPA for extensively visualizing APA dynamics across biological samples and at the single-cell level.

## 2 Design and implementation

vizAPA mainly consists of six modules (Supplementary Fig. S1). (1) Data input module imports different types of APA data through a compact data structure *PACdataset*. (2) Genome annotation module builds internally an *annoHub* data structure for adapting to different genome annotation sources from different species. (3) Visualization module *vizTracks* generates a genome-browser-like plot, which utilizes tracks to display different types of information related to APA, including gene models, positions and expression levels or usages of poly(A) sites, read alignments, and single-cell read counts. (4) Visualization module *vizStats* generates various charts, including violin plot, boxplot, bubble plot, dot plot, heatmap, etc, to visualize usages of any given poly(A) site(s) in a gene across different cell groups. (5) Visualization module *vizUMAP* learns two-dimensional embeddings for visualizing clusters of cells with similar APA expression or usage profiles. (6) Visualization module *vizAPAmarkers* identifies genes with differential APA usages (called APA markers) and generates rich plots. More details are described in Supplementary Material.

## 3 Application examples

**Figure 1.**
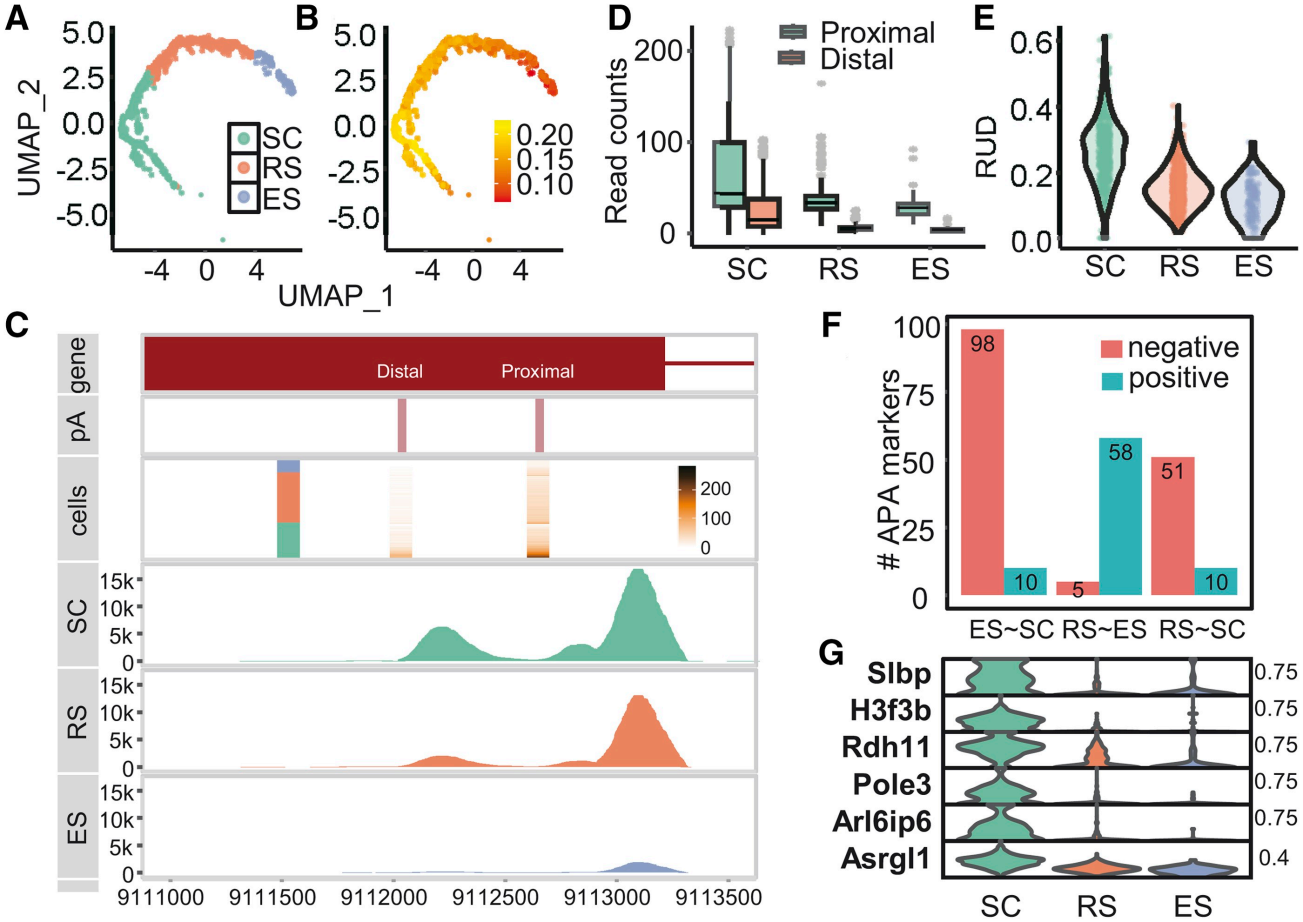
Visualization of APA dynamics during mouse sperm cell differentiation with vizAPA. (A) The low-dimensional representation generated by *vizUMAP* function. (B) Plot of 2D-embeddings generated by *vizUMAP* shows APA usages in single cells. For each cell, the mean RUD score of all APA genes was calculated. (C) A genome-browser-like plot generated by *vizTracks* for the *Asrgl1* gene. (D) Boxplots generated by *vizStats* for *Asrgl1* to show expression levels of individual poly(A) sites. (E) Violin plot summarizing RUD scores of *Asrgl1* in single cells. (F) Numbers of APA markers between every two stages detected with *getAPAmarkers*. Positive markers between group X and Y (X∼Y) are those with significant higher RUD scores in group X. (G) A violin plot generated by *vizAPAMarkers* for six APA markers.

To demonstrate the use of vizAPA, here we adopted a mouse spermatogenesis scRNA-seq dataset (Shulman and Elkon 2019), which sequenced three differentiation stages, including early stage (spermatocytes, SC), intermediate stage (round spermatids, RS), and late stage (elongating spermatids, ES). Poly(A) sites were first identified and quantified by scAPAtrap (Wu *et al.* 2021), which were then loaded and annotated as a *PACdataset* object into vizAPA. The low-dimensional representation of the poly(A) site profile generated by vizAPA’s *vizUMAP* function shows clearly three cell clusters (Fig. 1A). To investigate the global APA dynamics at the single-cell level, we used *vizUMAP* again to overlay the mean APA usage represented by RUD (Relative Usage of Distal poly(A) site) (Wu *et al.* 2021) of each cell on the 2D-embeddings (Fig. 1B). The plot with gradient colors shows gradual transition of 3′ UTR shortening (i.e. decreased RUD scores) during sperm cell differentiation (from SC to RS to ES).

Next, we examined usages of poly(A) sites in a given gene. Here we took the *Asrgl1* gene, an N-terminal nucleophile family member first described as a sperm autoantigen in rats. First, we used *vizTracks* to generate a genome-browser-like plot to display the gene model, locations of poly(A) sites, single-cell expression, and read coverage (Fig. 1C). It can be seen from the “*gene*” and “*pA*” tracks that this gene possesses two poly(A) sites in annotated 3′ UTR. From the read coverage tracks, the proximal site is more dominant than the distal one across all stages. However, the read coverage of both the proximal and distal site is increased from ES to SC, suggesting potential APA dynamics during spermatogenesis. Using *vizStats* to summarize expression levels of individual poly(A) sites, the boxplot also shows the higher expression level of the proximal site than the distal one (Fig. 1D). From the *“cells”* track, heterogeneous poly(A) site expression even in the same cell cluster is observed, which can also be reflected from other plots (Fig. 1B, D, and E). Moreover, profiles of RUD scores in single cells further demonstrate the dynamic usages of poly(A) sites of this gene across the three stages (Fig. 1E).

To further investigate APA dynamics during sperm cell differentiation, we detected genes with differential APA usages, i.e. APA markers between each pair of cell groups with the *getAPAmarkers* function (Fig. 1F). Most markers were identified between SC and ES, with much higher number of negative APA markers (lower RUD scores) in ES than SC. This is consistent with the RUD score distribution presented in the UMAP plot (Fig. 1B). Then *vizAPAMarkers* can be used to visualize selected APA markers with diverse plots, including violin plot, dot plot, heatmap, etc. (Fig. 1G). The violin plot shows that RUD scores of these APA markers in SC are significantly different from other stages. The Supplementary Material includes several user manuals that provide more results of this application example and in-depth use of vizAPA. Supplementary Table S1 compares the functionality of vizAPA with other visualization tools.

## 4 Conclusions

We developed a highly scalable and flexible toolkit, vizAPA, which provides comprehensive functions for visualizing APA dynamics across biological samples and/or at the single-cell level. vizAPA can serve as a plugin in many routine APA analysis pipelines to augment and guide APA analysis, and is valuable for studying APA dynamics and APA-mediated gene regulation from both bulk and single-cell data.

## Supplementary Material

btae099_Supplementary_Data

## Data Availability

The data underlying this article are available in the article and in its online supplementary material.
