# Supplementary material for: vizAPA: visualizing dynamics of alternative polyadenylation from bulk and single-cell data
Source: Bioinformatics. 2024 Mar 14;40(3):btae099. doi: 10.1093/bioinformatics/btae099 (PMC10950478; doi:10.1093/bioinformatics/btae099)
Supplement: btae099_Supplementary_Data [file btae099_supplementary_data.zip › vizAPA_user_mannual_3_Using_vizTracks.pdf]

Using vizTracks in vizAPA: a full tutorial

Xiaohui Wu, Xingyu Bi, Wenbin Ye

2024-01-10

Contents

Overview **2**

Data preparation **2**

Demo PACdataset 2

BAM files 4

Genome annotation **4**

Using a GFF3/GTF file 5

Using OrganismDb 5

Using TxDb 5

Using EnsDb 5

Using BioMart 6

Using customized genome annotation 6

Set priority of annotations 6

Chromosome consistency 6

Chromosome name mapping 8

`vizTracks` to plot gene model, pAs and BAM tracks **8**

Plot tracks in a gene 9

Plot tracks in a specified genomic region 13

Customize pA tracks 14

Customize the cells track 20

Customize BAM tracks 26

Customize the gene model track 28

Plot tracks mannually 30

Session information **34**

Overview

This tutorial takes a `PACdataset` object storing a list of poly(A) sites and BAM files as input and describes full usages of series function related to `vizTracks` in `vizAPA`.

Data preparation

Demo PACdataset

In the package of `vizAPA`, there is a demo `PACdataset` object of mouse sperm cells, containing 974 pAs [poly(A) sites] from 413 genes. There are total 955 cells from three cell types (SC, Spermatocytes; RS, Round spermatids; ES, Elongating spermatids). This PACdataset has been annotated, with both pAs' and cells' meta data.

```
library(vizAPA)

data(scPACds, package='vizAPA')

# summary of the PACdataset
movAPA::summary(scPACds)
```

```
## PAC# 974
## sample# 955
## summary of expression level of each PA
##    Min. 1st Qu.  Median    Mean 3rd Qu.    Max.
##       1      72     957    3151    3636   96363
## summary of expressed sample# of each PA
##    Min. 1st Qu.  Median    Mean 3rd Qu.    Max.
##    1.00   64.25  452.50  452.05  810.00  955.00
## gene# 413
##      nPAC
## 3UTR  974
```

```
# cell meta data
head(scPACds@colData)
```

```
##                  orig.ident nCount_RNA nFeature_RNA RNA_snn_res.0.5
## AAACCTGAGCTTATCG       gene      23617         5061               9
## AAACCTGGTTGAGTTC       gene      19555         4802               9
## AAACCTGTCAACGAAA       gene      23467         5009               8
## AAACGGGCACAGGTTT       gene      28832         5484               8
## AAACGGGTCATTTGGG       gene      18931         4819               8
## AAACGGGTCCTCATTA       gene      15734         3855               8
##                  seurat_clusters celltype       UMAP_1      UMAP_2
## AAACCTGAGCTTATCG               9       RS  0.361751856 4.528803031
## AAACCTGGTTGAGTTC               9       RS -0.119255482 4.563224952
## AAACCTGTCAACGAAA               8       RS  3.023034156 4.074635188
## AAACGGGCACAGGTTT               8       RS  3.322863163  3.81046788
## AAACGGGTCATTTGGG               8       ES   4.73071772 3.419416826
## AAACGGGTCCTCATTA               8       ES  5.306060375 3.274766843
##                           barcode
```

```
## AAACCTGAGCTTATCG AAACCTGAGCTTATCG
## AAACCTGGTTGAGTTC AAACCTGGTTGAGTTC
## AAACCTGTCAACGAAA AAACCTGTCAACGAAA
## AAACGGGCACAGGTTT AAACGGGCACAGGTTT
## AAACGGGTCATTTGGG AAACGGGTCATTTGGG
## AAACGGGTCCTCATTA AAACGGGTCCTCATTA
```

It is better to change the level order of the factor `celltype` in scPACds, which can make the labels in the plots consistent with sperm differentiation from SC to RS to ES.

```
scPACds@colData$celltype=factor(scPACds@colData$celltype,
                                levels=c('SC','RS','ES'))
```

The raw data was obtained from NCBI (accession number: GSM280334), and pAs were extracted by scAPA-trap. The resulted pA list from scAPAtrap was then processed and annotated with MM10 genome annotation (TxDb.Mmusculus.UCSC.mm10.knownGene) in movAPA, and a `PACdataset` object was finally obtained. This object was then used in vizAPA for visualization.

For demonstration Only genes containing at least one top 500 pAs ranked by the total number of reads were retained in the PACdataset.

Generally, it is easy to use `movAPA` to import the result (basically a pA/peak list with per cell counts) from `scAPAtrap` or other tools, following the example code below. Please see the vignettes "Read_PAC_data_from_Sierra" and "Read_PACs_data_from_scAPAtrap" for more details.

```
# First, use readPACds or createPACdataset to create the PACdataset object
# from a pA list, pA-cell counts table, cell meta data
PACds=movAPA::readPACds(pacFile,
                 colDataFile)

movAPA::summary(PACds)

# If there are internal priming artifacts, should be removed first
library(BSgenome.Mmusculus.UCSC.mm10)
bsgenome=BSgenome.Mmusculus.UCSC.mm10
PACds=movAPA::removePACdsIP(PACds, bsgenome)
PACds=PACds$real

# Then annotate the pA list with genome annotation from TxDb or a GFF/GTF file
library(TxDb.Mmusculus.UCSC.mm10.knownGene)
txdb=TxDb.Mmusculus.UCSC.mm10.knownGene

PACds=movAPA::annotatePAC(PACds, aGFF=txdb)

# Then extend 3'UTR by some length, e.g., 1000 bp
# which can recuite potential pAs in downstream intergenic regions of 3'UTR
PACds=movAPA::ext3UTRPACds(PACds, 1000)

# For single cell data, we'd better only use 3'UTR pAs for analysis
# since pAs in other genomic regions may be artifacts
PACds=movAPA::get3UTRAPAds(PACds)

# Finally, a full annotated 3'UTR PACdataset can be obtained
# which could be used in vizAPA for visualization
movAPA::summary(PACds)
```

BAM files

For this tutorial, three very small BAM files corresponding to the three cell types (SC, ES, and RS), which contains only mapped reads from five genes, were made from the original BAM files.

The `readBAMFileNames` function reads the BAM file names into a data frame recording file name, group, and label. Each row in the data frame stores the information of a BAM file. Normally, a BAM file represents a condition, e.g., cell type. The `readBAMFileNames` function will check the existence of each file (and the corresponding .bai file)! The BAM files could be in different folders, please see `?readBAMFileNames` for more examples.

The BAM files for the following analysis can be downloaded here:mouse.sperm.bam

```
#Create the list of BAM files
bam.files=c("dedup_GSM2803334.ES.mini.sorted.bam",
            "dedup_GSM2803334.RS.mini.sorted.bam",
            "dedup_GSM2803334.SC.mini.sorted.bam")
bam.groups=c("ES","RS","SC")
bam.labels=c("ES" ,"RS","SC")
bam.path='./'

# here we set bam.order to make BAM colors consistent with PACdataset
bams<-readBAMFileNames(bam.files=bam.files,
                        bam.path=bam.path,
                        bam.labels = bam.labels,
                        bam.groups = bam.groups,
                        bam.order=c('SC','RS','ES'))
bams
```

```
##                                    fileName group label
## 3 ./dedup_GSM2803334.SC.mini.sorted.bam    SC    SC
## 2 ./dedup_GSM2803334.RS.mini.sorted.bam    RS    RS
## 1 ./dedup_GSM2803334.ES.mini.sorted.bam    ES    ES
```

Genome annotation

In vizAPA, the genome annotation is used for the track plots to show gene models in a genomic region. The genome annotation could be retrieved from several sources, including gff3/gtf file, TxDb, EnsDb, BioMart, and OrganismDb. Users can provide one or more annotation sources. Even if no publicly availabe genome annotation exists, vizAPA can plot simply a gene or a pA region to represent the gene model.

Following are commonly used genome annotation sources:

- OrganismDb object: recommended, support gene symbols and other combination of columns as label.
- TxDb object: don't support gene symbol labeling.
- EnsDb object: supports gene symbol labeling, filtering etc.

Object type	example package/object	name contents
OrgDb	org.Hs.eg.db	gene based info. for Homo sapiens
TxDb	TxDb.Hsapiens.UCSC.hg19.knownGene	transcriptome ranges for Homo sapiens

Object type	example package/object	name contents
OrganismDb	Homo.sapiens	composite information for Homo sapiens
gff3/gtf file	Homo_sapiens.GRCh38.96.gtf	gtf file for Homo sapiens
biomaRt	hsapiens_gene_ensembl	biomaRt for Homo sapiens
genes	a data frame with chr/strand/start/end/gene_xx_ids	customized annotation in vizAPA

We can make an `annoHub` object with elements named txdb/gff/orgdb/ensdb/biomart/genes in its `annos` slot denoting different annotation sources. This `annoHub` object could be used by many functions in vizAPA.

```
annoSource=new("annoHub")
```

Using a GFF3/GTF file

Given a GFF3/GTF file, the `useGff` function can be used to parse the GFF3/GTF file to a data frame, which can be added to the annoHub. Here is the example code. However, it would be more convenient to use other sources of genome annotations, e.g., EnsDb, BioMart, and OrganismDb,

```
gff <- useGff(gff ="gencode.vM32.annotation.gff3")
annoSource=addAnno(annoSource, gff)
```

Using OrganismDb

We recommend to use `OrganismDb` as it contains both gene symbol and entrez id, which is easier to use.

```
library(Mus.musculus, quietly = TRUE)
orgdb=Mus.musculus
annoSource=addAnno(annoSource, orgdb)
annoSource=setDefaultAnno(annoSource, 'orgdb')
```

Using TxDb

The annoHub object in vizAPA allows adding multiple annotations, users can set default annotation for visualization.

```
library(TxDb.Mmusculus.UCSC.mm10.knownGene, quietly = TRUE)
txdb=TxDb.Mmusculus.UCSC.mm10.knownGene
annoSource=addAnno(annoSource, txdb)
```

Using EnsDb

```
library(EnsDb.Mmusculus.v79, quietly = TRUE)
ensdb=EnsDb.Mmusculus.v79
annoSource=addAnno(annoSource, ensdb)
```

Using BioMart

```
library(biomaRt)
bm = biomaRt::useMart("ensembl", dataset = "mmusculus_gene_ensembl")
annoSource=addAnno(annoSource, bm)
```

Using customized genome annotation

The `annoHub` supports user provided data frame with genomic ranges of genes, which is named `genes` in `\@annos`. Here we extracted all genes from `OrganismDb` and add it to `annoSource` for demonstration.

```
genes=getAnnoGenes(orgdb)
annoSource=addAnno(annoSource, genes)
```

Set priority of annotations

When searching given genes for genomic ranges in an `annoHub`, the searching order is the default annotation followed by other annotations in the order of `\@annos`' elements. We can set orders of the priority by reorder the elements in the annos slot.

```
## re-order annoSource to set the searching priority
names(annoSource@annos)
```

```
## [1] "orgdb"   "txdb"    "ensdb"   "biomart" "genes"
```

```
annoSource@annos=annoSource@annos[c('orgdb','txdb','ensdb','genes','biomart')]
annoSource
```

```
## @annos [annotation sources]:
## orgdb=OrganismDb
## txdb=TxDb
## ensdb=EnsDb
## genes=data.frame
## biomart=Mart
## @defaultAnno:
##  orgdb
```

Chromosome consistency

If multiple sources of genome annotation are provided, we can check the consistency of chromosome names among different sources, use the function `isChrConsistent`. Normally, main chromosome names (e.g., chr1, 1) are the same across different annotations, scaffolds may be different, we can set `exact=FALSE` to allow partial overlapping of chromosome names.

```
## The chr names are different: orgdb is chr1, ensdb is 1.
isChrConsistent(annoSource['orgdb'], annoSource['ensdb'], exact=TRUE)
```

```
## chrs not consistent!
## obj1: chr1 chr2 chr3 chr4 chr5 chr6 chr7 chr8 chr9 chr10 ...
## obj2: 1 10 11 12 13 14 15 16 17 18 ...
```

```
## [1] FALSE
```

```
isChrConsistent(annoSource['orgdb'], annoSource['ensdb'], exact=FALSE)
```

```
## chrs not consistent!
## obj1: chr1 chr2 chr3 chr4 chr5 chr6 chr7 chr8 chr9 chr10 ...
## obj2: 1 10 11 12 13 14 15 16 17 18 ...
```

```
## [1] FALSE
```

```
## The chr names are total the same between txdb and orgdb, both are like chr1
isChrConsistent(annoSource['txdb'], annoSource['orgdb'], exact=TRUE)
```

```
## [1] TRUE
```

We can also check the consistency of chromosome names among PACdataset, BAM files, and genome annotations.

```
# chr names are the same in PACds and in BAM file
isChrConsistent(scPACds, bams, exact=TRUE)
```

```
## [1] TRUE
```

```
# Here for annoSource, the default anno was used.
# It seems that some chromosomes do not have exacytly the same name
isChrConsistent(scPACds, bams, annoSource, exact=TRUE)
```

```
## chrs not consistent!
## obj1: chr1 chr10 chr11 chr12 chr13 chr14 chr15 chr16 chr17 chr18 ...
## obj2: chr1 chr2 chr3 chr4 chr5 chr6 chr7 chr8 chr9 chr10 ...
```

```
## [1] FALSE
```

```
# However, the main chr names are the same
isChrConsistent(scPACds, bams, annoSource, exact=FALSE)
```

```
## [1] TRUE
```

```
# We then get full chromosome names of each object,
# and it would be fine that main chromosomes have the same name.
getChrs(scPACds)
```

```
##  [1] "chr17" "chr14" "chr12" "chr4"  "chr19" "chr8"  "chr15" "chr1"  "chr16"
## [10] "chr13" "chr5"  "chr7"  "chr10" "chr11" "chr3"  "chr2"  "chr18" "chr9"
## [19] "chr6"  "chrX"
```

```
getChrs(bams)
```

```
##  [1] "chr1"       "chr10"      "chr11"      "chr12"      "chr13"     
##  [6] "chr14"      "chr15"      "chr16"      "chr17"      "chr18"     
## [11] "chr19"      "chr2"       "chr3"       "chr4"       "chr5"      
## [16] "chr6"       "chr7"       "chr8"       "chr9"       "chrM"      
## [21] "chrX"       "chrY"       "JH584299.1" "GL456233.1" "JH584301.1"
## [26] "GL456211.1" "GL456350.1" "JH584293.1" "GL456221.1" "JH584297.1"
## [31] "JH584296.1" "GL456354.1" "JH584294.1" "JH584298.1" "JH584300.1"
## [36] "GL456219.1" "GL456210.1" "JH584303.1" "JH584302.1" "GL456212.1"
## [41] "JH584304.1" "GL456379.1" "GL456216.1" "GL456393.1" "GL456366.1"
## [46] "GL456367.1" "GL456239.1" "GL456213.1" "GL456383.1" "GL456385.1"
## [51] "GL456360.1" "GL456378.1" "GL456389.1" "GL456372.1" "GL456370.1"
## [56] "GL456381.1" "GL456387.1" "GL456390.1" "GL456394.1" "GL456392.1"
## [61] "GL456382.1" "GL456359.1" "GL456396.1" "GL456368.1" "JH584292.1"
## [66] "JH584295.1"
```

We can also get chr names for different annotations in `annoSource`.

```
getChrs(annoSource, which='orgdb') # chr1
getChrs(annoSource, which='ensdb') # 1
getChrs(annoSource, which='biomart') # 1
getChrs(annoSource, which='genes') # chr1
getChrs(annoSource, which='txdb') # chr1
```

Chromosome name mapping

Since there are different annotations in the `annoSource`, we can add the `chrMappings` slot to coordinate the chromosome names among different annotation sources. Given `chrMappings`, vizAPA can automatically use appropriate chr names in its functions.

```
chrMappings=data.frame(cn1=c(1:19, 'X','Y'))
chrMappings$cn2=paste0('chr', chrMappings$cn1)
annoSource@chrMappings=chrMappings
head(chrMappings)
```

```
##   cn1  cn2
## 1   1 chr1
## 2   2 chr2
## 3   3 chr3
## 4   4 chr4
## 5   5 chr5
## 6   6 chr6
```

vizTracks to plot gene model, pAs and BAM tracks

`vizTracks` gets all tracks for a genomic region, a gene, or PAs, including gene model track, pA track(s), cells track, and BAM track(s).

- **gene model track**: plot gene models given a specific region or a gene symbol/ID, according to the annotation(s) in the `annoHub` object.
- **pA track**: plot the positions, expression levels, APA ratios for a PACdataset or multiple PACdataset objects.

- **cells track**: plot counts or ratios of individual cells using gradient colors.
- **BAM track**: plot BAM coverage using lines or areas for individual cell groups or merged cell groups.

Plot tracks in a gene

Here we plot for a gene all available tracks including gene model, BAM coverages, pA coordinates, and individual cell distributions. The gene model is based on the annotation provided in `annoSource` (here is the default element `annoSource['orgdb']`). The pAs in this gene are represented as a expanded 21 bp (2*`PA.width`+1) region of `coord`. Only the position rather than the expression of pAs is shown (PA.show='pos'). BAM coverage information is from `bams`. The 5' and 3' ranges of the gene model are expanded by 300 bp (`space5` and `space3`).

Gene information

In the following we used gene Asrgl1 (Entrezid=66514) for demonstration, as this gene was present in our demo BAM files and in demo PACdataset.

```
gene=66514

## show this gene in PACds
scPACds@anno[scPACds@anno$gene==gene, c(1:6, 10:12)]
```

```
##           chr strand   coord   start     end  ftr  gene gene_start gene_end
## PA16509 chr19      - 9112654 9112654 9113210 3UTR 66514    9109868  9135636
## PA16510 chr19      - 9112037 9112037 9112602 3UTR 66514    9109868  9135636
```

```
## the gene is represented as Entrez ID in PACds, we can show its symbol name
## using 'genes' table in the annos slot
annoSource@annos$genes[annoSource@annos$genes$gene_entrezid==gene, ]
```

```
##         chr strand   start     end gene_entrezid       gene_ensembl gene_symbol
## 18250 chr19      - 9109868 9135636         66514 ENSMUSG00000024654      Asrgl1
```

Basic `vizTracks` for a gene

Plot an example gene, with gene model, pA coordinates, and BAM coverages. Here we extend the gene model by 1000 bp at both ends.

```
# here gm.reduce=FALSE to show full features of gene model instead of collapsed
vizTracks(gene=gene,
          PACds.list=list(pA=scPACds), PA.show=c("pos"),
          annoSource=annoSource,
          PA.columns="coord", PA.width=10,
          space5=1000, space3=1000,
          vizTheme=list(gm.reduce=FALSE))
```

```
## Plot tracks for region: chr19:-:9108868:9136636
## Get gene model track from annoSource[ orgdb ]...
## Get PACds track...
## chr19:-:9108868:9136636
```

```r
# plot collapsed gene model and the BAM coverage
vizTracks(gene=gene,
          bams=bams, PACds.list=list(pA=scPACds), PA.show=c("pos"),
          annoSource=annoSource,
          PA.columns="coord", PA.width=10,
          space5=1000, space3=1000)
```

```
## Plot tracks for region: chr19:-:9108868:9136636
## Get gene model track from annoSource[ orgdb ]...
## Get PACds track...
## chr19:-:9108868:9136636
## Get BAM tracks...
```

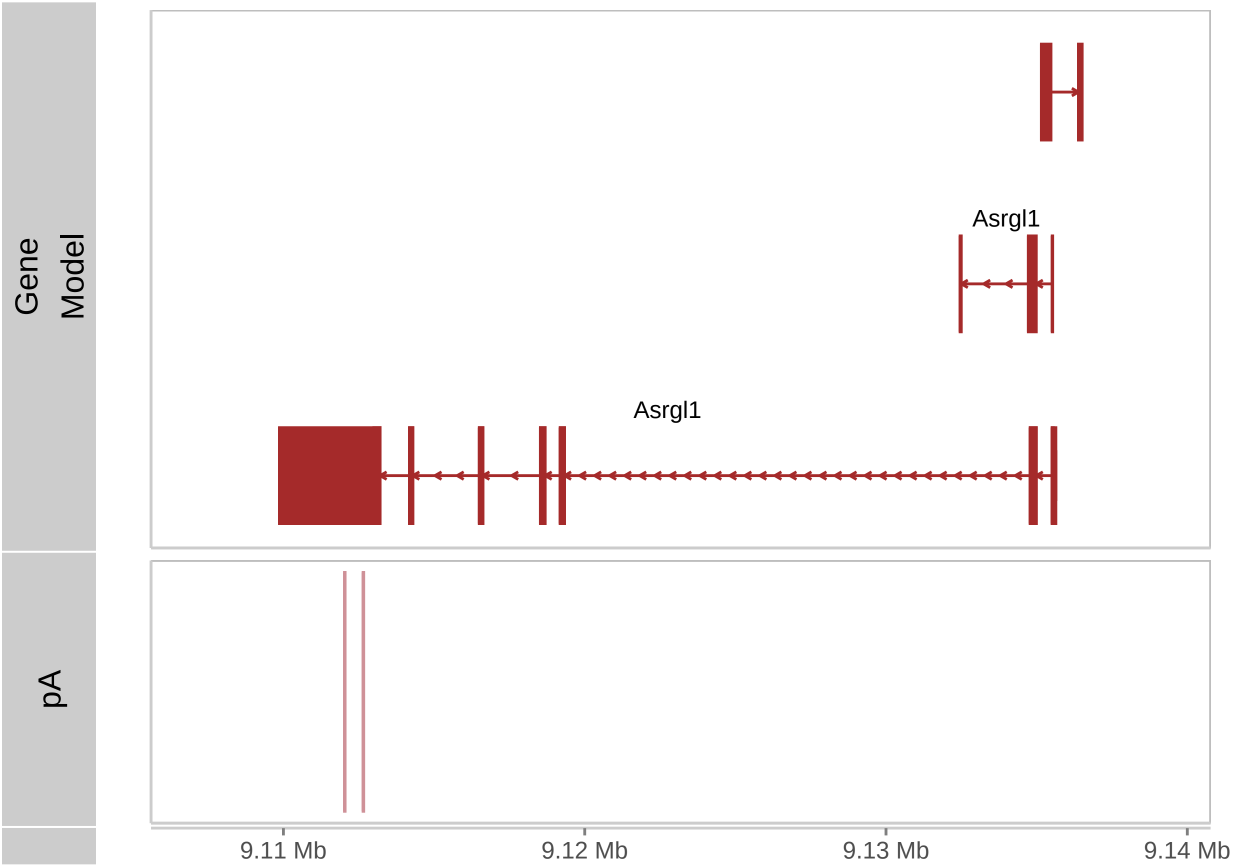

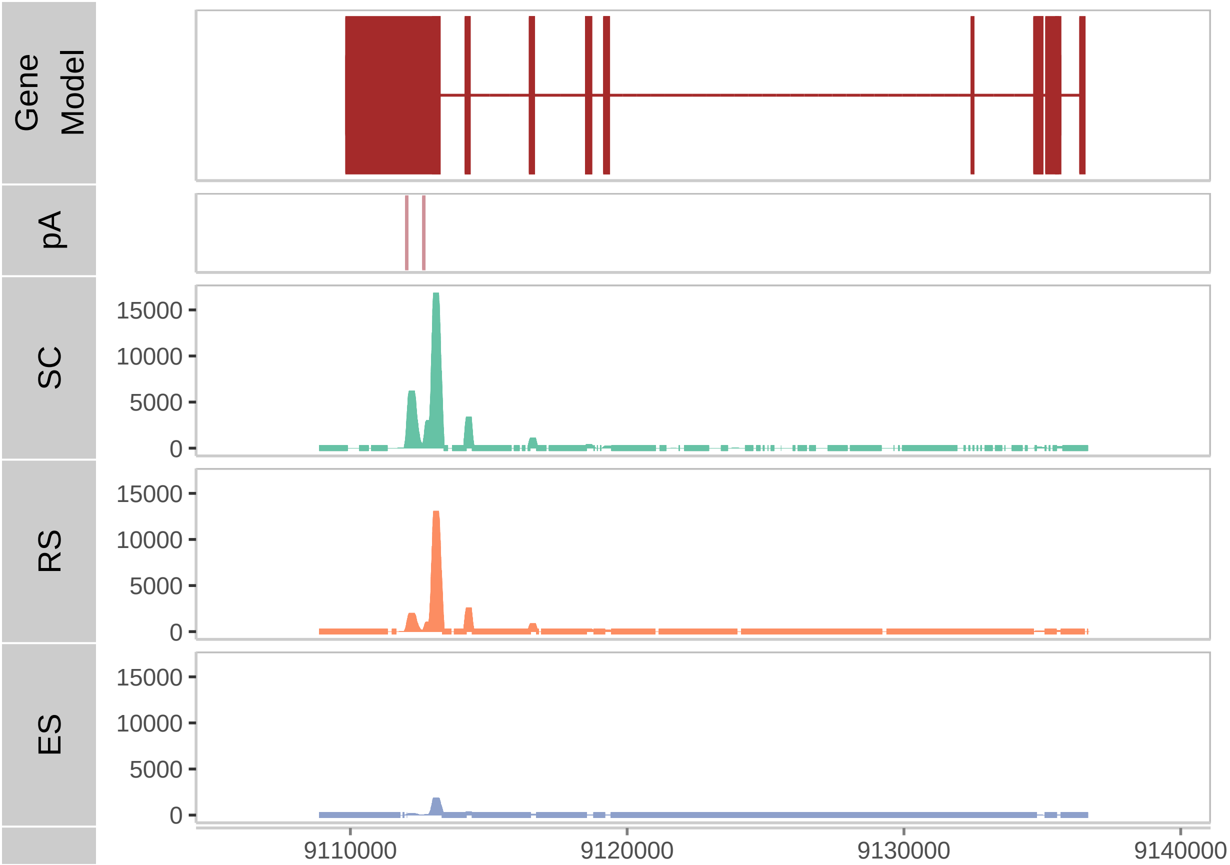

Add heatmap for individual cells

We can also plot the pA expression in individual cells as a heatmap.

```
vizTracks(gene=gene,
          bams=bams, PACds.list=list(pA=scPACds), PA.show=c("pos"),
          cells=TRUE, cells.group='celltype',
          cells.method=c('sum'), cells.sort=c('group'),
          cells.width=100,
          annoSource=annoSource,
          PA.columns="coord", PA.width=10, logPA=TRUE,
          space5=1000, space3=1000)
```

```
## Plot tracks for region: chr19:-:9108868:9136636
## Get gene model track from annoSource[ orgdb ]...
## Get PACds track...
## chr19:-:9108868:9136636
## Get cells track...
## Get BAM tracks...
```

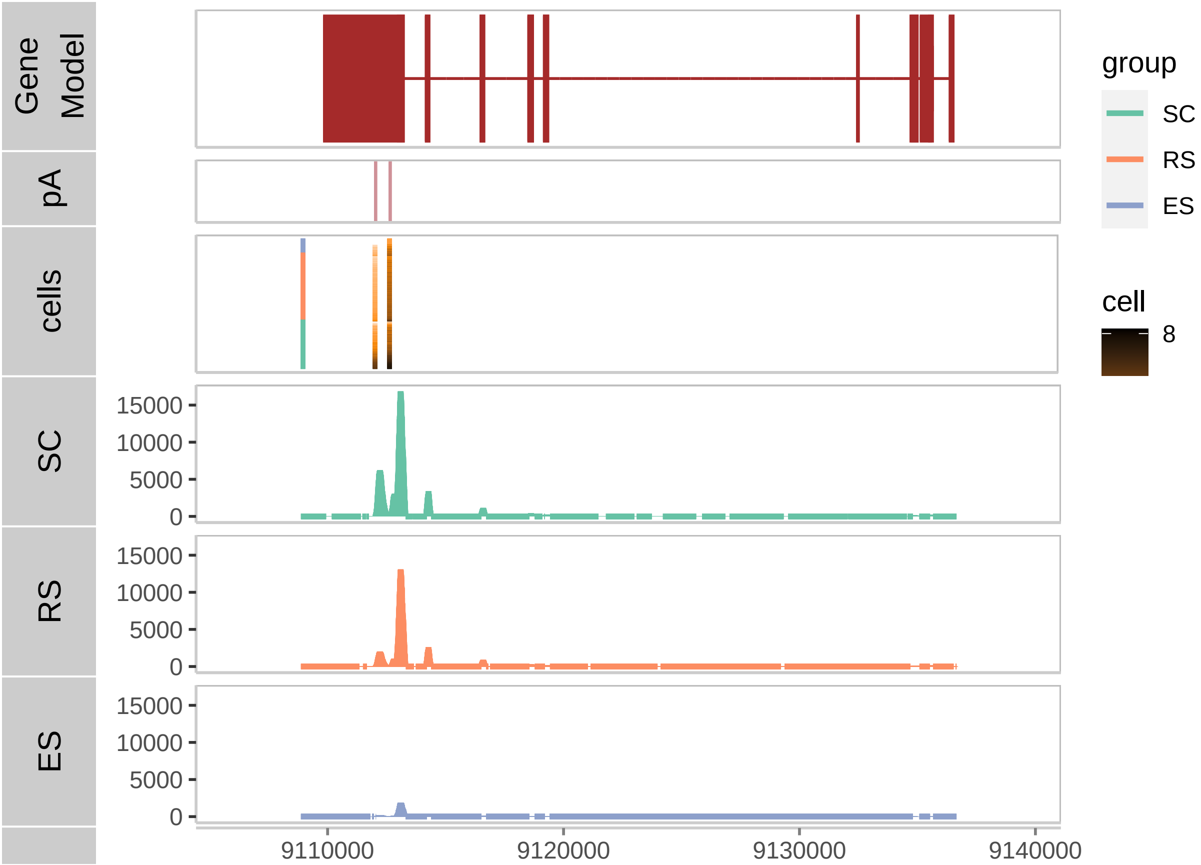

Change colors by `vizTheme`

However, in the above figure, the colors are not consistent between the BAM track and the cells track, as different track are independent. We can set `vizTheme` to make cells' group colors as the order or BAM files. The global variable `vizTHEME` stores all visualization parameters for vizAPA, see `?setVizTheme` for details.

```
# Show default vizTheme parameters
setVizTheme(NULL)
```

```
# set colors of the cells track and BAM coverage
cells.group.cols=c('red','green','blue')
names(cells.group.cols)=c('ES','RS','SC')
vizTheme=list(cells.group.cols=cells.group.cols, bams.col=cells.group.cols)

vizTracks(gene=gene,
          bams=bams, PACds.list=list(pA=scPACds), PA.show=c("pos"),
          cells=TRUE, cells.group='celltype',
          cells.method=c('sum'), cells.sort=c('group'),
          cells.width=100,
          annoSource=annoSource,
          PA.columns="coord", PA.width=10, logPA=TRUE,
          space5=1000, space3=1000,
          vizTheme=vizTheme)
```

```
## Plot tracks for region: chr19:-:9108868:9136636
```

```
## Get gene model track from annoSource[ orgdb ]...
## Get PACds track...
## chr19:-:9108868:9136636
## Get cells track...
## Get BAM tracks...
```

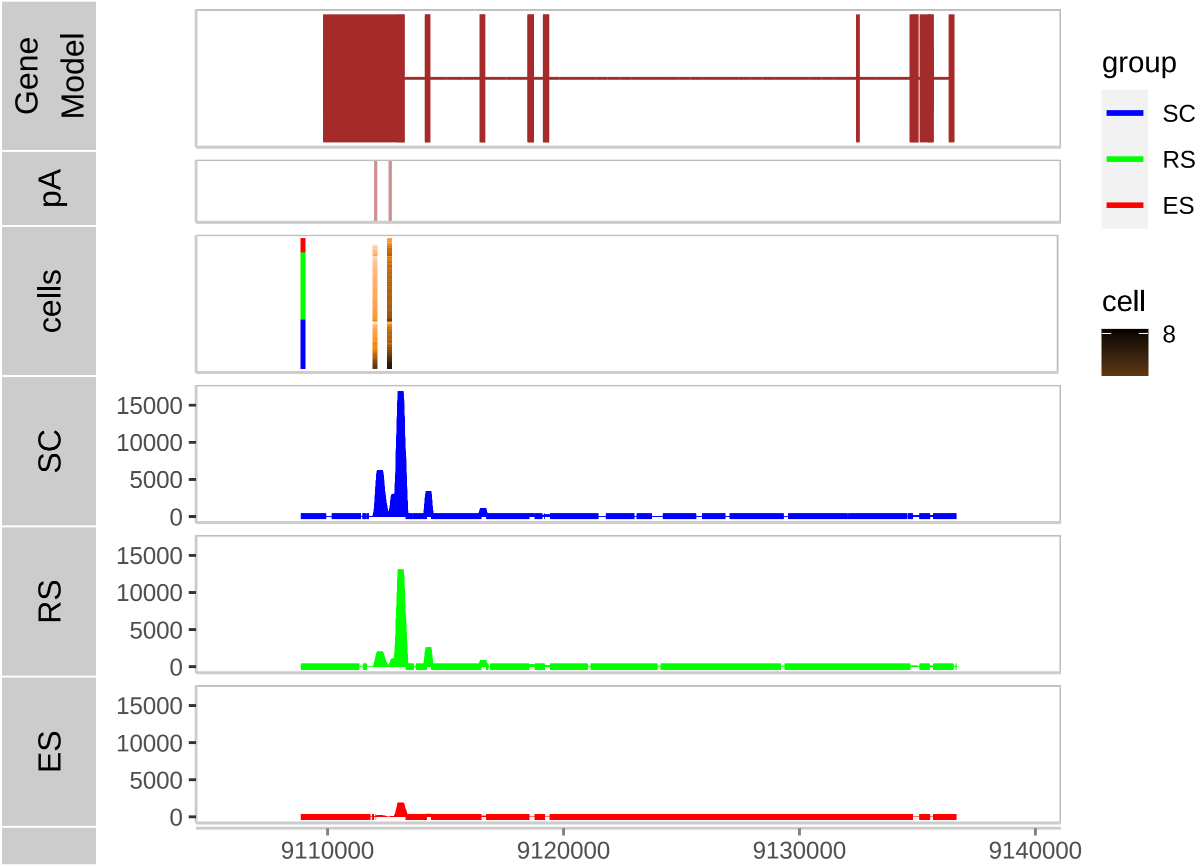

Plot tracks in a specified genomic region

In addition to plot tracks in a given gene, we can also plot tracks in a given genomic region by setting `genomicRegion` instead of `gene` in `vizTracks`.

Get gene's region

```
# here for demonstration, we get the genomic region of the gene
genesGR=getGenesRange(gene, annoSource, rt='str')
genesGR
```

```
## [1] "chr19:-:9109868:9135636"
```

Basic vizTracks for a region

```r
vizTracks(genomicRegion=genesGR,
          bams=bams, PACds.list=list(pA=scPACds), PA.show=c("pos"),
          cells=TRUE, cells.group='celltype',
          cells.method=c('sum'), cells.sort=c('group'),
          cells.width=100,
          annoSource=annoSource,
          PA.columns="coord", PA.width=10, logPA=TRUE,
          space5=1000, space3=1000,
          vizTheme=NULL)
```

```
## Plot tracks for region: chr19:-:9108868:9136636
## Get gene model track from annoSource[ orgdb ]...
## Get PACds track...
## chr19:-:9108868:9136636
## Get cells track...
## Get BAM tracks...
```

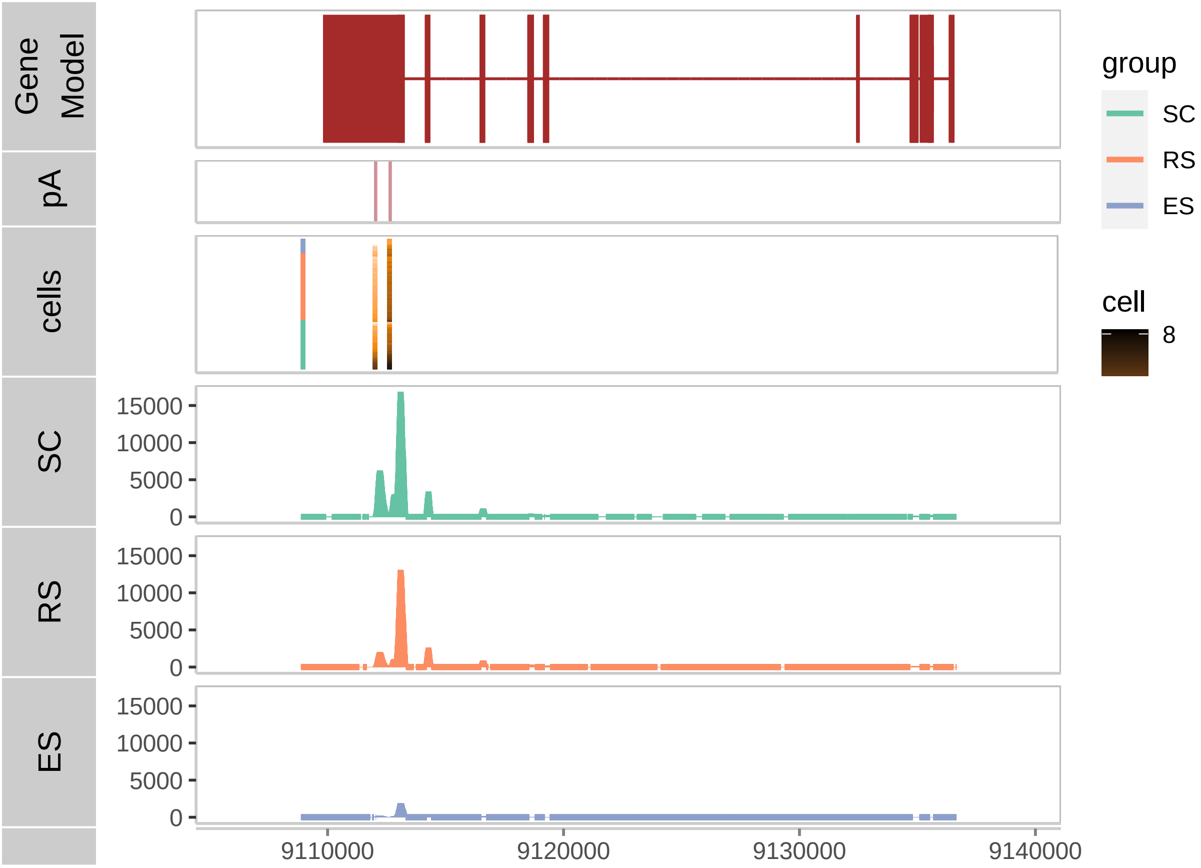

Customize pA tracks

This demo `PACdataset` provides also the range of each pA (the start and end columns in `PACds\@anno`, so we can represent pAs as a range by providing the `PA.columns="start:end"`. In this case, the value of `PA.width` or `cells.width` will be ignored, and the bar width of each pA is the `start-end+1` of the respective pA.

Plot pA regions

```
# show the region of the pA
scPACds@anno[1:5, 1:5]
```

```
##            chr strand    coord    start      end
## PA6062   chr17      +  6607918  6607411  6607918
## PA15501 chr17      -  6648938  6648938  6649363
## PA6073   chr17      + 13376977 13376187 13376977
## PA6074   chr17      + 13377115 13377041 13377115
## PA6072   chr17      + 13377893 13377588 13377893
```

```
# plot pA regions by setting PA.columns
vizTracks(genomicRegion=genesGR,
          bams=bams, PACds.list=list(pA=scPACds), PA.show=c("pos"),
          cells=FALSE,
          annoSource=annoSource,
          PA.columns="start:end", PA.width=10, logPA=TRUE,
          space5=1000, space3=1000,
          vizTheme=NULL)
```

```
## Plot tracks for region: chr19:-:9108868:9136636
## Get gene model track from annoSource[ orgdb ]...
## Get PACds track...
## chr19:-:9108868:9136636
## Get BAM tracks...
```

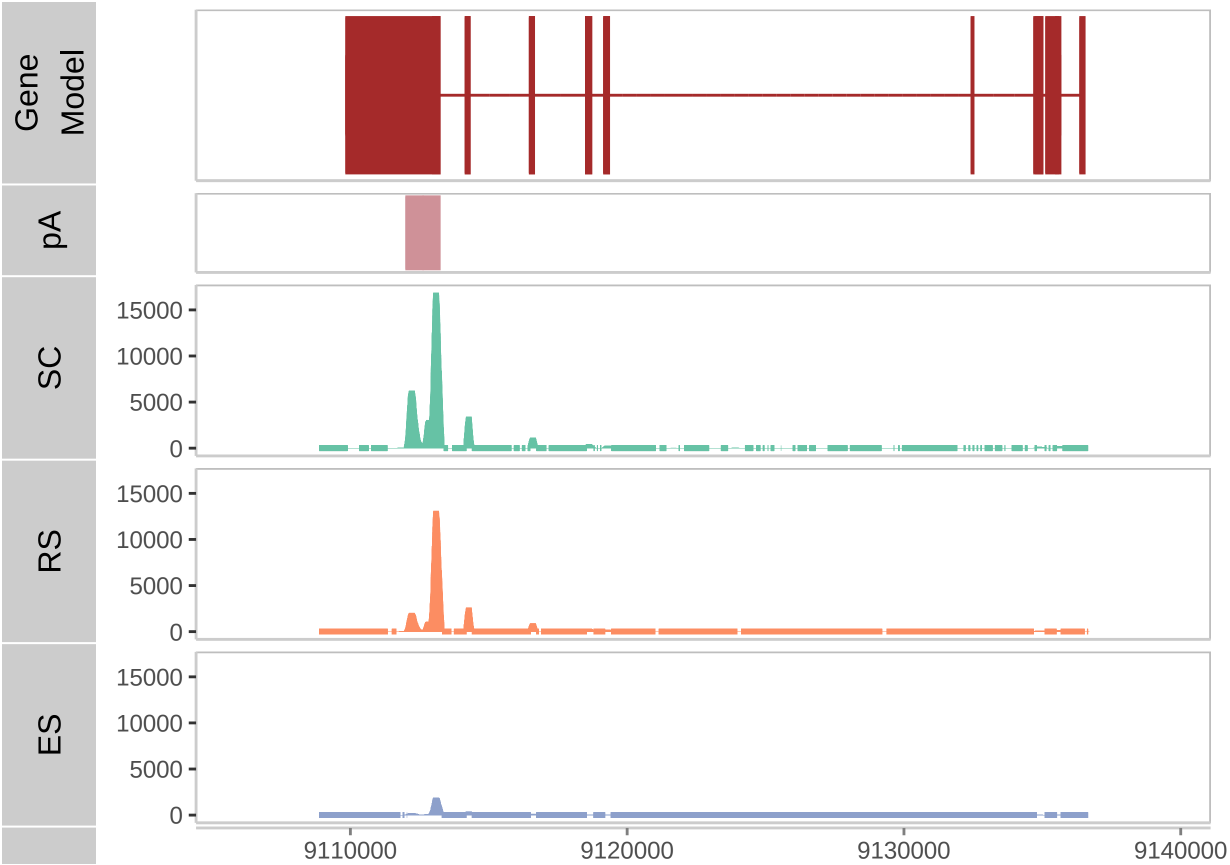

Plot pA values

We can also add additional tracks of expression values (counts of ratios) of pAs, by adding the column(s) of expression value in `PACds\@anno` or `PACds\@counts` to `PA.show`.

```
## Here we add a tot_tagnum to the PACds@anno,
## and show the total expression of pAs
scPACds@anno$tot_tagnum=Matrix::rowSums(scPACds@counts)

## We can do log-transformation to show the tot_tagnum clearly.
scPACds@anno$tot_tagnum=log2(scPACds@anno$tot_tagnum+1)

vizTracks(genomicRegion=genesGR,
          bams=NULL, PACds.list=list(pA=scPACds),
          PA.show=c("pos", "tot_tagnum"),
          annoSource=annoSource,
          PA.columns="coord", PA.width=10, logPA=TRUE,
          space5=1000, space3=1000,
          vizTheme=NULL)
```

```
## Plot tracks for region: chr19:-:9108868:9136636
## Get gene model track from annoSource[ orgdb ]...
## Get PACds track...
## chr19:-:9108868:9136636
```

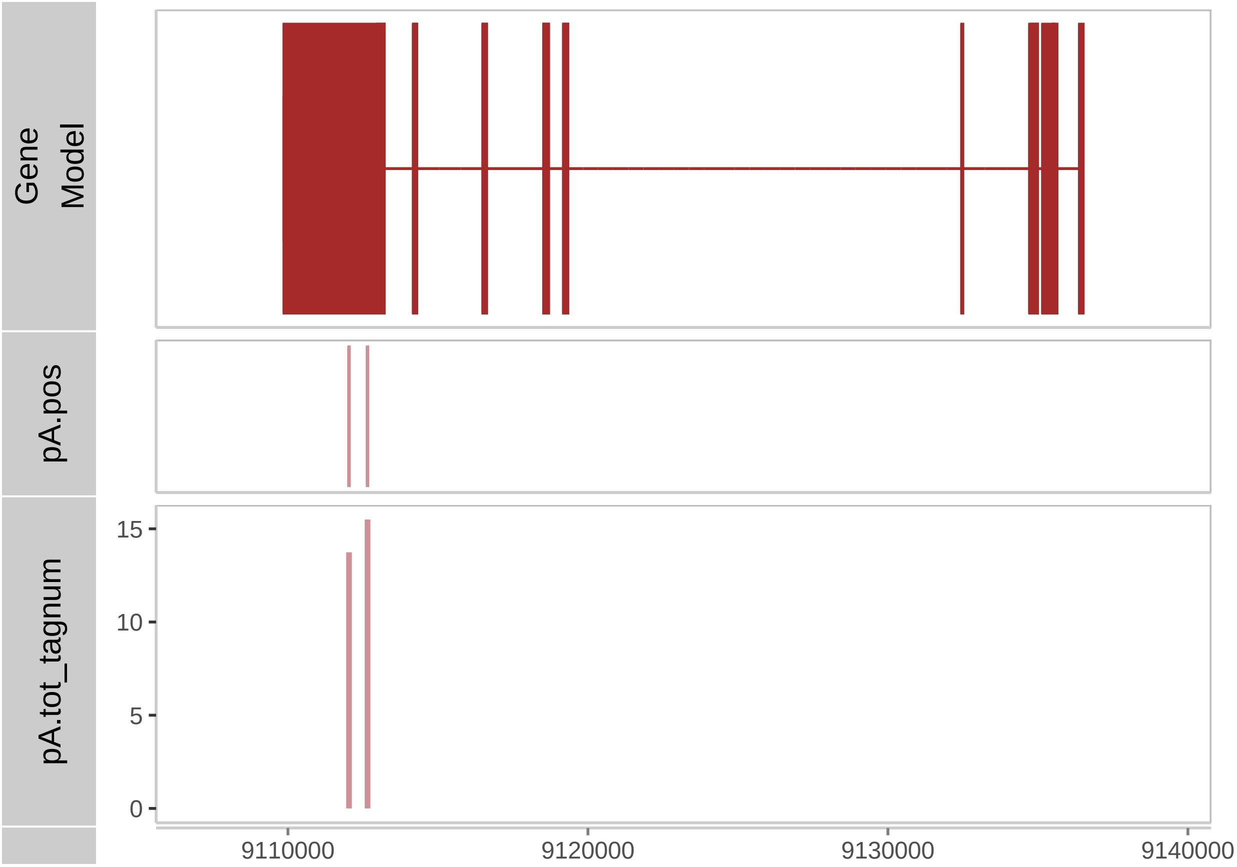

Plot multiple pA datasets

If we have multiple PACdatasets (e.g., one for bulk and one for single cell), we can plot coordinates and expression values of each PACdataset as individual tracks by adding the PACdataset in `PACds.list`. In the following example, for each PACdataset, both the coordinate and the expression value of each PACdataset will be shown.

```
# here we just use a replicated PACds for demonstration
# we can save the tracks and then plot
tks=vizTracks(genomicRegion=genesGR,
          bams=NULL, PACds.list=list(pA1=scPACds, PA2=scPACds),
          PA.show=c("pos", "tot_tagnum"),
          annoSource=annoSource,
          PA.columns="coord", PA.width=10, logPA=TRUE,
          space5=1000, space3=1000,
          vizTheme=NULL, res='list')
```

```
## Plot tracks for region: chr19:-:9108868:9136636
## Get gene model track from annoSource[ orgdb ]...
## Get PACds track...
## chr19:-:9108868:9136636
## Get PACds track...
## chr19:-:9108868:9136636
```

```r
# plot tracks
ggbio::tracks(tks)
```

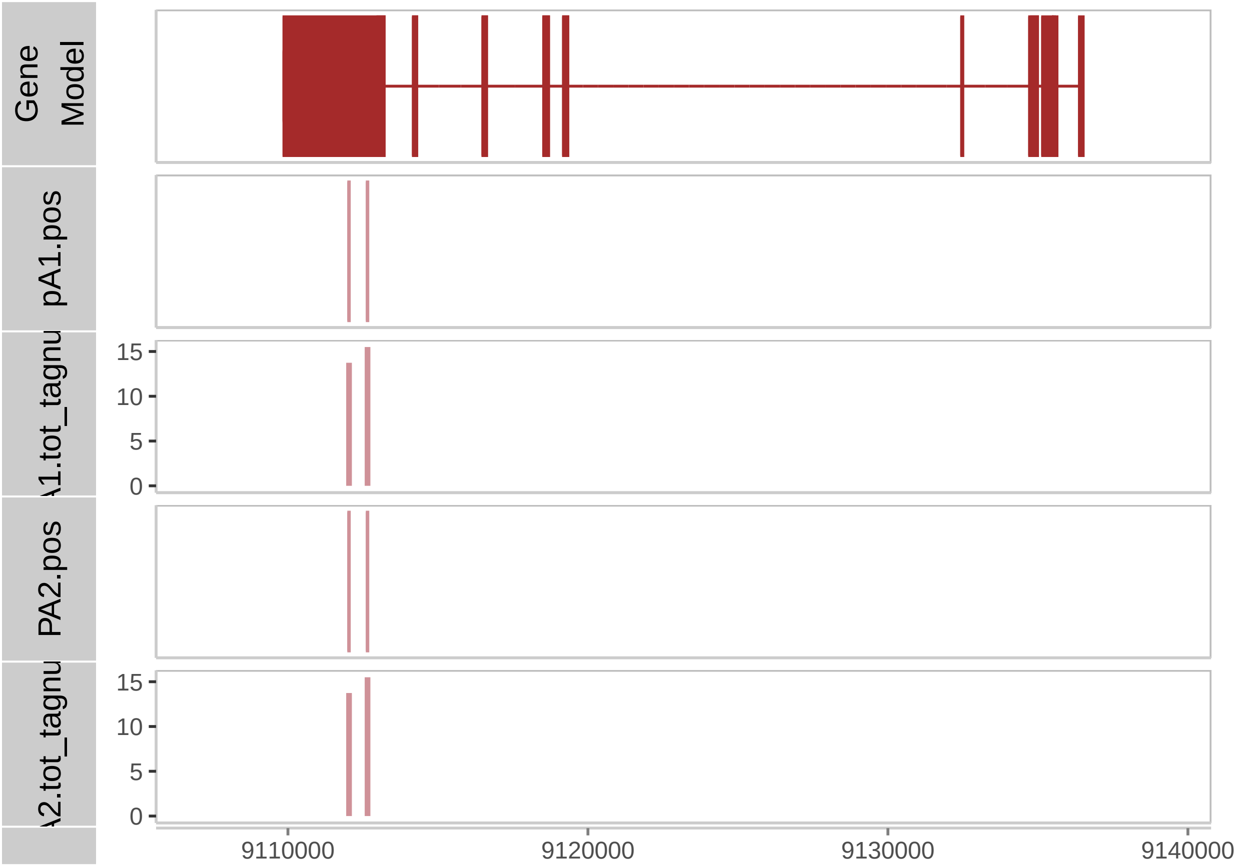


```r
# we can change the title of the tracks
names(tks)[2:5]=c('pos1', 'tag1', 'pos2', 'tag2')
ggbio::tracks(tks)
```

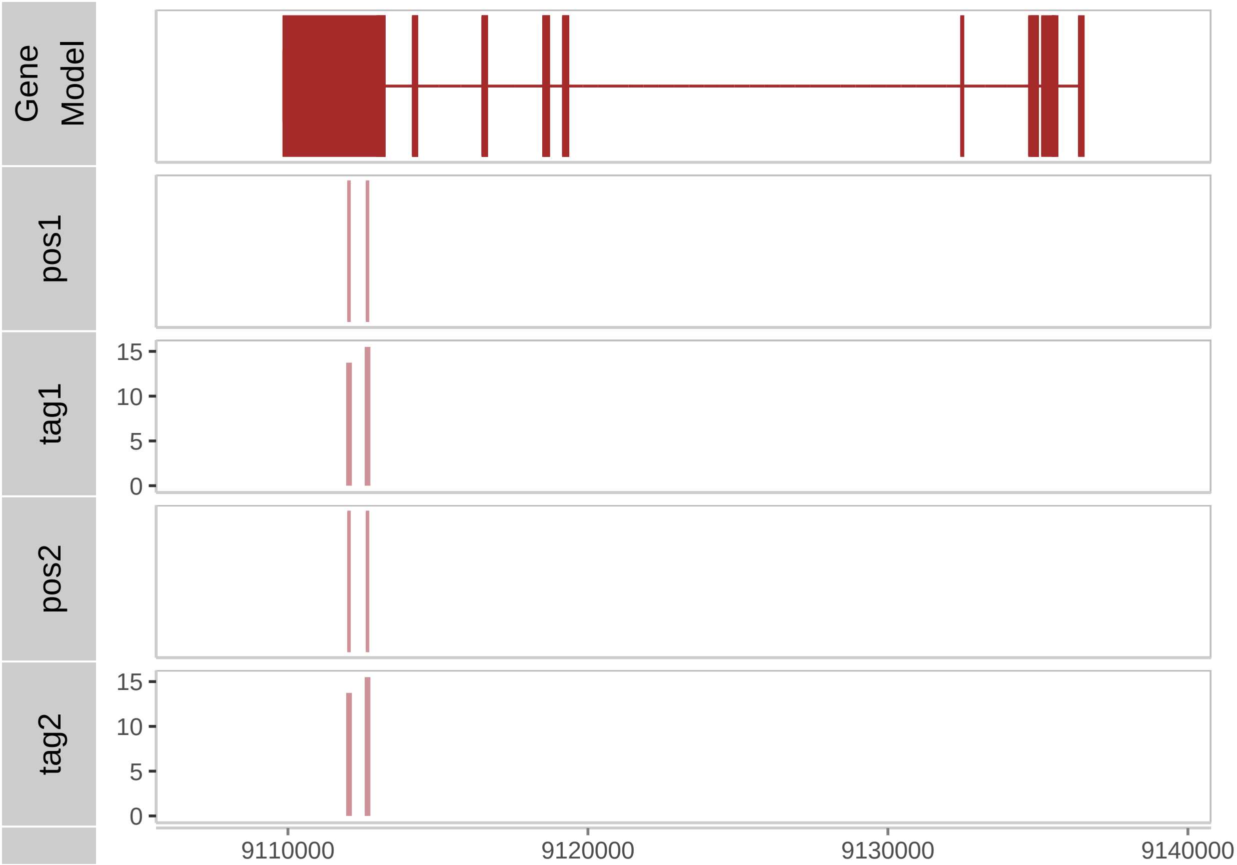

Change colors by `vizTheme`

The `vizTHEME` variable contains all settings for the track plot, which can be customized. Here we changed the colors of pAs.

```
vizTheme=list(PA.col='green', PA.fill='green')
vizTracks(gene=gene,
          bams=NULL, PACds.list=list(pA1=scPACds), PA.show=c("pos"),
          annoSource=annoSource,
          PA.columns="coord", PA.width=10,
          space5=1000, space3=1000,
          vizTheme=vizTheme)
```

```
## Plot tracks for region: chr19:-:9108868:9136636
## Get gene model track from annoSource[ orgdb ]...
## Get PACds track...
## chr19:-:9108868:9136636
```

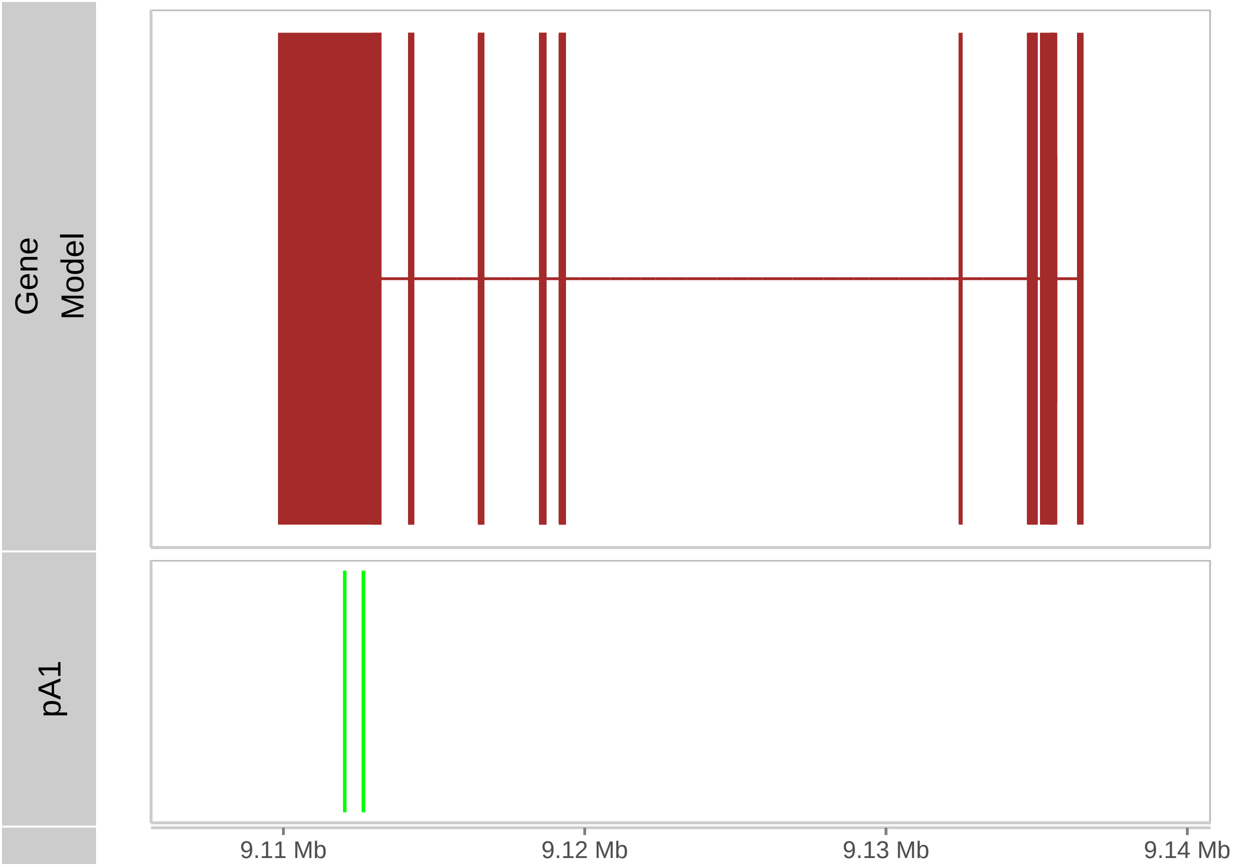

Customize the cells track

Once set `cells=TRUE` in `vizTracks`, a track would be plot to show pA counts or ratios in individual cells. The bar width of each pA depends on `PA.columns` and/or `PA.width`. If `PA.columns` is like 'coord' then the bar width is 2*PA.width+1; if `PA.columns` is like 'start:end', then the bar width of a pA is `end-start+1` of the respective pA.

By default, the cells are sorted within the group, but we can also do not sort the cells or sort the cells within all cells. We can also customize the colors of the values and cell annotations.

Plot expression levels for each cell

```
# use diffusion map to sort cells within each group
vizTracks(genomicRegion=genesGR,
          PACds.list=scPACds, PA.show=c("pos"),
          annoSource=annoSource,
          cells=TRUE, cells.group='celltype',
          cells.method='diff', cells.sort='group',
          cells.width=100,
          PA.columns="coord", PA.width=10)
```

```
## Plot tracks for region: chr19:-:9108868:9136636
## Get gene model track from annoSource[ orgdb ]...
```

```
## Get PACds track...
## chr19:-:9108868:9136636
## Get cells track...
```

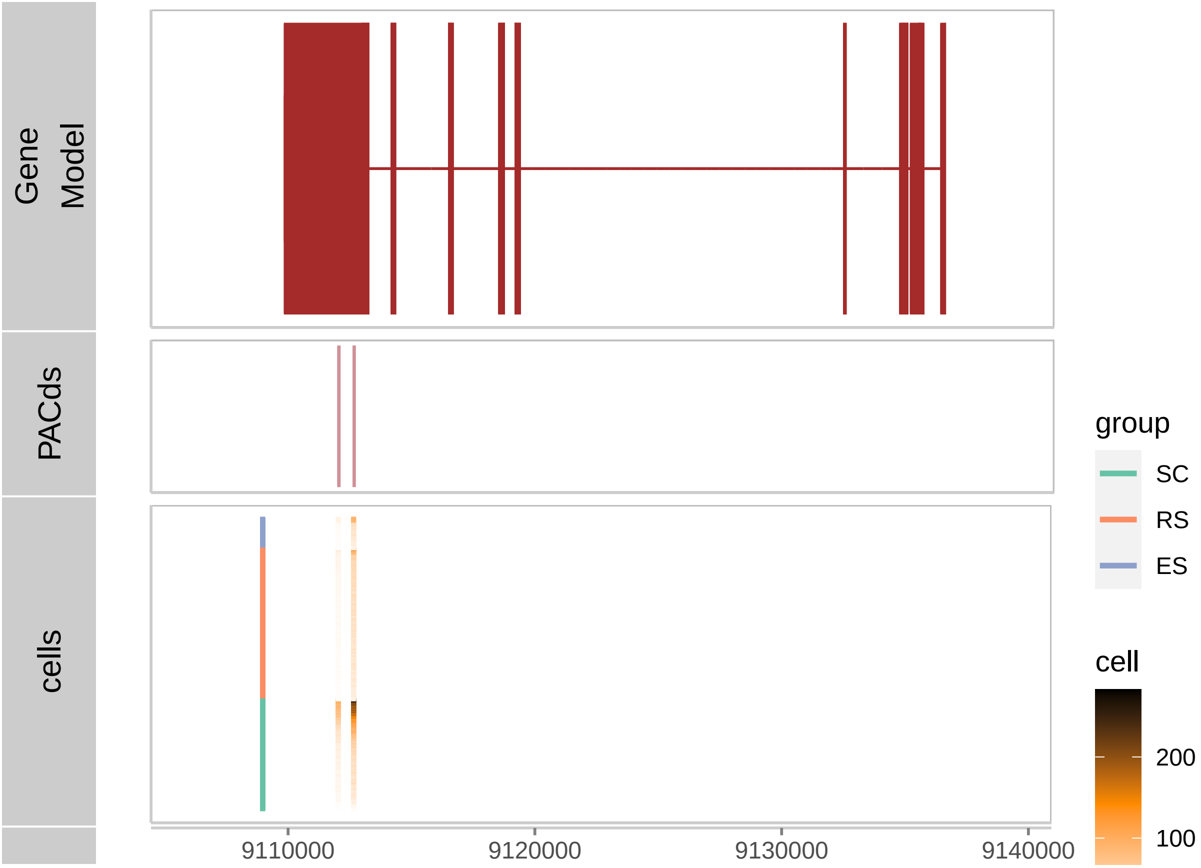

Plot log2 level

The pA expression of one pA is too large to show clearly the expression values for other pAs, here we log2 the value. We can also sort cells by the total counts or ratios of each cell.

```
vizTracks(genomicRegion=genesGR,
          PACds.list=scPACds, PA.show=c("pos"),
          annoSource=annoSource,
          cells=TRUE, cells.group='celltype',
          cells.method='sum', cells.sort='group',
          cells.width=100,
          PA.columns="coord", PA.width=10, logPA=TRUE)
```

```
## Plot tracks for region: chr19:-:9108868:9136636
## Get gene model track from annoSource[ orgdb ]...
## Get PACds track...
## chr19:-:9108868:9136636
## Get cells track...
```

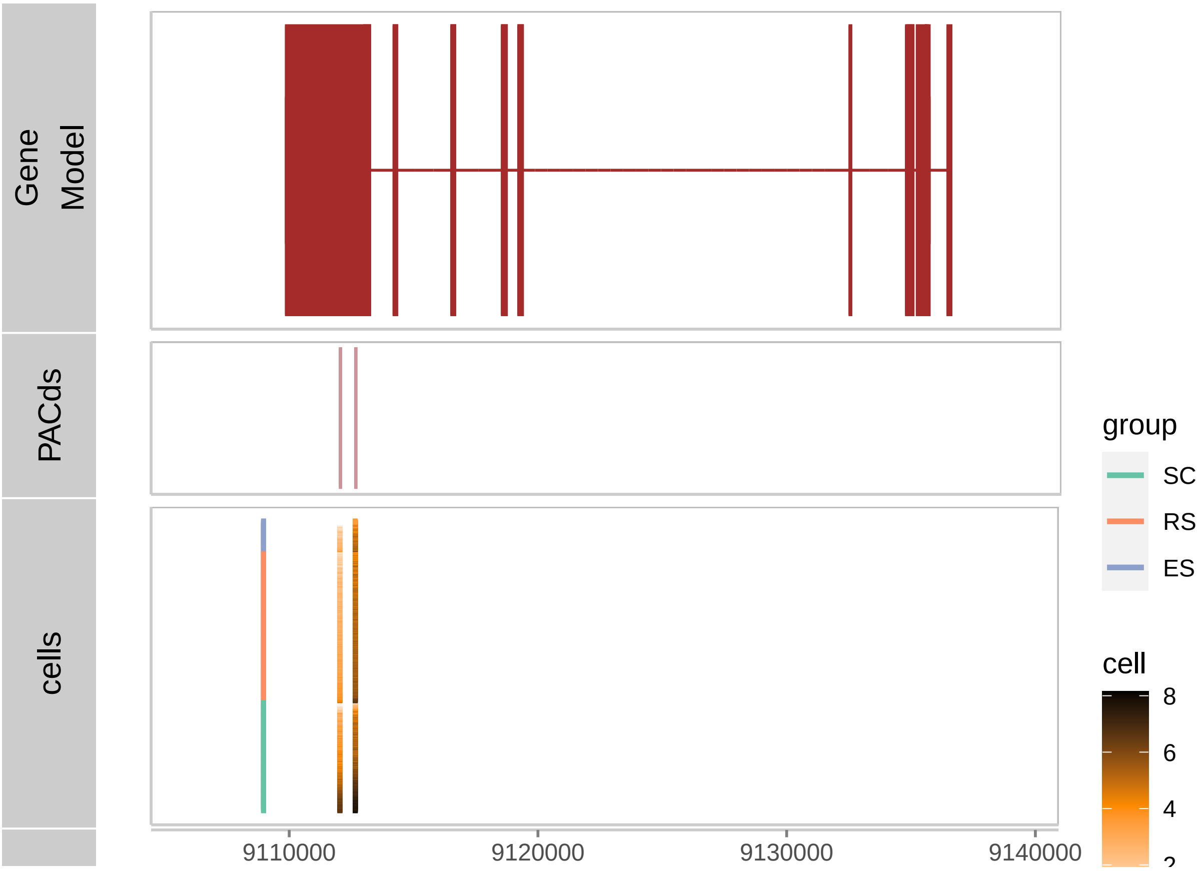

Change cell annotation bar

We can change the position of the cell annotation bar to the right, and also change the colors of the value bar.

```
vizTheme=list(cells.annoBar.pos='right',
              cells.scale.low='white',
              cells.scale.high='purple', cells.scale.mid = "grey",
              cells.group.cols=c(RColorBrewer::brewer.pal(8, "Set1")))

vizTracks(genomicRegion=genesGR,
          PACds.list=scPACds, PA.show=c("pos"),
          annoSource=annoSource,
          cells=TRUE, cells.group='celltype',
          cells.method='sum', cells.sort='group',
          cells.width=100,
          PA.columns="coord", PA.width=10, logPA=TRUE, vizTheme=vizTheme)
```

```
## Plot tracks for region: chr19:-:9108868:9136636
## Get gene model track from annoSource[ orgdb ]...
## Get PACds track...
## chr19:-:9108868:9136636
## Get cells track...
```

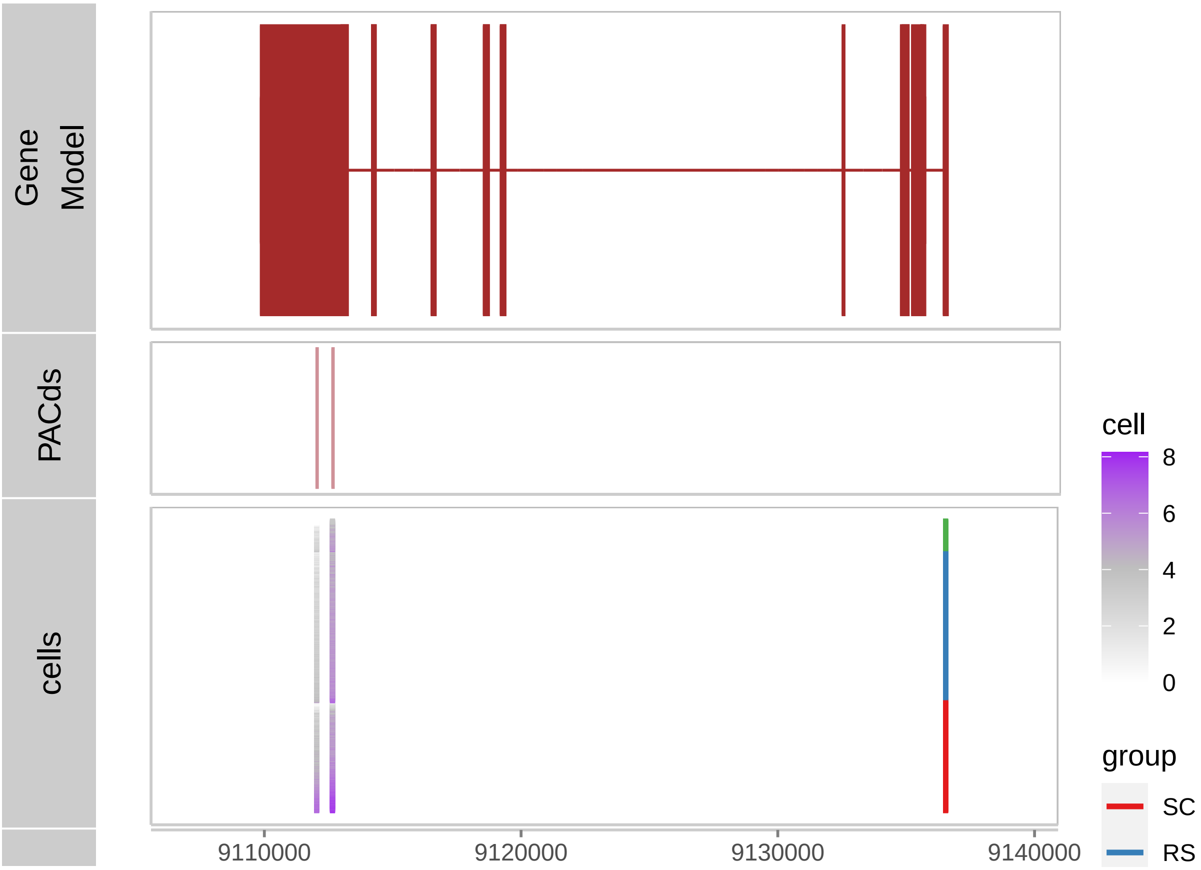

We can also put the annotation bar in a specific position.

```
vizTracks(genomicRegion=genesGR,
          PACds.list=scPACds, PA.show=c("pos"),
          annoSource=annoSource,
          cells=TRUE, cells.group='celltype',
          cells.method='sum', cells.sort='group',
          cells.width=100, cells.annoCoord=9110000,
          PA.columns="coord", PA.width=10, logPA=TRUE)
```

```
## Plot tracks for region: chr19:-:9108868:9136636
## Get gene model track from annoSource[ orgdb ]...
## Get PACds track...
## chr19:-:9108868:9136636
## Get cells track...
```

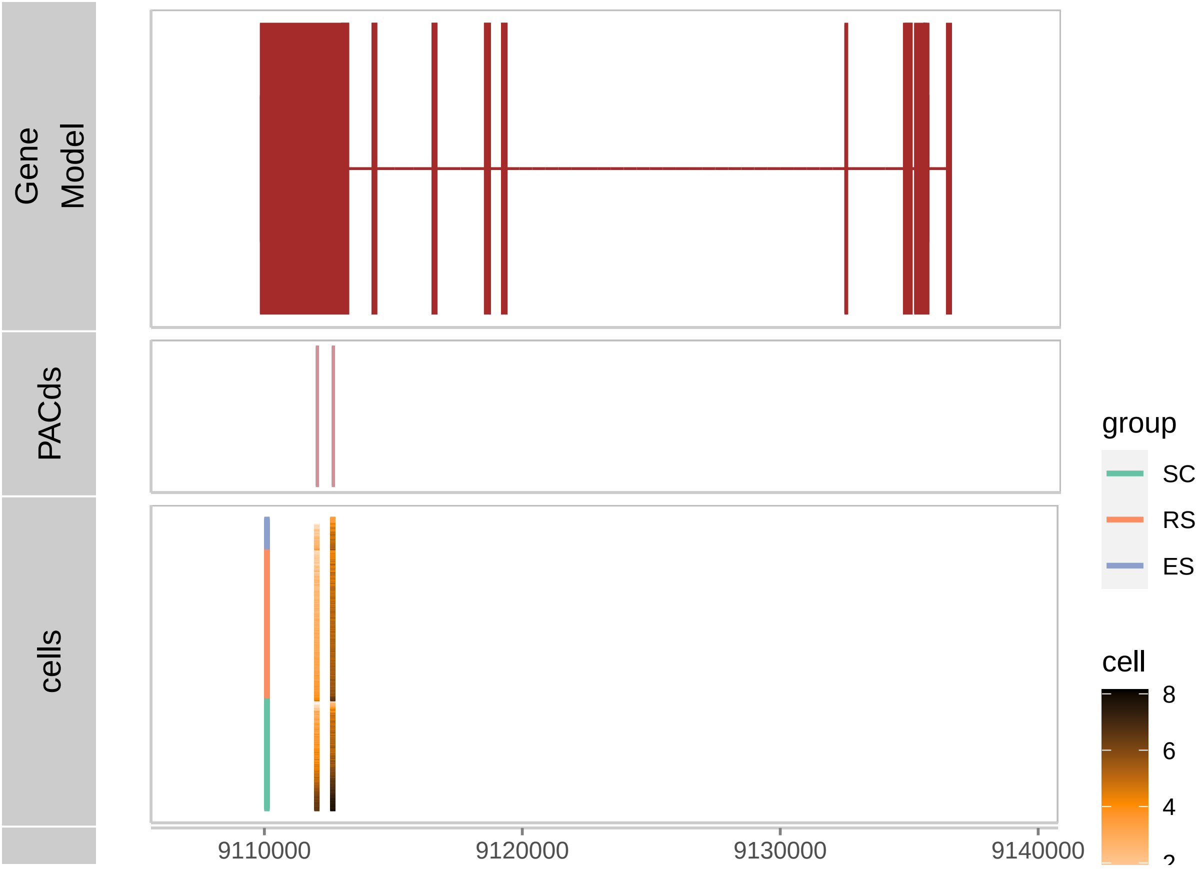

Sort cells

By default, the cells are sorted within the group, we can also do not sort the cells.

```
vizTracks(genomicRegion=genesGR,
          PACds.list=scPACds, PA.show=c("pos"),
          annoSource=annoSource,
          cells=TRUE, cells.group='celltype',
          cells.method='sum', cells.sort='none',
          cells.width=100,
          PA.columns="coord", PA.width=10, logPA=TRUE)
```

```
## Plot tracks for region: chr19:-:9108868:9136636
## Get gene model track from annoSource[ orgdb ]...
## Get PACds track...
## chr19:-:9108868:9136636
## Get cells track...
```

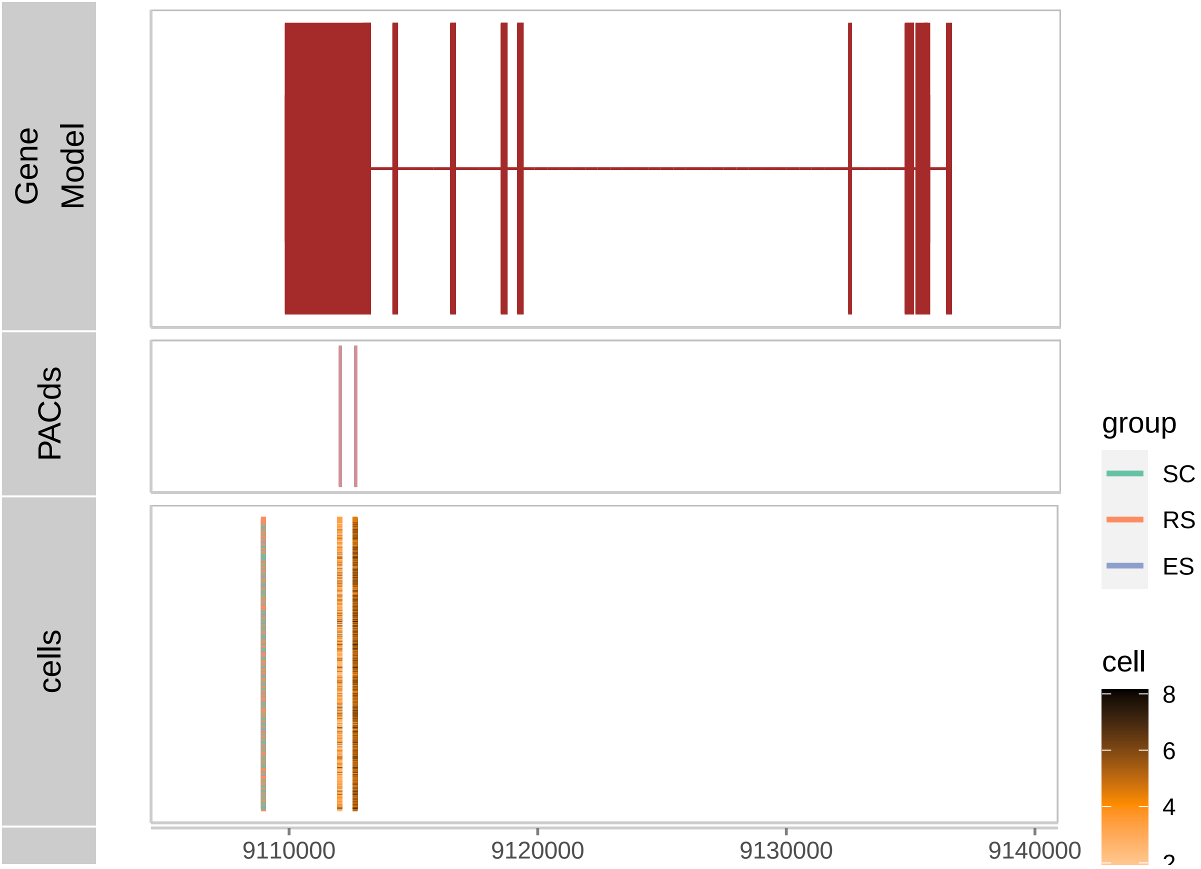

We can also sort the cells within all cells rather than within each group.

```
vizTracks(genomicRegion=genesGR,
          PACds.list=scPACds, PA.show=c("pos"),
          annoSource=annoSource,
          cells=TRUE, cells.group='celltype',
          cells.method='sum', cells.sort='all',
          cells.width=100,
          PA.columns="coord", PA.width=10, logPA=TRUE)
```

```
## Plot tracks for region: chr19:-:9108868:9136636
## Get gene model track from annoSource[ orgdb ]...
## Get PACds track...
## chr19:-:9108868:9136636
## Get cells track...
```

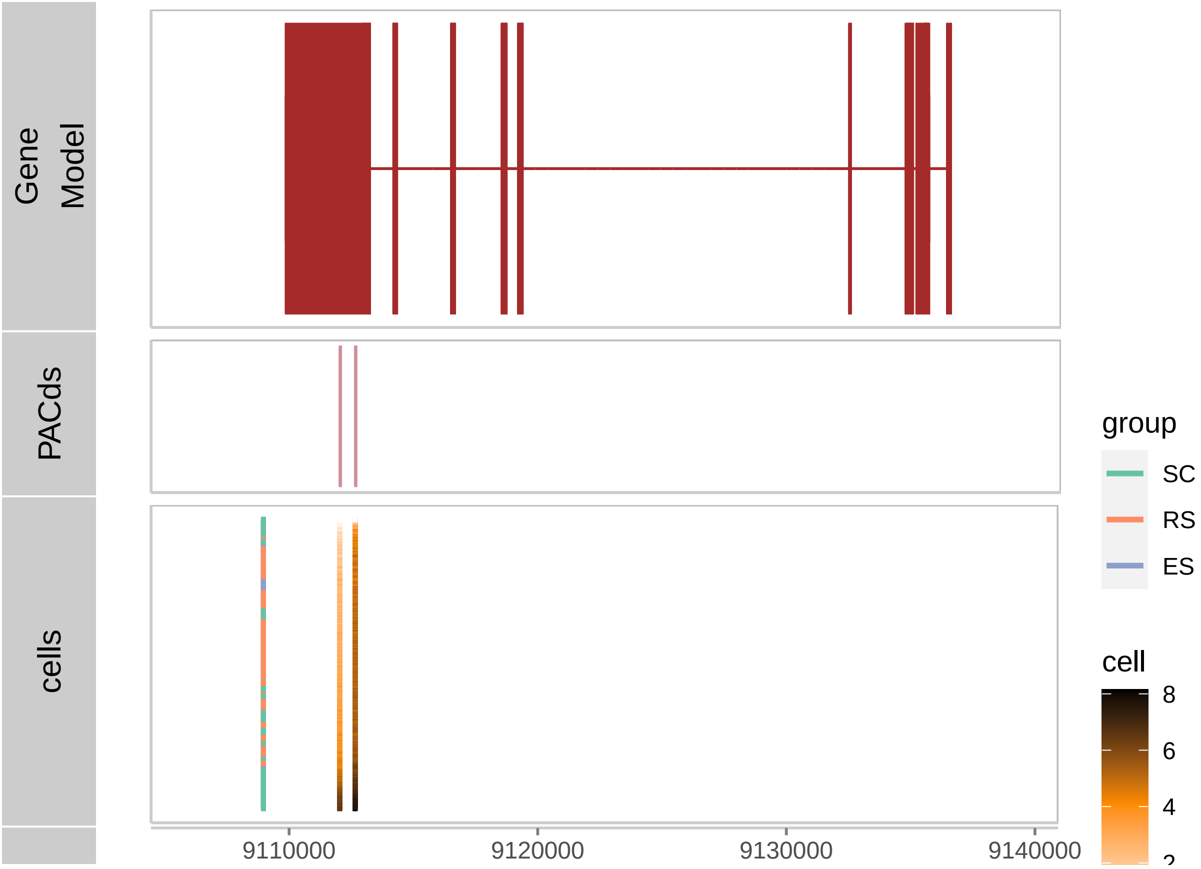

Customize BAM tracks

Plot BAM coverage

By default, BAM coverage of individual BAM files listed in `bams` will be plot. Each BAM could be one cell type. We can also add additional track showing merged (average or sum) coverage from all BAM files, by setting `vizTheme`.

```
# set how to show BAM coverage
vizTheme=list(bam.covMerge='avg',
              bam.showMerge=TRUE,
              bam.showSingle=TRUE,
              bam.covMerge.col='yellow',
              bam.covMerge.fill='yellow')

# we can also set the colors for individual bam coverages
vizTheme$bams.col=RColorBrewer::brewer.pal(8, "Set1")
vizTheme$bams.fill=RColorBrewer::brewer.pal(8, "Set1")

vizTracks(gene=gene,
          bams=bams,
          PACds.list=list(pA1=scPACds), PA.show=c("pos"),
          annoSource=annoSource,
          PA.columns="coord", PA.width=10,
```

```
         space5=1000, space3=1000,
         vizTheme=vizTheme)
```

```
## Plot tracks for region: chr19:-:9108868:9136636
## Get gene model track from annoSource[ orgdb ]...
## Get PACds track...
## chr19:-:9108868:9136636
## Get BAM tracks...
```

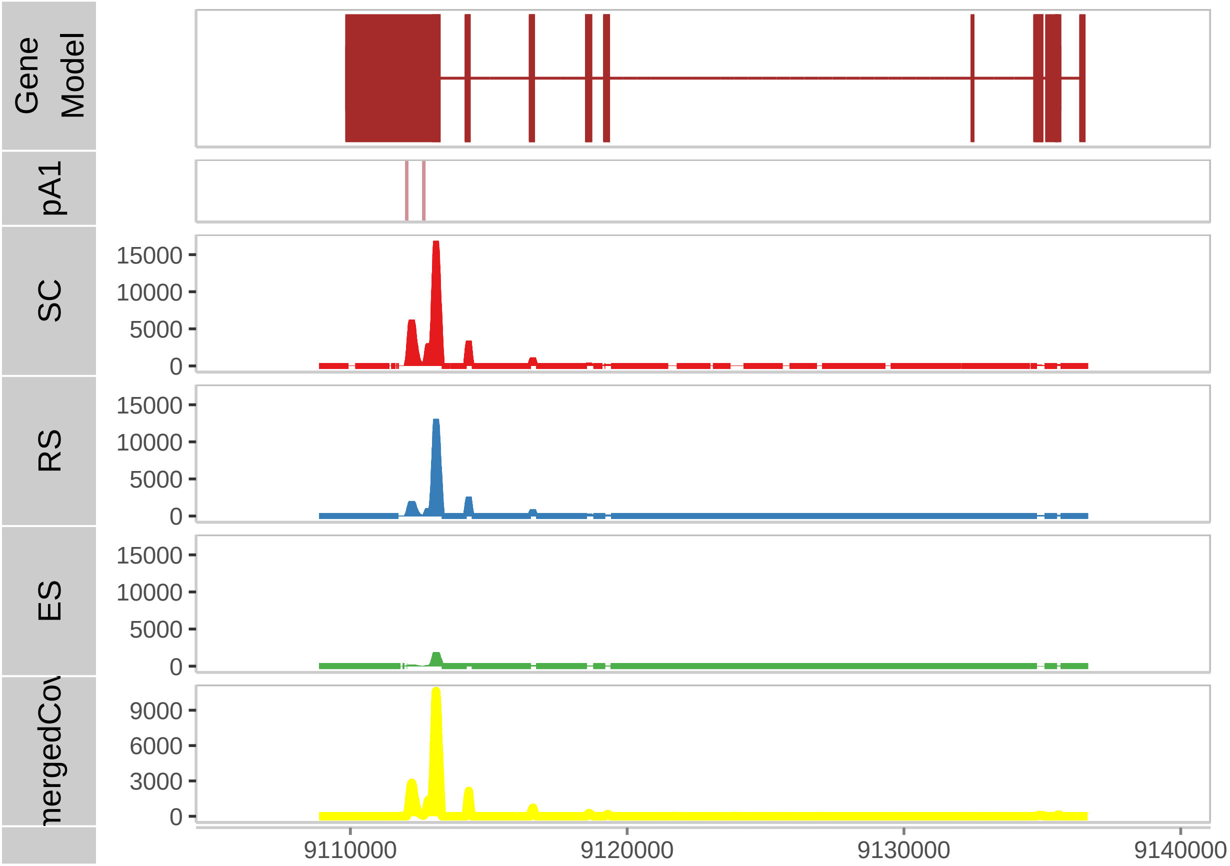

Collapse BAM coverage

We can also collapse all BAM coverage into one track by setting `bam.collapse` in `vizTheme` as TRUE. In this case, BAM coverage of individual BAM files will be shown as curves in one track.

```
vizTheme$bam.collapse=TRUE

vizTracks(gene=gene,
          bams=bams,
          PACds.list=list(pA1=scPACds), PA.show=c("pos"),
          annoSource=annoSource,
          PA.columns="coord", PA.width=10,
          space5=1000, space3=1000,
          vizTheme=vizTheme)
```

```
## Plot tracks for region: chr19:-:9108868:9136636
## Get gene model track from annoSource[ orgdb ]...
## Get PACds track...
## chr19:-:9108868:9136636
## Get BAM tracks...
```

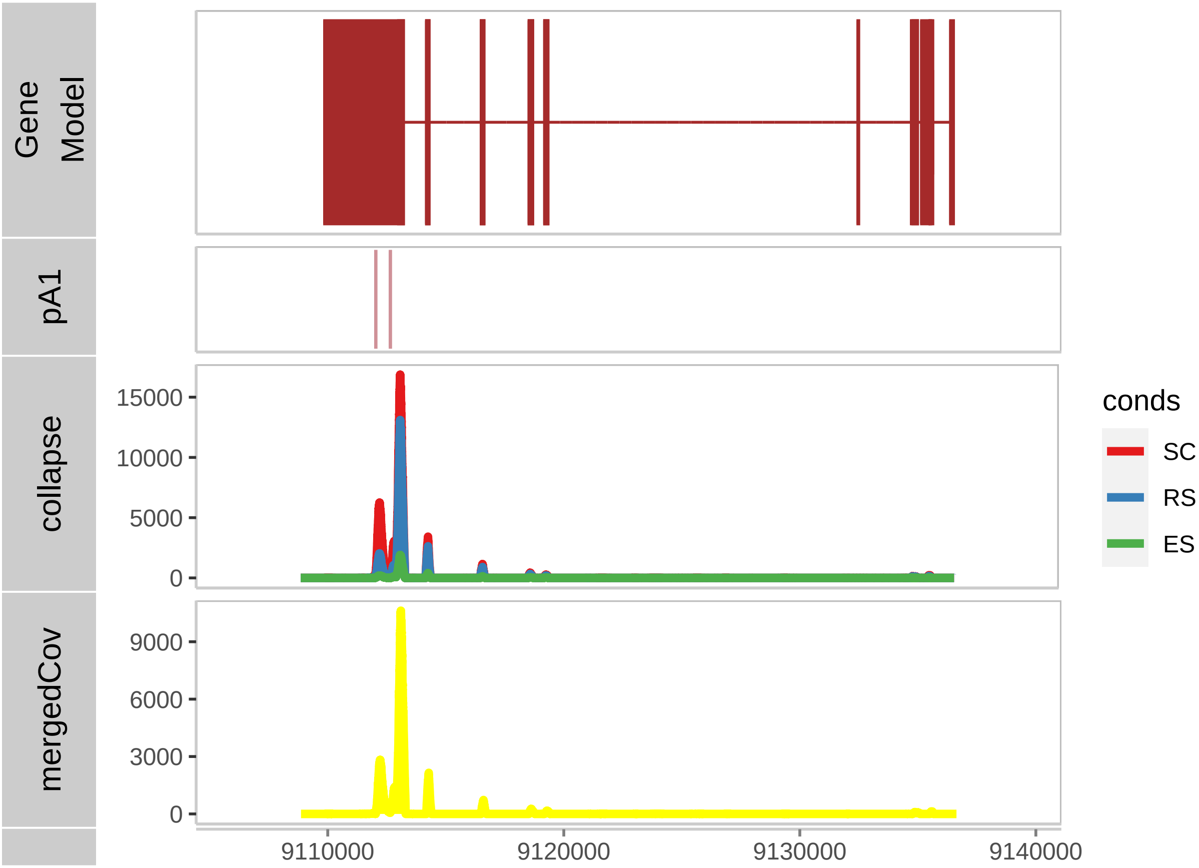

Customize the gene model track

Plot a gene model

vizAPA can retrieve gene model track from a given gene or a genomic range, based on different annotation sources, by setting `annoSource`. Here is an example to get gene model from a TxDb object. Before start, we check whether the chromosome names in this TxDb object are consistent with PACdataset and BAM files.

```
## yes, they are consistent
isChrConsistent(annoSource['txdb'], bams, scPACds, exact=FALSE)
```

```
## [1] TRUE
```

```
head(getChrs(annoSource['txdb']))
```

```
## [1] "chr1" "chr2" "chr3" "chr4" "chr5" "chr6"
```

```r
## set the default anno as txdb
annoSource=setDefaultAnno(annoSource, 'txdb')
```

```r
vizTracks(gene=gene,
          bams=bams,
          PACds.list=list(pA=scPACds), PA.show=c("pos"),
          annoSource=annoSource,
          PA.columns="coord", PA.width=10,
          space5=1000, space3=1000)
```

```
## Plot tracks for region: chr19:-:9108868:9136636
## Get gene model track from annoSource[ txdb ]...
## Get PACds track...
## chr19:-:9108868:9136636
## Get BAM tracks...
```

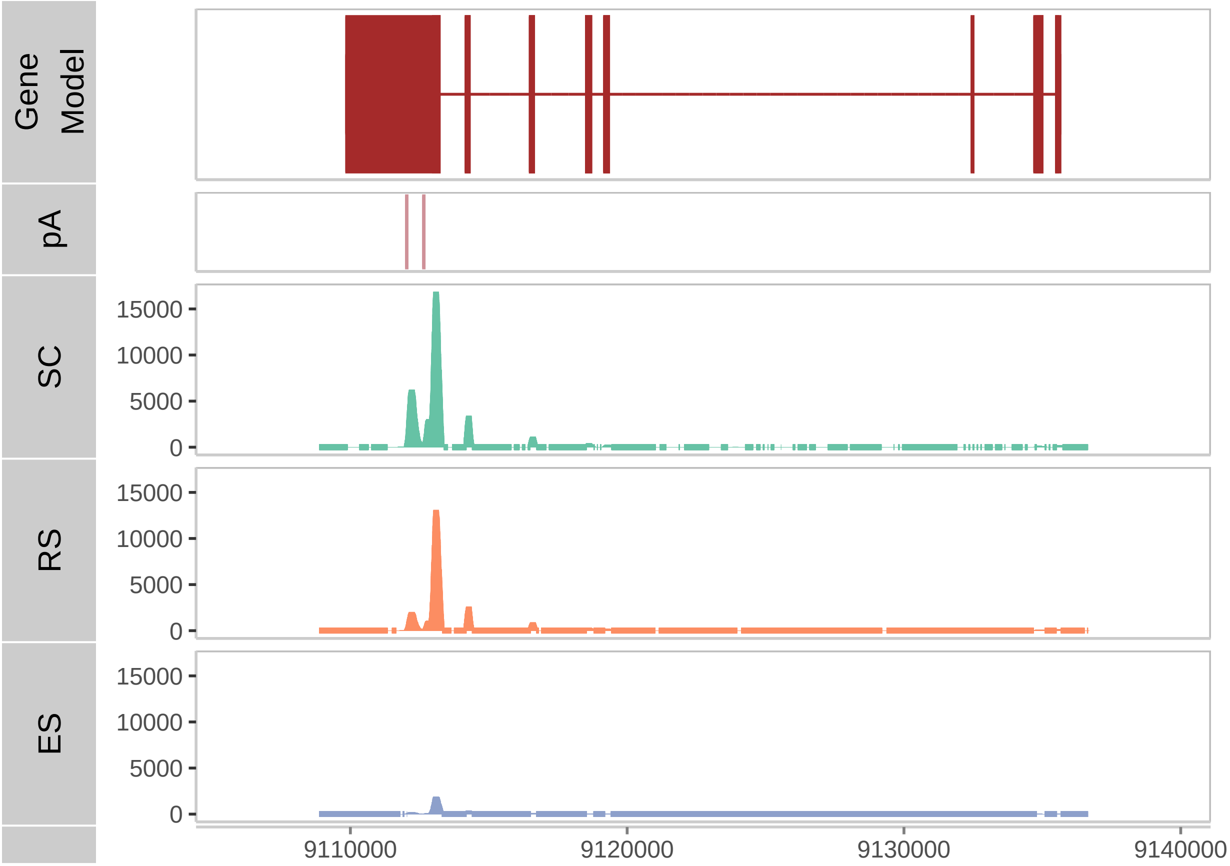

Plot a genomic region

We can also plot the genomic region instead of gene model, without providing any annotations, i.e., just providing an empty annoSource.

```r
vizTracks(gene=gene,
          bams=bams,
```

```r
PACds.list=list(pA=scPACds), PA.show=c("pos"),
annoSource=new('annoHub'),
PA.columns="coord", PA.width=10,
space5=1000, space3=1000)
```

```
## Cannot find any range for gene [66514] from annoSource, try to get gene-range from PACds.list.
## Found gene region for gene [66514] in PACds: chr19|-|91098689135636
## Plot tracks for region: chr19:-:9108868:9136636
## Get gene model track from annoSource[  ]...
## Get PACds track...
## chr19:-:9108868:9136636
## Get BAM tracks...
```

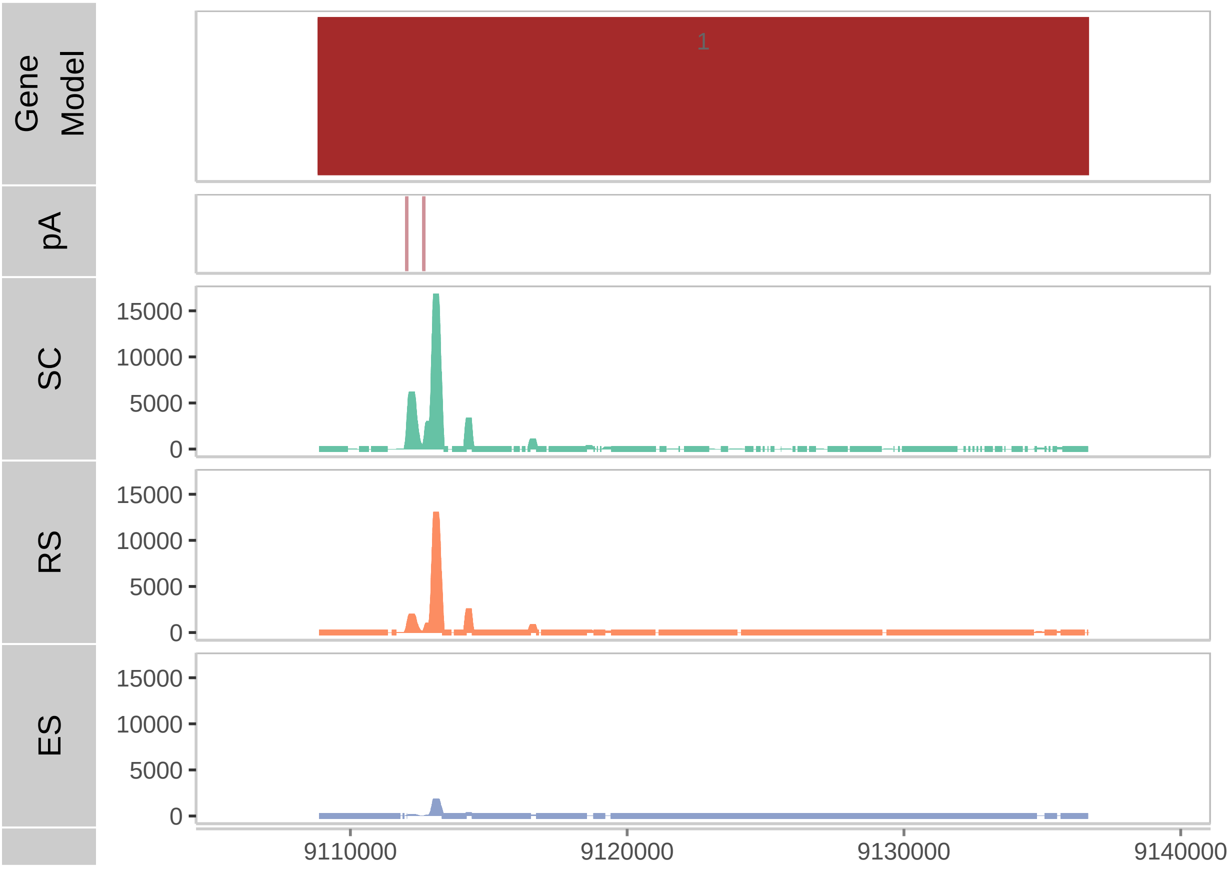

Plot tracks mannually

The `vizTracks` function provide a wrapper function to plot BAMs, pAs, gene model, individual cells together. However, we can also use a series of `getTrack...` functions to get gene model tracks from different genome annotation sources, get pA tracks from different PACdatasets, get pA in individual cells, and get BAM coverage tracks from different BAM files, respectively. And then we can combine all these tracks together in one plot.

Get gene model tracks

```
# first check whether the gene is annotated in different annos.
getGenesRange(genes=gene, annoSource['txdb'], rt='gr')
getGenesRange(genes=gene, annoSource['ensdb'], rt='gr')
getGenesRange(genes=gene, annoSource['orgdb'], rt='gr')
getGenesRange(genes=gene, annoSource['biomart'], rt='gr')
getGenesRange(genes=gene, annoSource['genes'], rt='gr')
```

```
# collapose gene model features, not show transcript labels
vizTheme=list(gm.reduce=FALSE, gm.label=FALSE)

# use the orgdb anno
annoSource=setDefaultAnno(annoSource, 'orgdb')
gm.tk1=getTrackGeneModel(gene=gene, annoSource=annoSource, title='orgdb',
                         vizTheme = vizTheme)

annoSource=setDefaultAnno(annoSource, 'ensdb')
gm.tk2=getTrackGeneModel(gene=gene, annoSource=annoSource,
                         title='ensdb', vizTheme = vizTheme)

annoSource=setDefaultAnno(annoSource, 'txdb')
gm.tk3=getTrackGeneModel(gene=gene, annoSource=annoSource,
                         title='txdb', vizTheme = vizTheme)

annoSource=setDefaultAnno(annoSource, 'genes')
gm.tk4=getTrackGeneModel(gene=gene, annoSource=annoSource,
                         title='genes', vizTheme = vizTheme)

#annoSource=setDefaultAnno(annoSource, 'biomart')
#gm.tk5=getTrackGeneModel(gene=gene, annoSource=annoSource, title='biomart')
```

```
# show these different annotations for the same gene
tks=c(gm.tk1, gm.tk2, gm.tk3, gm.tk4)
ggbio::tracks(tks, heights=c(2, 3, 3, 1.5))
```

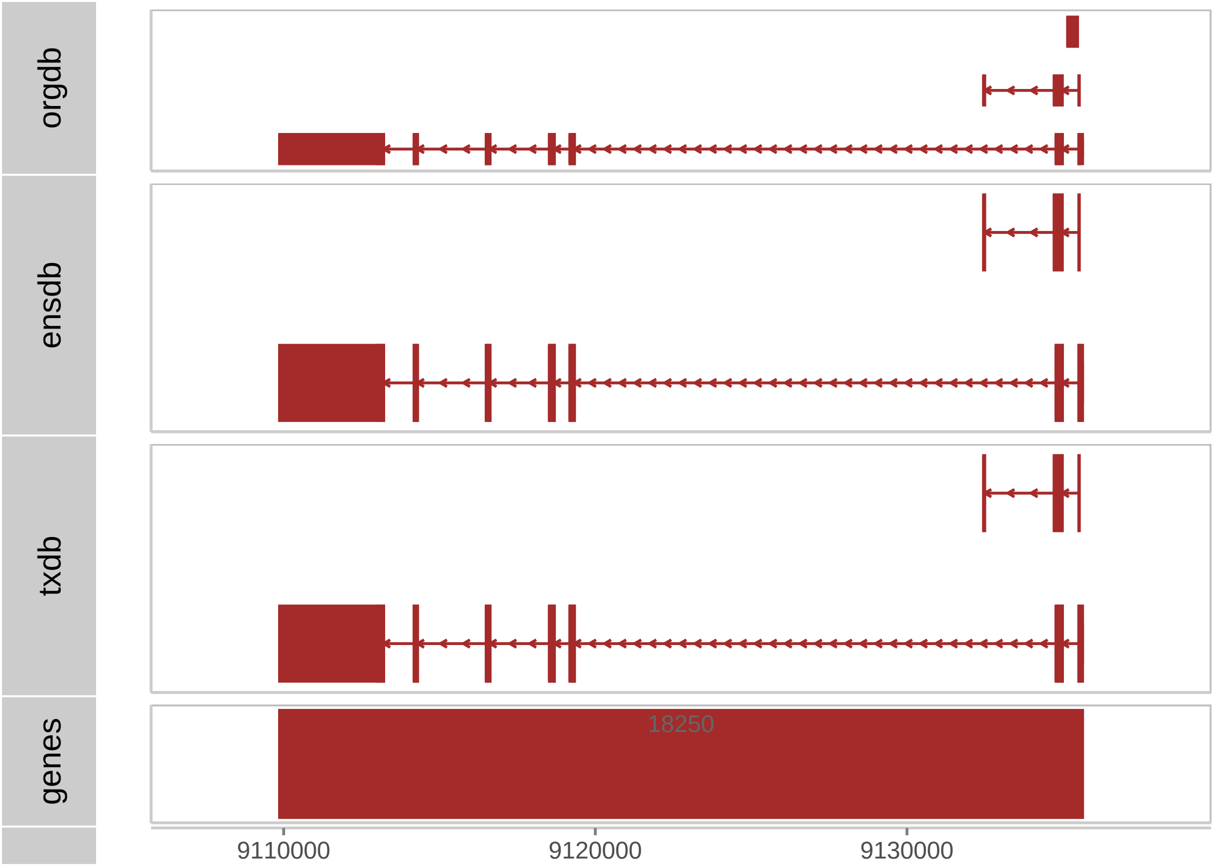

Get pA/cells/BAM tracks

We can also get pA or cells tracks for PACdatasets.

```
pac.tk1=getTrackPACds(scPACds, gene=gene,
                      PA.show=c("pos"),
                      PA.columns="coord", PA.width=10,
                      title='PApos', vizTheme = list(PA.shape=17))
```

```
pac.tk2=getTrackPACds(scPACds, gene=gene,
                      PA.show=c("tot_tagnum"), log=TRUE,
                      PA.columns="start:end", title='totTag')
```

```
sc.tk1=getTrackCells(scPACds,gene=gene,
                     group='celltype',
                     sortMethod='sum', sortCells='group',log=TRUE,
                     PA.columns="coord", PA.width=100, title='diffs')

genomicRegion=getGenesRange(genes=gene, annoObj=annoSource['orgdb'], rt='str')
bam.tk1=getTrackBams(bams, genomicRegion=genomicRegion)

## show gene model, pAs, cells, and bams
tks=c(gm.tk1, pac.tk1, pac.tk2, sc.tk1 ,bam.tk1)
ggbio::tracks(tks)
```

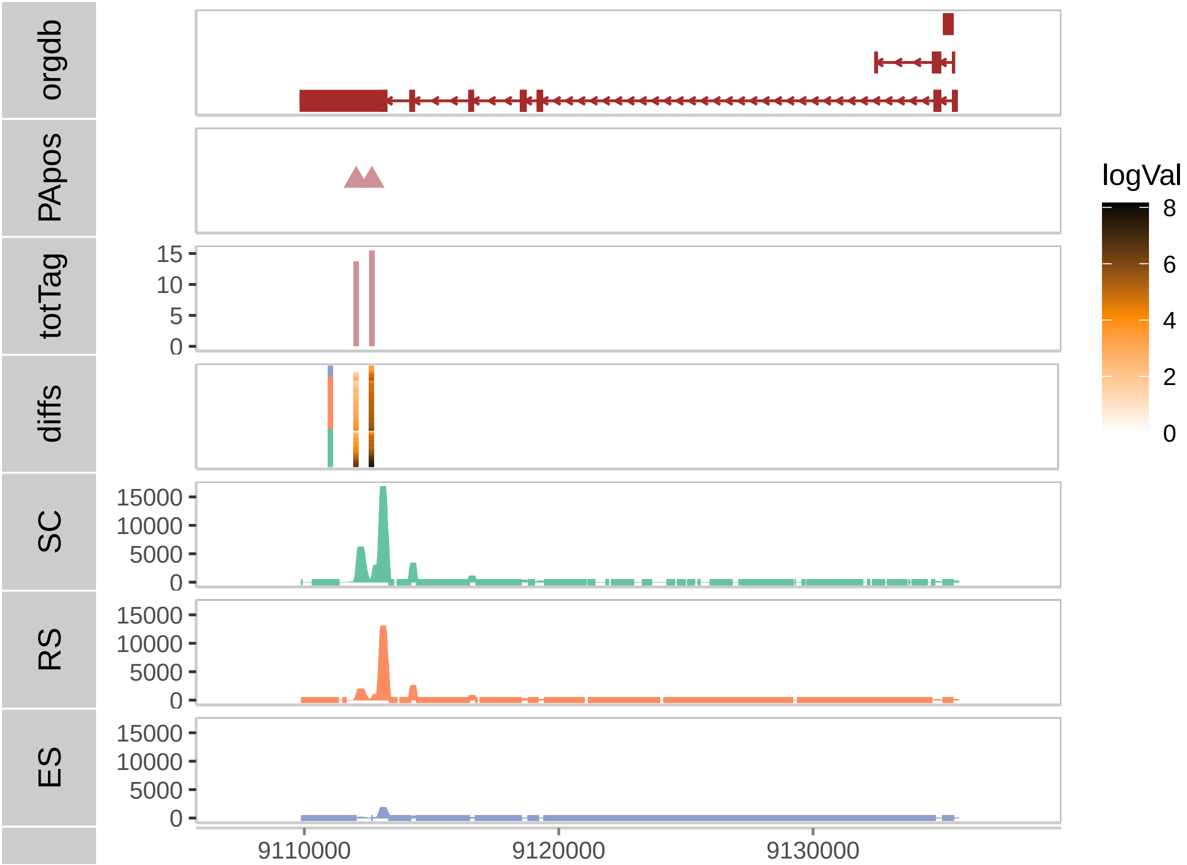

We can zoom in on a genomic region by specifying `xlim` and set the height of each track. For example, here we only display the 3'UTR area.

```
# plot legend 'horizontal' or 'vertical'
ggbio::tracks(tks, heights=c(0.8, 0.5, 1.2, 1.2, 1.2, 1.2, 1.2),
              xlim=c(9111000, 9113500))+
  ggplot2::theme(legend.direction = 'vertical')
```

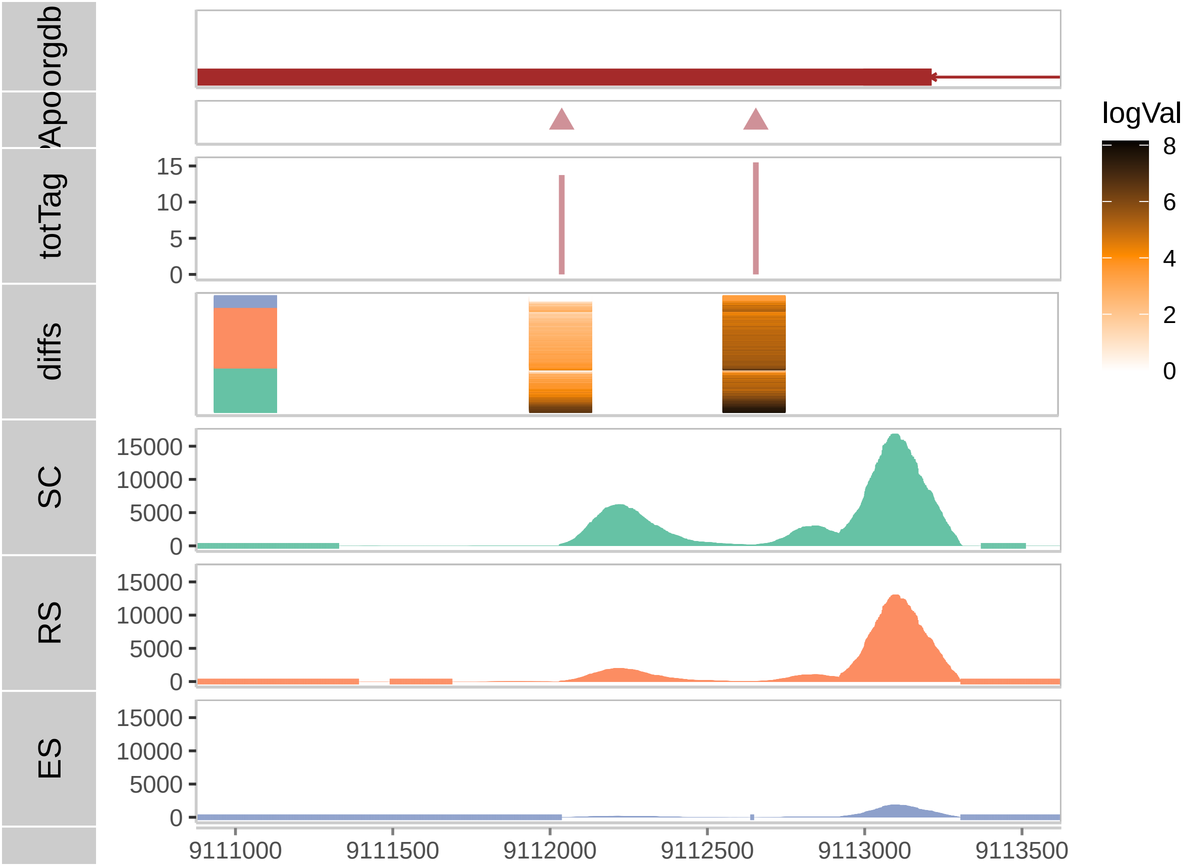

Session information

The session information records the versions of all the packages used in the generation of the present document.

```
sessionInfo()
```

```
## R version 4.2.2 (2022-10-31 ucrt)
## Platform: x86_64-w64-mingw32/x64 (64-bit)
## Running under: Windows 10 x64 (build 22621)
##
## Matrix products: default
##
## locale:
## [1] LC_COLLATE=Chinese (Simplified)_China.utf8
## [2] LC_CTYPE=Chinese (Simplified)_China.utf8
## [3] LC_MONETARY=Chinese (Simplified)_China.utf8
## [4] LC_NUMERIC=C
## [5] LC_TIME=Chinese (Simplified)_China.utf8
##
## attached base packages:
## [1] stats4    stats     graphics  grDevices utils     datasets  methods
## [8] base
##
```

```
## other attached packages:
##  [1] biomaRt_2.54.0
##  [2] EnsDb.Mmusculus.v79_2.99.0
##  [3] ensembldb_2.22.0
##  [4] AnnotationFilter_1.22.0
##  [5] Mus.musculus_1.3.1
##  [6] TxDb.Mmusculus.UCSC.mm10.knownGene_3.10.0
##  [7] org.Mm.eg.db_3.16.0
##  [8] GO.db_3.16.0
##  [9] OrganismDbi_1.40.0
## [10] GenomicFeatures_1.50.2
## [11] GenomicRanges_1.50.1
## [12] GenomeInfoDb_1.34.9
## [13] AnnotationDbi_1.60.0
## [14] IRanges_2.32.0
## [15] S4Vectors_0.36.0
## [16] Biobase_2.58.0
## [17] BiocGenerics_0.44.0
## [18] vizAPA_0.1.0
##
## loaded via a namespace (and not attached):
##   [1] utf8_1.2.2                  tidyselect_1.2.0
##   [3] movAPA_0.2.0                RSQLite_2.2.18
##   [5] htmlwidgets_1.5.4           ranger_0.14.1
##   [7] grid_4.2.2                  BiocParallel_1.32.1
##   [9] munsell_0.5.0               destiny_3.12.0
##  [11] codetools_0.2-18            interp_1.1-3
##  [13] future_1.30.0               withr_2.5.0
##  [15] colorspace_2.0-3            progressr_0.12.0
##  [17] filelock_1.0.2              highr_0.9
##  [19] knitr_1.41                  rstudioapi_0.14
##  [21] SingleCellExperiment_1.20.0 robustbase_0.95-0
##  [23] vcd_1.4-11                  VIM_6.2.2
##  [25] TTR_0.24.3                  listenv_0.9.0
##  [27] MatrixGenerics_1.10.0       labeling_0.4.2
##  [29] GenomeInfoDbData_1.2.9      bit64_4.0.5
##  [31] farver_2.1.1                parallelly_1.33.0
##  [33] vctrs_0.6.5                 generics_0.1.3
##  [35] xfun_0.35                   ggthemes_4.2.4
##  [37] biovizBase_1.46.0           BiocFileCache_2.6.0
##  [39] R6_2.5.1                    doParallel_1.0.17
##  [41] clue_0.3-63                 RcppEigen_0.3.3.9.3
##  [43] bitops_1.0-7                cachem_1.0.6
##  [45] reshape_0.8.9               DelayedArray_0.24.0
##  [47] assertthat_0.2.1            BiocIO_1.8.0
##  [49] scales_1.2.1                nnet_7.3-18
##  [51] gtable_0.3.1                globals_0.16.2
##  [53] ggbio_1.46.0                rlang_1.1.2
##  [55] scatterplot3d_0.3-43        GlobalOptions_0.1.2
##  [57] splines_4.2.2               rtracklayer_1.58.0
##  [59] lazyeval_0.2.2              hexbin_1.28.3
##  [61] dichromat_2.0-0.1           checkmate_2.1.0
##  [63] abind_1.4-5                 BiocManager_1.30.19
##  [65] yaml_2.3.6                  reshape2_1.4.4
```

```
##  [67] backports_1.4.1             Hmisc_5.0-0
##  [69] RBGL_1.74.0                 tools_4.2.2
##  [71] ggplot2_3.4.0               ellipsis_0.3.2
##  [73] RColorBrewer_1.1-3          proxy_0.4-27
##  [75] Rcpp_1.0.9                  plyr_1.8.8
##  [77] base64enc_0.1-3             progress_1.2.2
##  [79] zlibbioc_1.44.0             purrr_1.0.2
##  [81] RCurl_1.98-1.9              prettyunits_1.1.1
##  [83] rpart_4.1.19                deldir_1.0-6
##  [85] GetoptLong_1.0.5            cowplot_1.1.1
##  [87] zoo_1.8-11                  SeuratObject_4.1.3
##  [89] SummarizedExperiment_1.28.0 cluster_2.1.4
##  [91] magrittr_2.0.3              data.table_1.14.6
##  [93] RSpectra_0.16-1             circlize_0.4.15
##  [95] lmtest_0.9-40               pcaMethods_1.90.0
##  [97] ggnewscale_0.4.8            ProtGenerics_1.30.0
##  [99] matrixStats_0.63.0          hms_1.1.2
## [101] evaluate_0.18               smoother_1.1
## [103] XML_3.99-0.12               jpeg_0.1-10
## [105] gridExtra_2.3               shape_1.4.6
## [107] testthat_3.1.5              compiler_4.2.2
## [109] tibble_3.2.1                crayon_1.5.2
## [111] htmltools_0.5.3             Formula_1.2-4
## [113] tidyr_1.2.1                 DBI_1.1.3
## [115] dbplyr_2.2.1                ComplexHeatmap_2.14.0
## [117] MASS_7.3-58.1               rappdirs_0.3.3
## [119] boot_1.3-28                 Matrix_1.5-3
## [121] car_3.1-1                   brio_1.1.3
## [123] cli_3.6.1                   parallel_4.2.2
## [125] pkgconfig_2.0.3             GenomicAlignments_1.34.0
## [127] laeken_0.5.2                foreign_0.8-83
## [129] sp_1.5-1                    xml2_1.3.3
## [131] foreach_1.5.2               XVector_0.38.0
## [133] stringr_1.4.1               VariantAnnotation_1.44.0
## [135] digest_0.6.30               graph_1.76.0
## [137] Biostrings_2.66.0           rmarkdown_2.18
## [139] htmlTable_2.4.1             restfulr_0.0.15
## [141] curl_4.3.3                  ggplot.multistats_1.0.0
## [143] Rsamtools_2.14.0            rjson_0.2.21
## [145] lifecycle_1.0.3             carData_3.0-5
## [147] BSgenome_1.66.2             fansi_1.0.3
## [149] pillar_1.9.0                lattice_0.20-45
## [151] GGally_2.1.2                KEGGREST_1.38.0
## [153] fastmap_1.1.0               httr_1.4.4
## [155] DEoptimR_1.0-11             survival_3.4-0
## [157] xts_0.13.0                  glue_1.6.2
## [159] png_0.1-7                   iterators_1.0.14
## [161] bit_4.0.5                   class_7.3-20
## [163] stringi_1.7.8               blob_1.2.3
## [165] RcppHNSW_0.4.1              latticeExtra_0.6-30
## [167] memoise_2.0.1               dplyr_1.0.10
## [169] irlba_2.3.5.1               e1071_1.7-13
## [171] future.apply_1.10.0
```
