# Supplementary material for: vizAPA: visualizing dynamics of alternative polyadenylation from bulk and single-cell data
Source: Bioinformatics. 2024 Mar 14;40(3):btae099. doi: 10.1093/bioinformatics/btae099 (PMC10950478; doi:10.1093/bioinformatics/btae099)
Supplement: btae099_Supplementary_Data [file btae099_supplementary_data.zip › vizAPA_user_mannual_1_A_minimal_tutorial.pdf]

Using vizAPA: a minimal tutorial

Xiaohui Wu, Xingyu Bi, Wenbin Ye

2024-01-09

Contents

Overview **1**

Demo PACdataset **2**

`vizStats` to explore APA dynamics across cell categories **4**

`vizStats` to summarize APA usages across cell categories 4

`vizStats` to summarize APA expression levels across cell categories 13

`vizUMAP` to plot 2D-embeddings **17**

`vizAPAmarkers` to visualize APA markers across cell categories **22**

`getAPAmarkers` to get APA markers 22

`vizAPAMarkers` to visualize multiple APA markers 24

Visualize single APA marker 30

APA markers for one cell type 33

`vizTracks` to plot gene model, pAs and BAM tracks **34**

Prepare BAM files 35

Load genome annotation to an annoHub 35

Plot tracks for a specified gene 35

Session information **39**

Overview

This tutorial takes a `PACdataset` object storing a list of poly(A) sites as input and describes some simple but commonly used functions of vizAPA.

Demo PACdataset

In the package of vizAPA, there is a demo `PACdataset` object of mouse sperm cells, containing 974 pAs [poly(A) sites] from 413 genes. This PACdataset has been annotated, with both pAs' and cells' meta data. The data contains three differentiation stages, including early stage (spermato-cytes, SC), intermediate stage (round spermatids, RS), and late stage (elongating spermatids, ES).

```
library(vizAPA)

data(scPACds, package='vizAPA')

# summary of the PACdataset
movAPA::summary(scPACds)
```

```
## PAC# 974
## sample# 955
## summary of expression level of each PA
##    Min. 1st Qu.  Median    Mean 3rd Qu.    Max.
##       1      72     957    3151    3636   96363
## summary of expressed sample# of each PA
##    Min. 1st Qu.  Median    Mean 3rd Qu.    Max.
##    1.00   64.25  452.50  452.05  810.00  955.00
## gene# 413
##      nPAC
## 3UTR  974
```

```
# cell meta data
head(scPACds@colData)
```

```
##                  orig.ident nCount_RNA nFeature_RNA RNA_snn_res.0.5
## AAACCTGAGCTTATCG       gene      23617         5061               9
## AAACCTGGTTGAGTTC       gene      19555         4802               9
## AAACCTGTCAACGAAA       gene      23467         5009               8
## AAACGGGCACAGGTTT       gene      28832         5484               8
## AAACGGGTCATTTGGG       gene      18931         4819               8
## AAACGGGTCCTCATTA       gene      15734         3855               8
##                  seurat_clusters celltype       UMAP_1      UMAP_2
## AAACCTGAGCTTATCG               9       RS  0.361751856 4.528803031
## AAACCTGGTTGAGTTC               9       RS -0.119255482 4.563224952
## AAACCTGTCAACGAAA               8       RS  3.023034156 4.074635188
## AAACGGGCACAGGTTT               8       RS  3.322863163  3.81046788
## AAACGGGTCATTTGGG               8       ES   4.73071772 3.419416826
## AAACGGGTCCTCATTA               8       ES  5.306060375 3.274766843
##                           barcode
## AAACCTGAGCTTATCG AAACCTGAGCTTATCG
## AAACCTGGTTGAGTTC AAACCTGGTTGAGTTC
## AAACCTGTCAACGAAA AAACCTGTCAACGAAA
## AAACGGGCACAGGTTT AAACGGGCACAGGTTT
## AAACGGGTCATTTGGG AAACGGGTCATTTGGG
## AAACGGGTCCTCATTA AAACGGGTCCTCATTA
```

The coordinate labels of the 2D-embedding have already been stored in the PACdataset. For this data, the labels are `UMAP_1` and `UMAP_2`. Otherwise, users can use `reduceDim()` to get the 2D-embeddings for the PACdataset.

```r
colnames(scPACds@colData)
```

```
## [1] "orig.ident"      "nCount_RNA"      "nFeature_RNA"    "RNA_snn_res.0.5"
## [5] "seurat_clusters" "celltype"        "UMAP_1"          "UMAP_2"
## [9] "barcode"
```

```r
head(scPACds@colData)
```

```
##                  orig.ident nCount_RNA nFeature_RNA RNA_snn_res.0.5
## AAACCTGAGCTTATCG       gene      23617         5061               9
## AAACCTGGTTGAGTTC       gene      19555         4802               9
## AAACCTGTCAACGAAA       gene      23467         5009               8
## AAACGGGCACAGGTTT       gene      28832         5484               8
## AAACGGGTCATTTGGG       gene      18931         4819               8
## AAACGGGTCCTCATTA       gene      15734         3855               8
##                  seurat_clusters celltype       UMAP_1      UMAP_2
## AAACCTGAGCTTATCG               9       RS  0.361751856 4.528803031
## AAACCTGGTTGAGTTC               9       RS -0.119255482 4.563224952
## AAACCTGTCAACGAAA               8       RS  3.023034156 4.074635188
## AAACGGGCACAGGTTT               8       RS  3.322863163  3.81046788
## AAACGGGTCATTTGGG               8       ES   4.73071772 3.419416826
## AAACGGGTCCTCATTA               8       ES  5.306060375 3.274766843
##                           barcode
## AAACCTGAGCTTATCG AAACCTGAGCTTATCG
## AAACCTGGTTGAGTTC AAACCTGGTTGAGTTC
## AAACCTGTCAACGAAA AAACCTGTCAACGAAA
## AAACGGGCACAGGTTT AAACGGGCACAGGTTT
## AAACGGGTCATTTGGG AAACGGGTCATTTGGG
## AAACGGGTCCTCATTA AAACGGGTCCTCATTA
```

Since the dataset already contains cell coordinates of UMAP, it is easy to view the UMAP plot of this dataset with vizAPA. Here the `Annotation` plot shows the cell type annotation and the `MEAN` plot shows the mean read counts of all pAs in this PACdataset. For more details on the usage of vizUMAP, please refer to the following chapters.

```r
vizUMAP(scPACds, group='celltype', xcol='UMAP_1', ycol='UMAP_2')
```

```
## vizUMAP: group=celltype, x=UMAP_1, y=UMAP_2
```

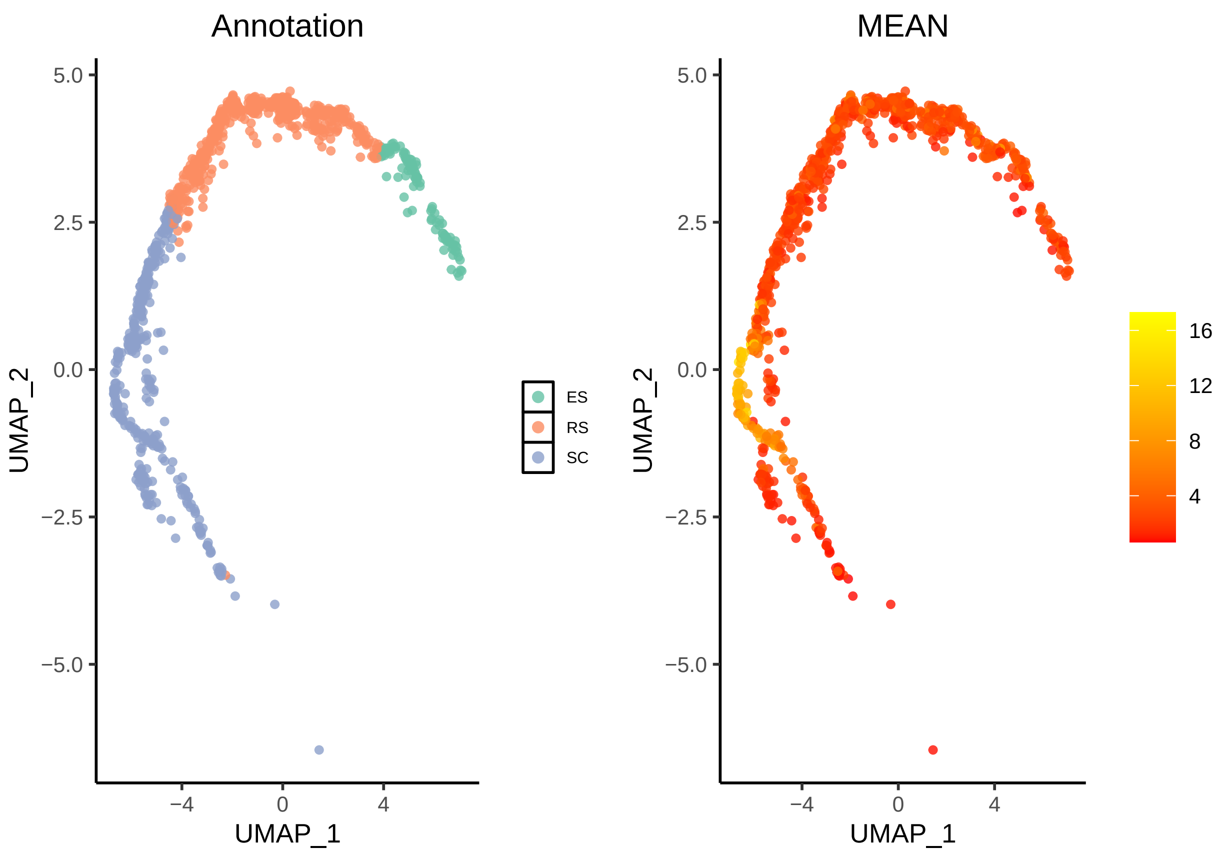

We can use `eoffice::topptx` to export the image in editable PPT format.

```
eoffice::topptx(filename = 'figures.pptx', title="vizUMAP_annotation_RUD",
                width = 8, height = 4, append=FALSE)
```

`vizStats` to explore APA dynamics across cell categories

In addition to `vizUMAP`, `vizStats` draws different types of plots, including boxplot, violin plot, dot plot, bubble plot, and heatmap to show expression (pA read counts or APA ratio) of given pAs or pAs in a gene across different conditions (e.g., cell types).

`vizStats` to summarize APA usages across cell categories

To investigate the global APA dynamics at the single-cell level, we calculated APA usage for each gene represented by RUD (Relative Usage of Distal poly(A) site) score of each cell. A larger RUD value of a gene in a cell means the longer 3'UTR of the gene in the cell.

Note: `getAPAindexPACds` only implements the RUD index in `movAPA`, users can use `movAPA::movAPAindex` for more types of APA index.

```
# change the level order of the factor celltype in scPACds
# to make it consistent with sperm differentiation
scPACds@colData$celltype=factor(scPACds@colData$celltype,
                                levels=c('SC','RS','ES'))
```

```
# get 3UTR PACds, only genes with 3'UTR APA can be used for RUD calculation.
# this scPACds has only 3UTR pAs, so this step will remove nothing
scPACds<-scPACds[scPACds@anno$ftr=="3UTR"]
# get RUD
RUD=getAPAindexPACds(scPACds, choose2PA = "PD")
```

First, we plot a violin plot with dots to show the mean RUD of all genes in the PACdataset, which reflects the global 3'UTR changes during the three stages. The plot shows transition of 3' UTR shortening (i.e., decreased RUD scores) during sperm cell differentiation (SC -> RS -> ES).

```
vizStats(RUD, group='celltype', figType="dot")
```

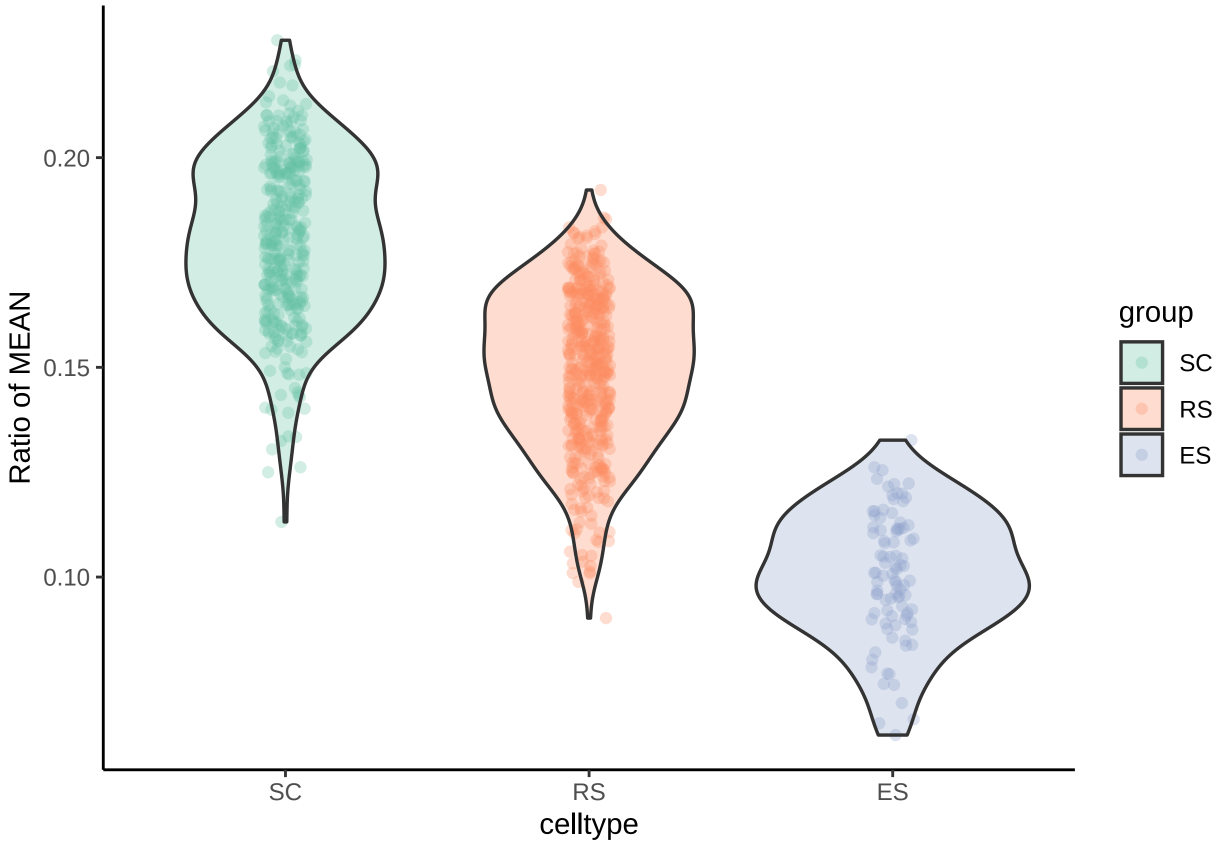

We can also plot a specific gene or pA using `vizStats`. First, we check the gene column in the `anno` slot of scPACds, and found that the gene is represented as entrez id. It is easy to use `getAnnoGenes` in vizAPA to get genomic ranges of all genes from different genome annotation sources. Here we replaced the original entrez id to gene symbol for the RUD object.

```
head(RUD@anno)
```

```
##                gene
## 100040531 100040531
## 100041352 100041352
## 100041639 100041639
## 100042055 100042055
```

```
## 100101807 100101807
## 100165       100165
```

```
library(Mus.musculus, quietly = TRUE)
orgdb=Mus.musculus
genes=getAnnoGenes(orgdb)
RUD@anno=merge(RUD@anno, genes, by.x='gene', by.y='gene_entrezid', all.x=TRUE)
# there are two entrez ids not in orgdb, so we use entrez id instead symbol
RUD@anno$gene_symbol[is.na(RUD@anno$gene_symbol)]=
  RUD@anno$gene[is.na(RUD@anno$gene_symbol)]
RUD@anno$entrezid=RUD@anno$gene
# set the gene column as gene_symbol
RUD@anno$gene=RUD@anno$gene_symbol
# set rownames of counts slot as gene_symbol
rownames(RUD@counts)=RUD@anno$gene_symbol
# after conversion
head(RUD@anno)
```

```
##        gene   chr strand     start       end       gene_ensembl gene_symbol
## 1 100040531  <NA>   <NA>        NA        NA               <NA>   100040531
## 2    Tcp10c chr17      +  13354572  13377223 ENSMUSG00000052469      Tcp10c
## 3  Dynlt2a2 chr17      -  14964242  15041537 ENSMUSG00000079710    Dynlt2a2
## 4   Gm10377 chr14      -  41767172  43015628 ENSMUSG00000095226     Gm10377
## 5  Fam177a2 chr12      +  55124469  55217168 ENSMUSG00000094103    Fam177a2
## 6  AI507597  chr4      + 141614026 141615604 ENSMUSG00000073731    AI507597
##    entrezid
## 1 100040531
## 2 100041352
## 3 100041639
## 4 100042055
## 5 100101807
## 6    100165
```

Here we chose an example gene ASRGL1 (66514, ENSMUSG00000024654) for demonstration, showing the RUD score of this gene in each cell type.

```
gene='Asrgl1'
RUD@anno[RUD@anno$gene==gene, ]
```

```
##       gene   chr strand   start     end       gene_ensembl gene_symbol entrezid
## 266 Asrgl1 chr19      - 9109868 9135636 ENSMUSG00000024654      Asrgl1    66514
```

Here is an example to plot a heatmap to show the RUD score of pAs in a given gene across cell types. The RUD value of this gene across cells in each cell type are averaged.

```
## plot a heatmap for summarizing average expression of pAs in this gene
vizStats(RUD, group='celltype', gene=gene, figType="heatmap")

## we can change the text size or remove text in the heatmap (heat.text.size=0)
## or change the number of digits for the averaged value
vizStats(RUD, group='celltype', gene=gene, figType="heatmap",
         statTheme = list(heat.text.size=6, heat.text.digits=4))
```

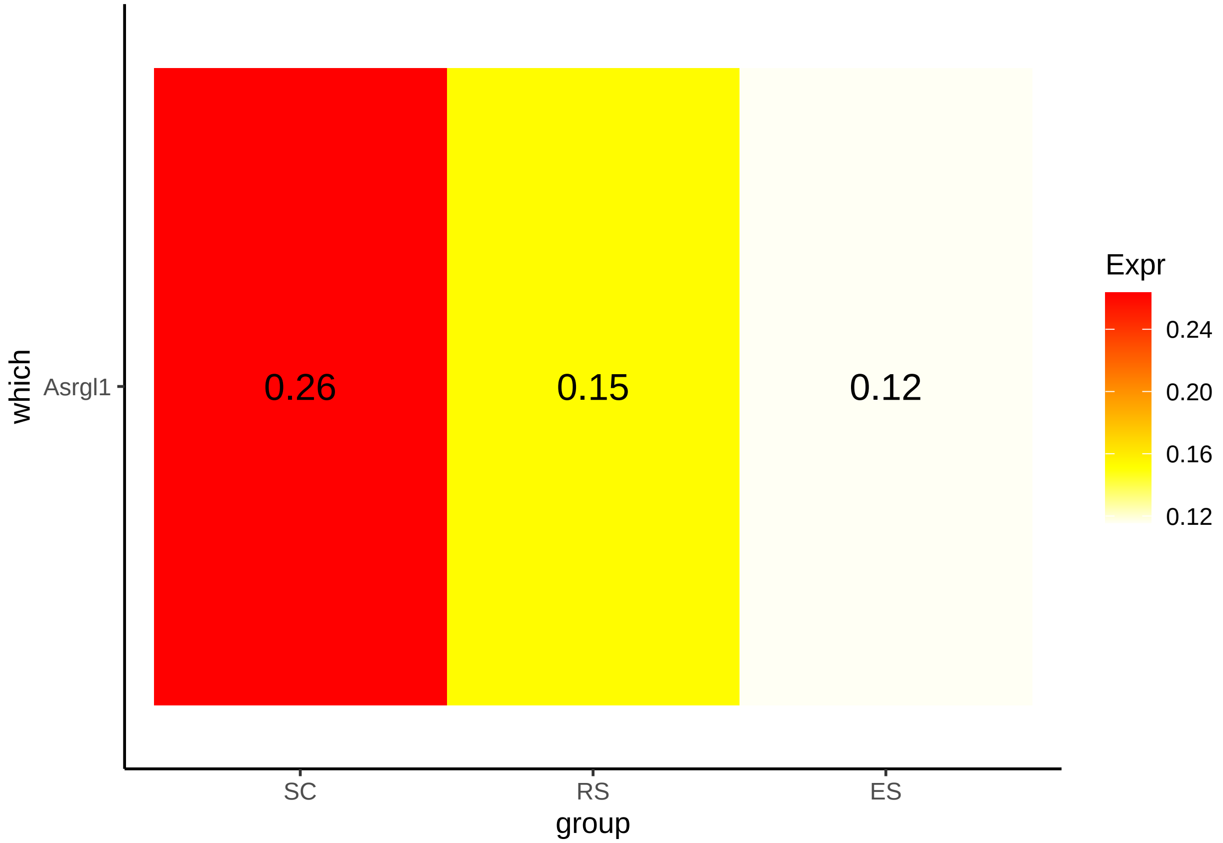

which
Asrgl1
0.26
0.15
0.12
SC
RS
ES
group
Expr
0.24
0.20
0.16
0.12

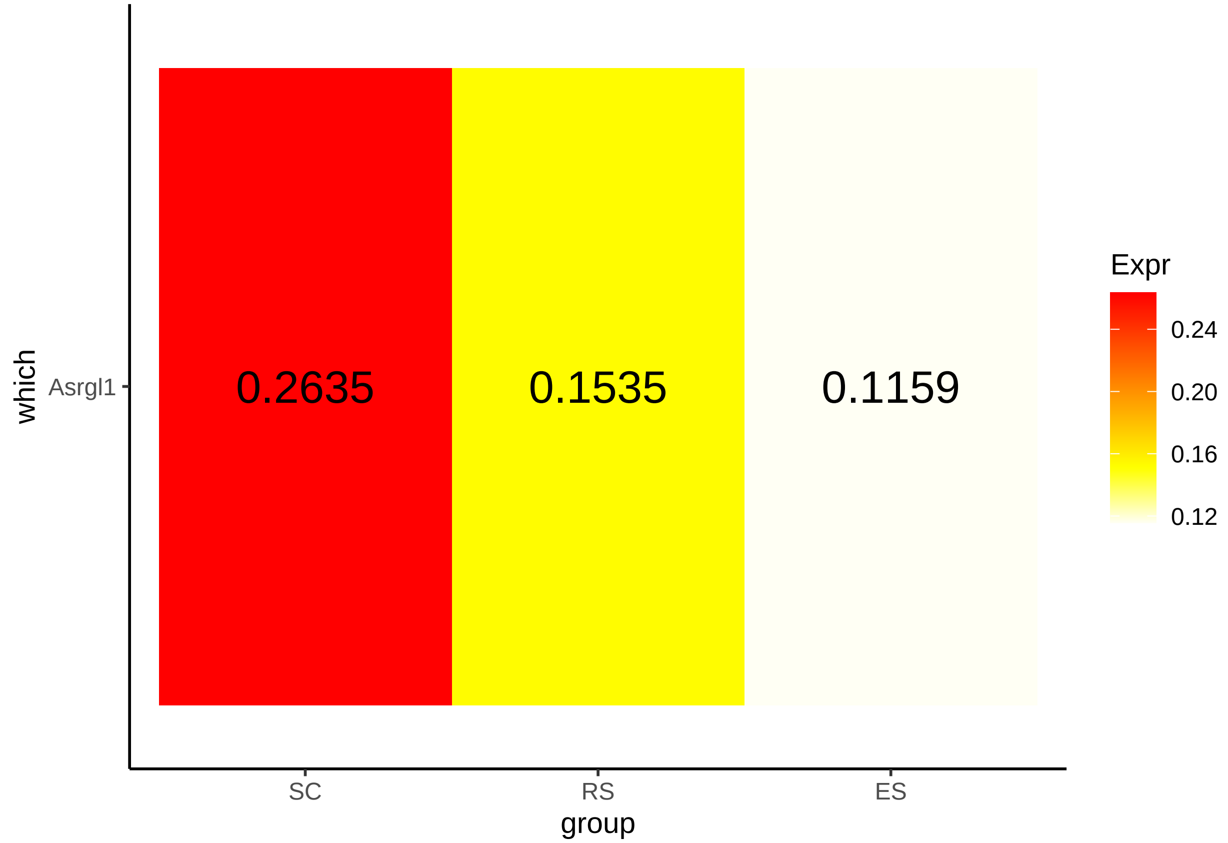

The returned plot is a ggplot2 object, we can using ggplot2 code to change the plot. For example, here we change the color of the heatmap.

```
p=vizStats(RUD, group='celltype', gene=gene, figType="heatmap")
p+ggplot2::scale_fill_gradientn(colours = grDevices::heat.colors(50)) +
  ggplot2::theme_minimal()
```

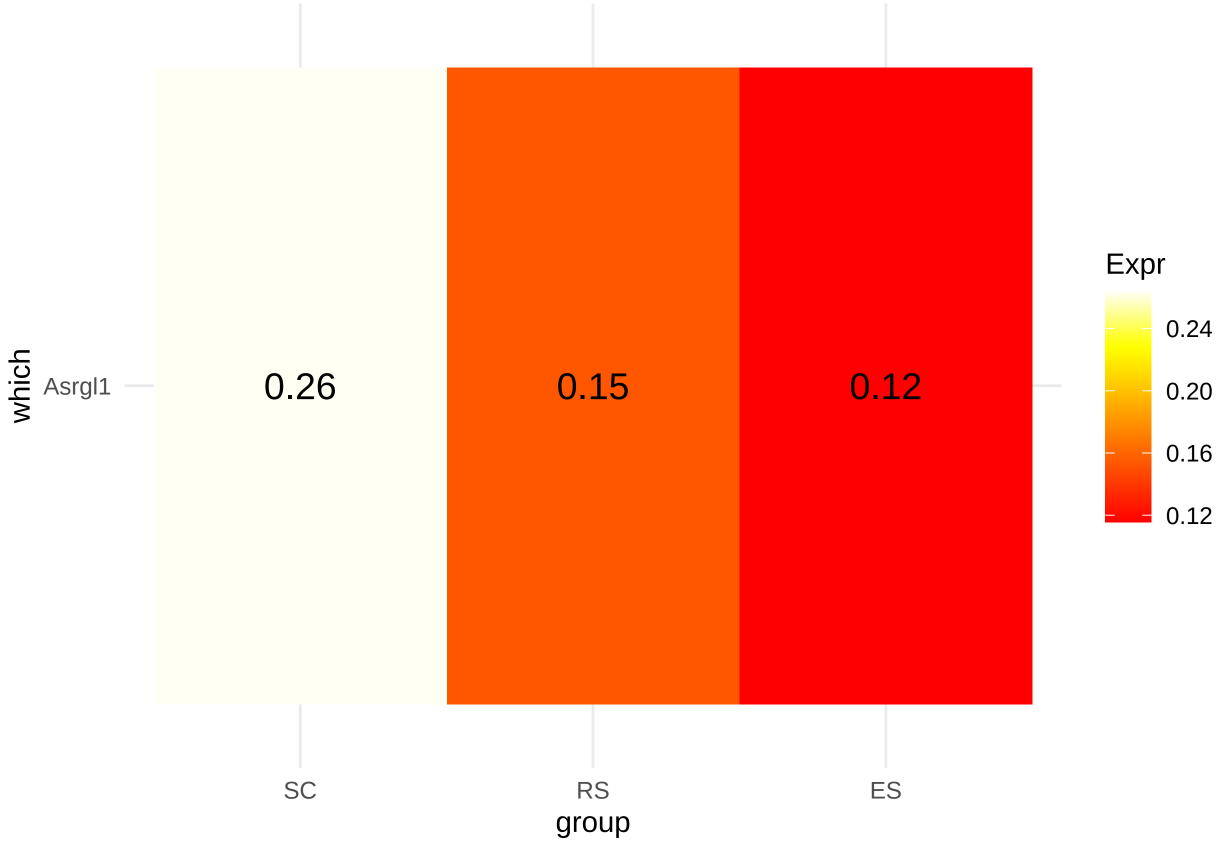

We can also show a boxplot instead. In this case, RUD scores of the gene in cells of the same cell type are not averaged as in the heatmap, so the boxplot shows the RUD profile of each gene in each cell type.

```
vizStats(RUD, group='celltype', gene=gene, figType="box")
```

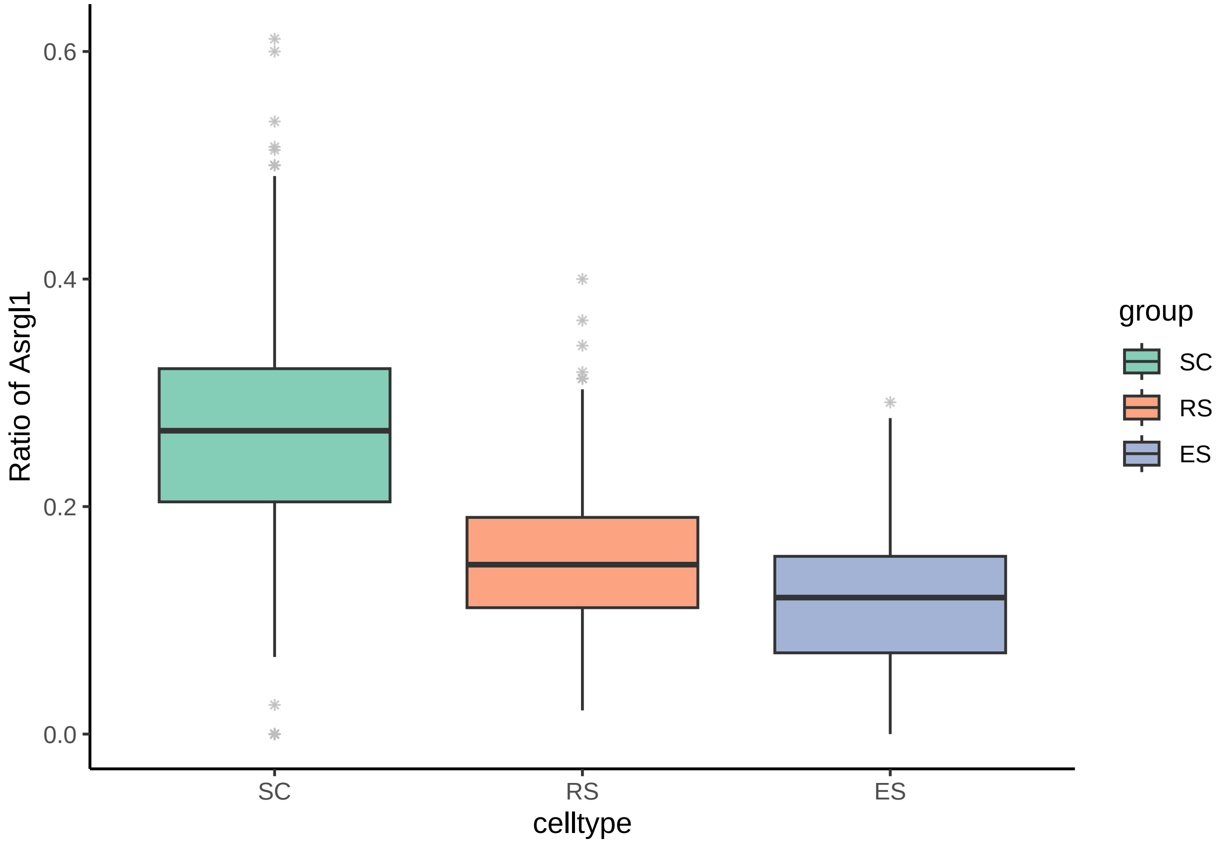

In addition, we can plot other types of plots, including violin plot, dot plot, and bubble plot. The violin plot and dot plot are similar to the boxplot.

```
# violin plot
vizStats(RUD, group='celltype', gene=gene, figType="violin")
```

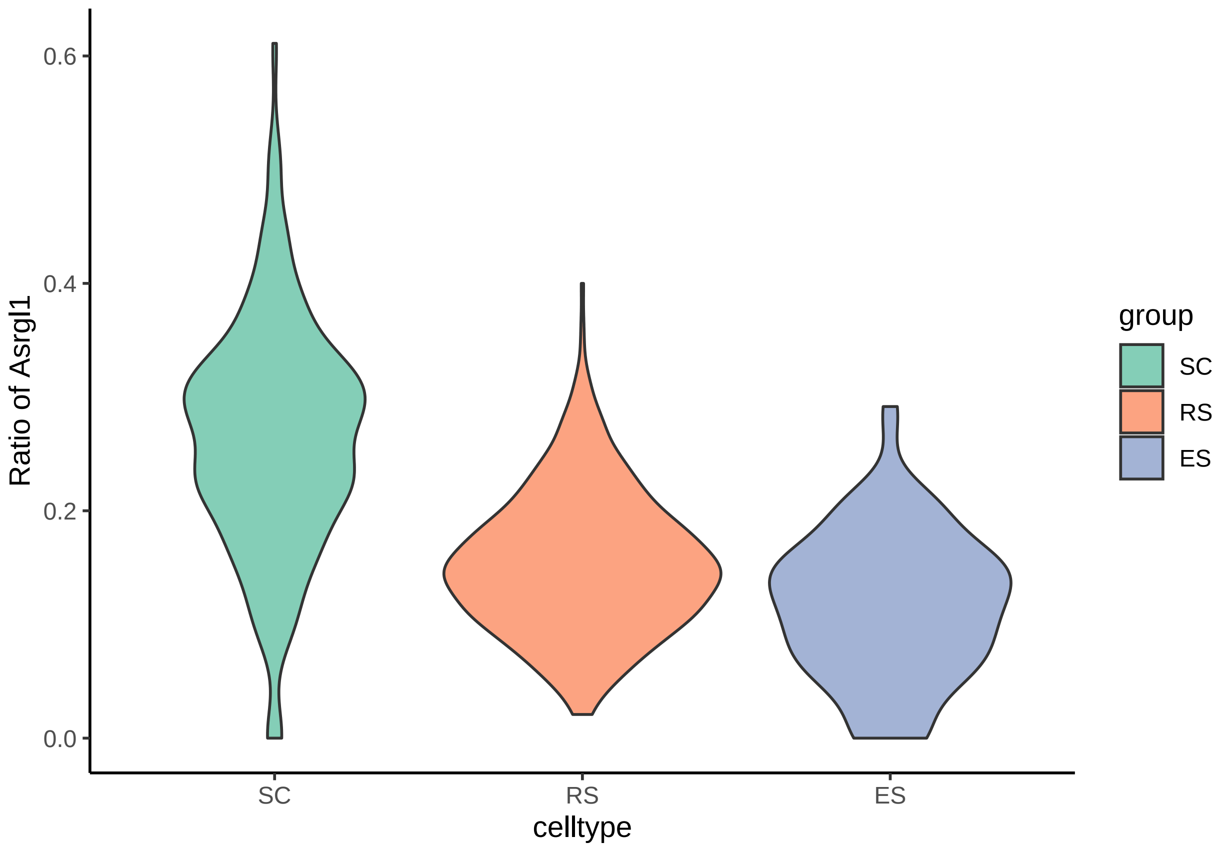


```
# violin plot with dots, and each dot is one cell
vizStats(RUD, group='celltype', gene=gene, figType="dot")
```

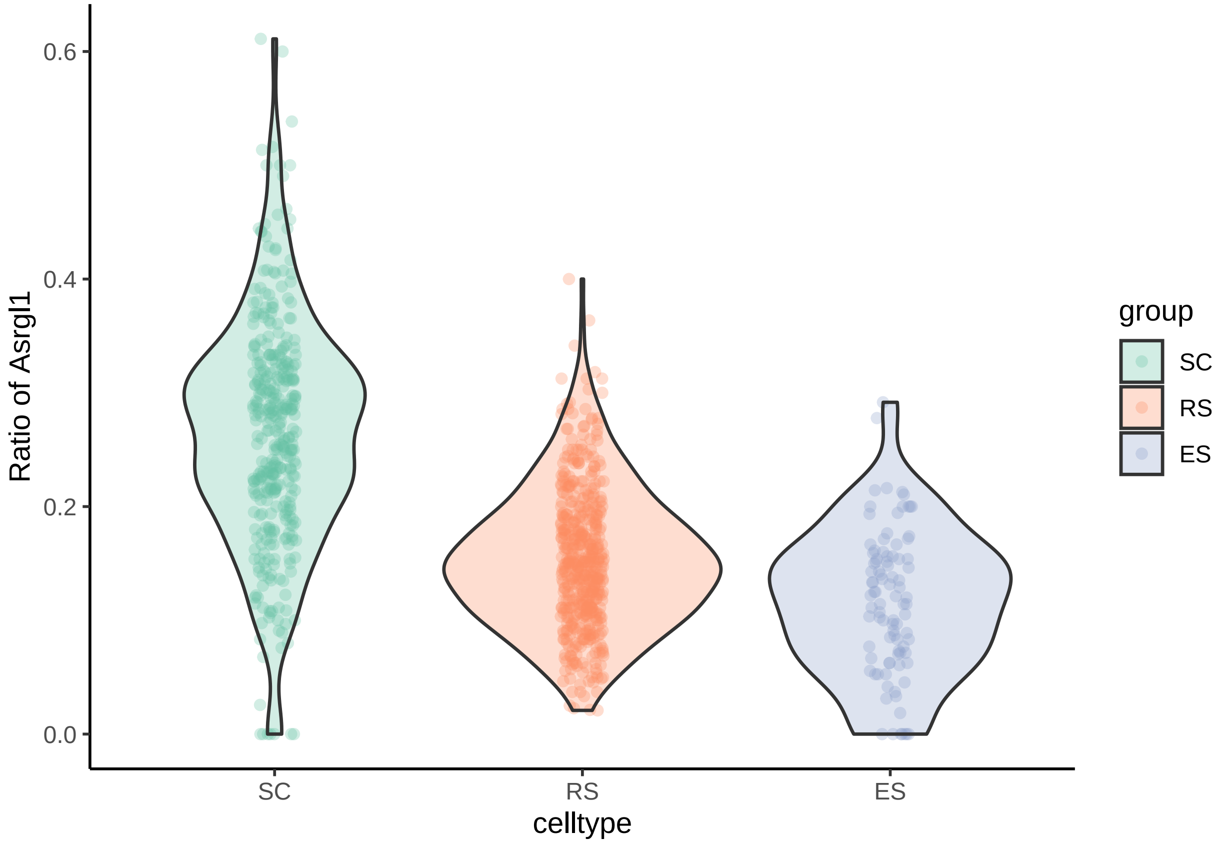


```
#eoffice::topptx(filename = 'figures.pptx', title="gene_dot_plot",
#                 width = 4, height = 4, append=TRUE)
```

The bubble plot is similar to the heatmap, but it also displays the number of cells where each pA expressed (RUD>0). The larger the bubble is, the higher number of cells with RUD>0 of that pA.

```
vizStats(RUD, group='celltype', gene=gene, figType="bubble")
```

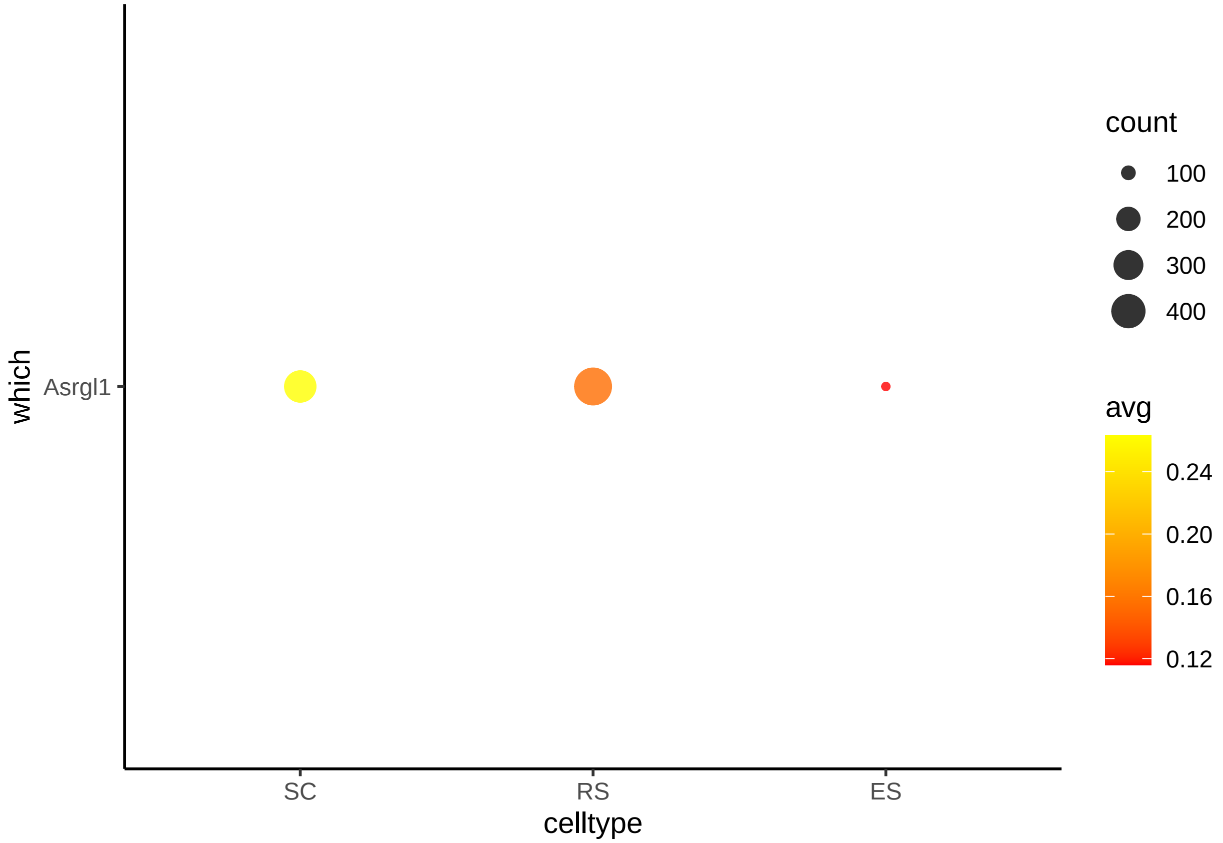

`vizStats` to summarize APA expression levels across cell categories

In addition to using RUD score of a gene, we can also plot expression levels of individual pAs in a gene. Here is an example to plot a heatmap to show the **expression levels** of pAs in a given gene across cell types. The expression levels (read counts) of each pA of this gene across cells in each cell type are averaged.

```
# for scPACds, the id type is entrezid
geneid=66514
## plot a heatmap for summarizing average expression of pAs in this gene
vizStats(scPACds, group='celltype', gene=geneid, figType="heatmap")
```

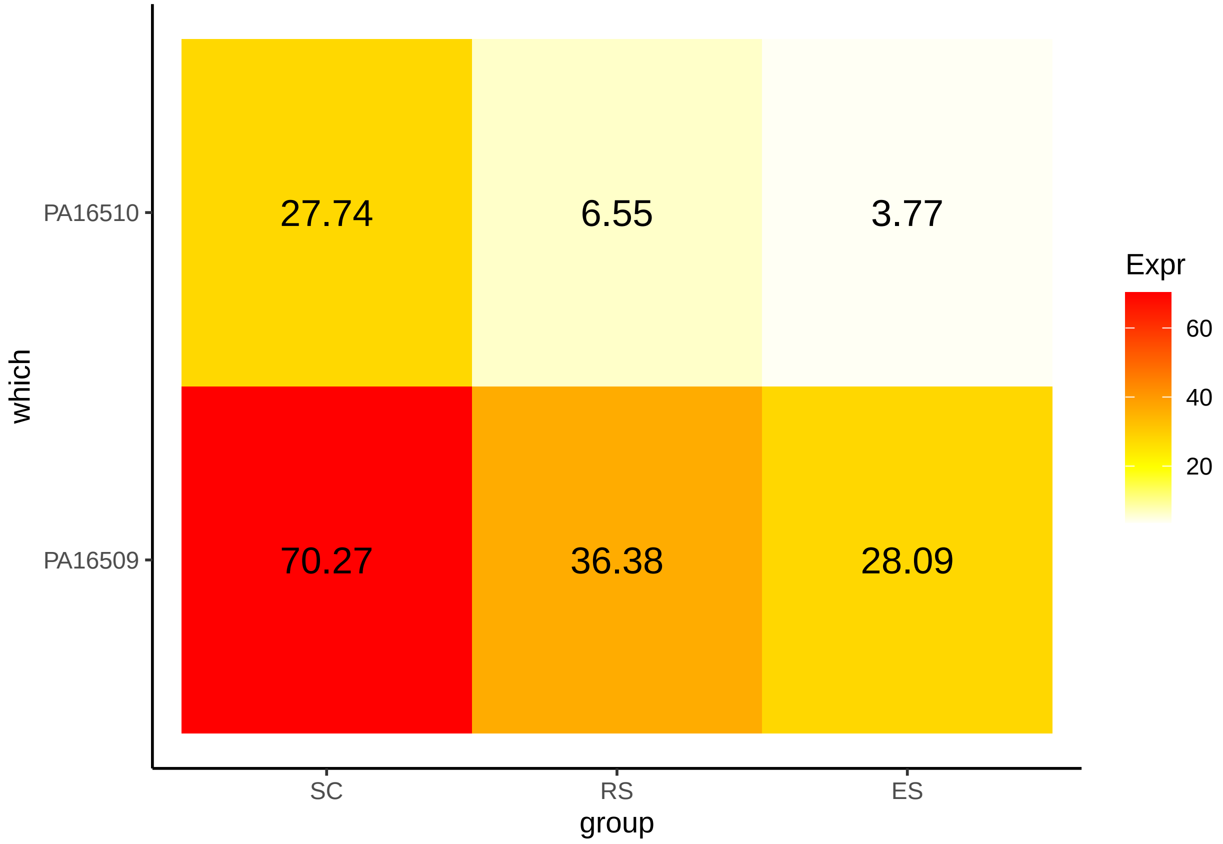

We can also show a boxplot instead. In this case, expression levels of pA in cells of the same cell type are not averaged as in the heatmap, so the plot shows the expression profile of each pA in each cell type.

```
# plot a boxplot to compare the pA usage of this gene in different cell types
vizStats(scPACds, group='celltype', gene=geneid, figType="box")

#eoffice::topptx(filename = 'figures.pptx', title="gene_box_plot",
#                width = 4, height = 4, append=TRUE)
```

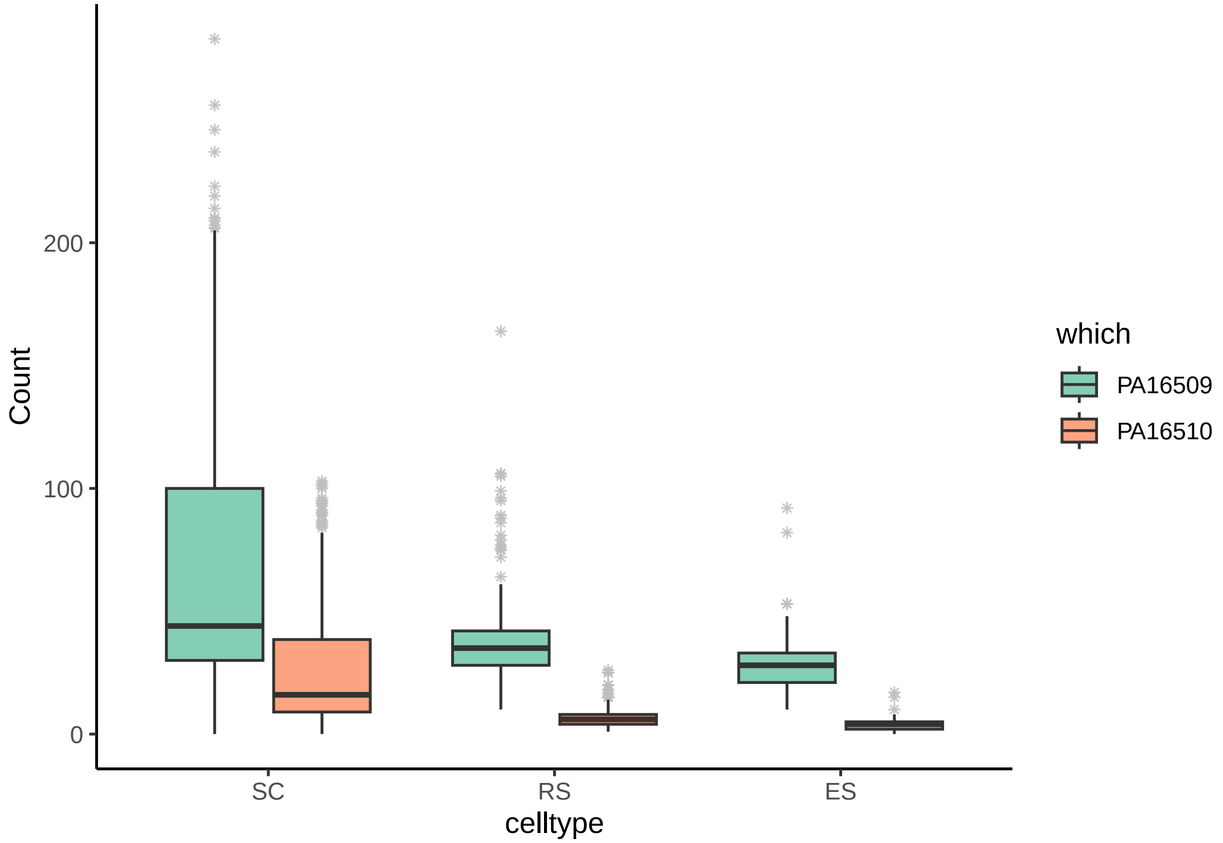

The bubble plot is similar to the heatmap, but it also displays the number of cells where each pA expressed (expression level>0). The larger the bubble is, the higher number of cells with >0 count of that pA.

```
vizStats(scPACds, group='celltype', gene=geneid,  figType="bubble")
```

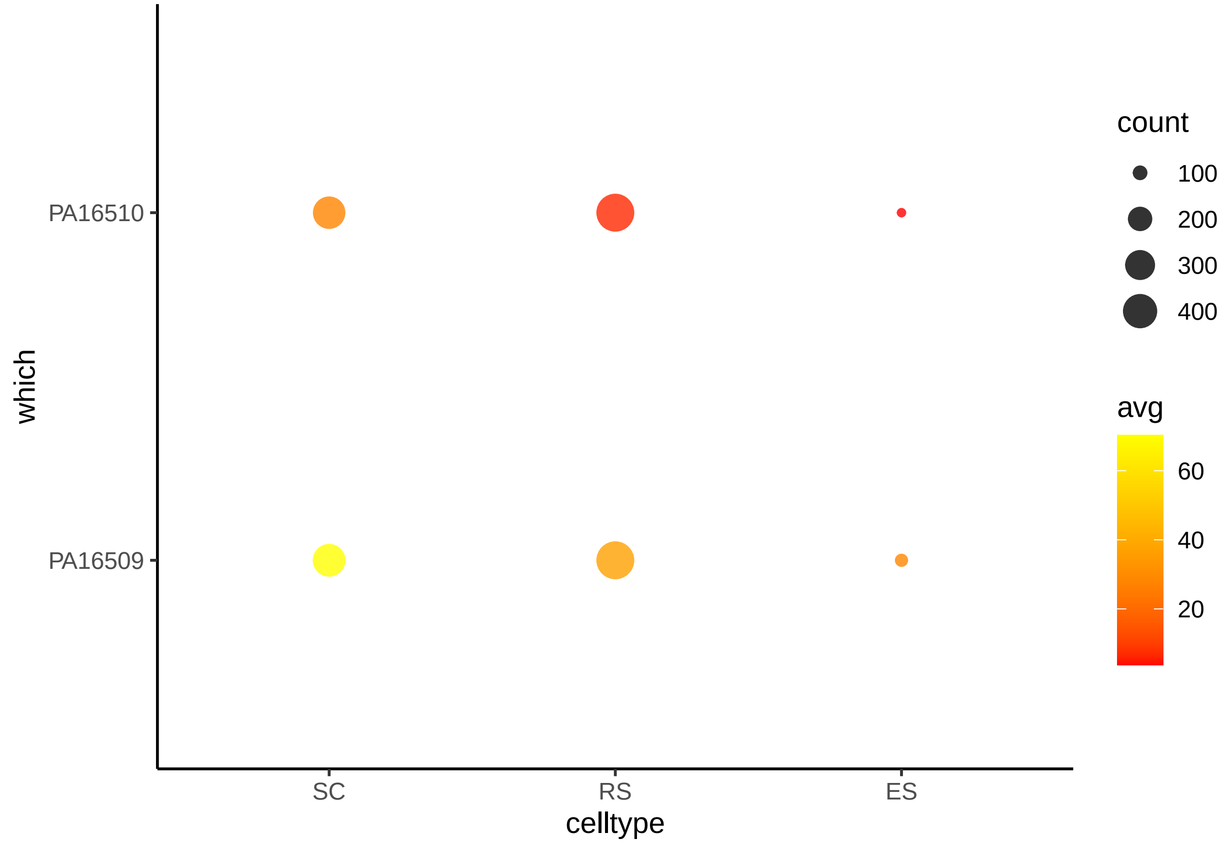

If no pA or gene is provided, then it is to plot the mean of all pAs (if it is a pA matrix) or genes (if it is a gene or APA index matrix) in the PACdataset. Here the scPACds is a pA-expression matrix, so `vizStats` plots the mean value of all pAs across cell types.

```
vizStats(scPACds, group='celltype', figType="dot")
```

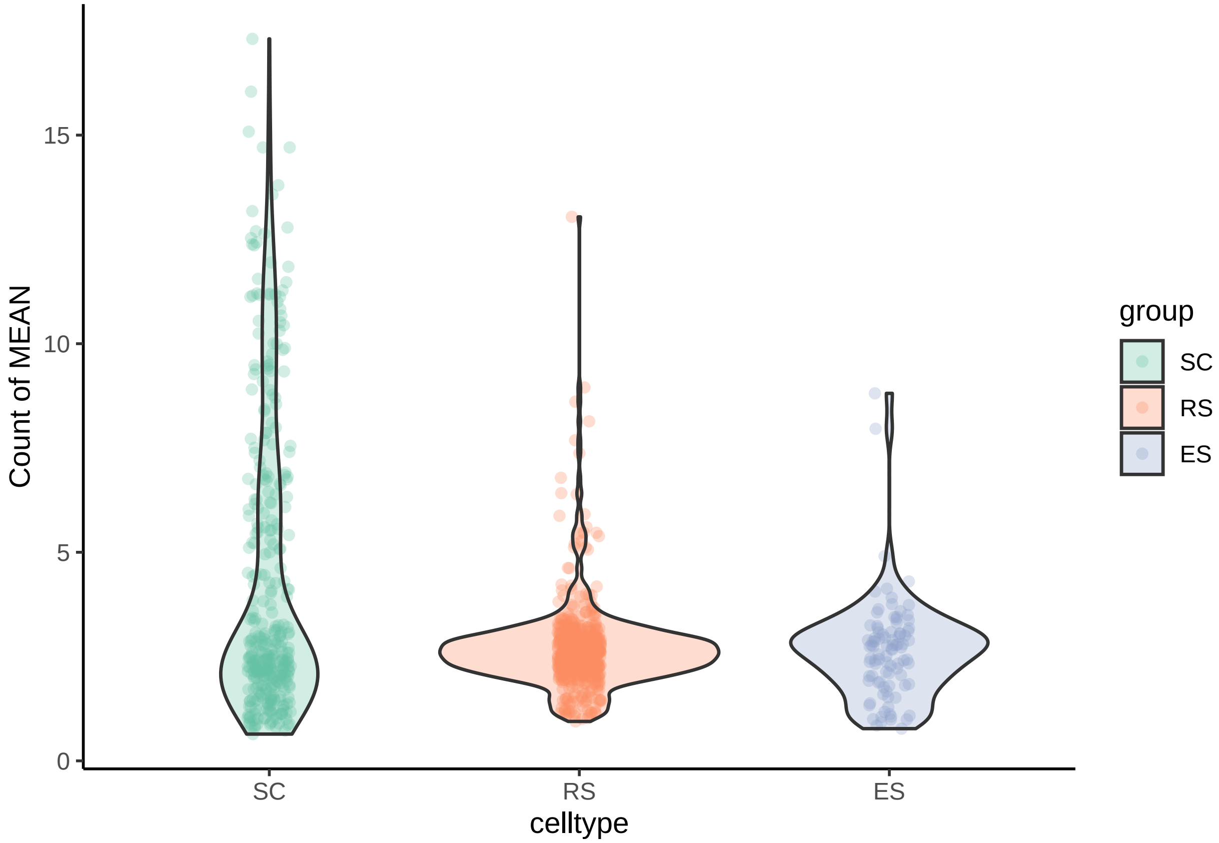

vizUMAP to plot 2D-embeddings

`vizUMAP` plots a UMAP plot where each point is a cell and it's positioned based on the cell embedding determined by the reduction technique.

To investigate the global APA dynamics at the single-cell level, we used `vizUMAP` to overlay the mean APA usage represented by RUD score of each cell on the 2D-embeddings. Here, the plot with gradient colors shows gradual transition of 3' UTR shortening (i.e., lower RUD scores) during sperm cell differentiation (SC > RS > ES).

```
# show UMAP using RUD scores
vizUMAP(RUD, group='celltype', xcol='UMAP_1', ycol='UMAP_2')
```

```
## vizUMAP: group=celltype, x=UMAP_1, y=UMAP_2
```

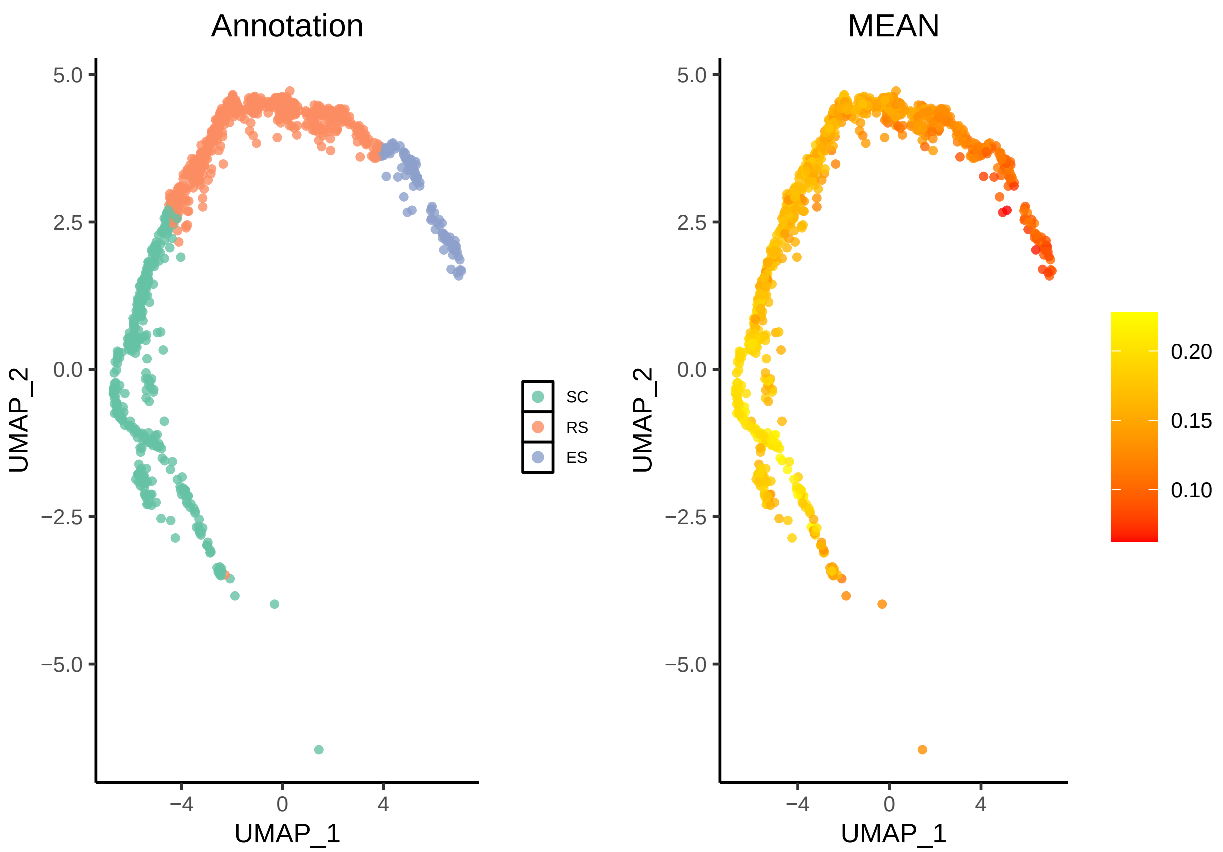


```
#eoffice::topptx(filename = 'figures.pptx', title="vizUMAP_annotation_RUD",
#                width = 8, height = 4, append=TRUE)
```

In addition to plot all genes or pAs in a PACdataset, providing a gene id or a list of genes in the gene column of the PACdataset, we can plot a UMAP overlaying with the mean expression value or APA ratio (e.g., RUD score) of the gene(s).

Here we plot a UMAP for the Asrgl1 gene, overlaying the RUD scores of this gene in each cell.

```
vizUMAP(RUD,
        group='celltype', xcol='UMAP_1',
        ycol='UMAP_2', genes=gene)
```

```
## vizUMAP: group=celltype, x=UMAP_1, y=UMAP_2
```

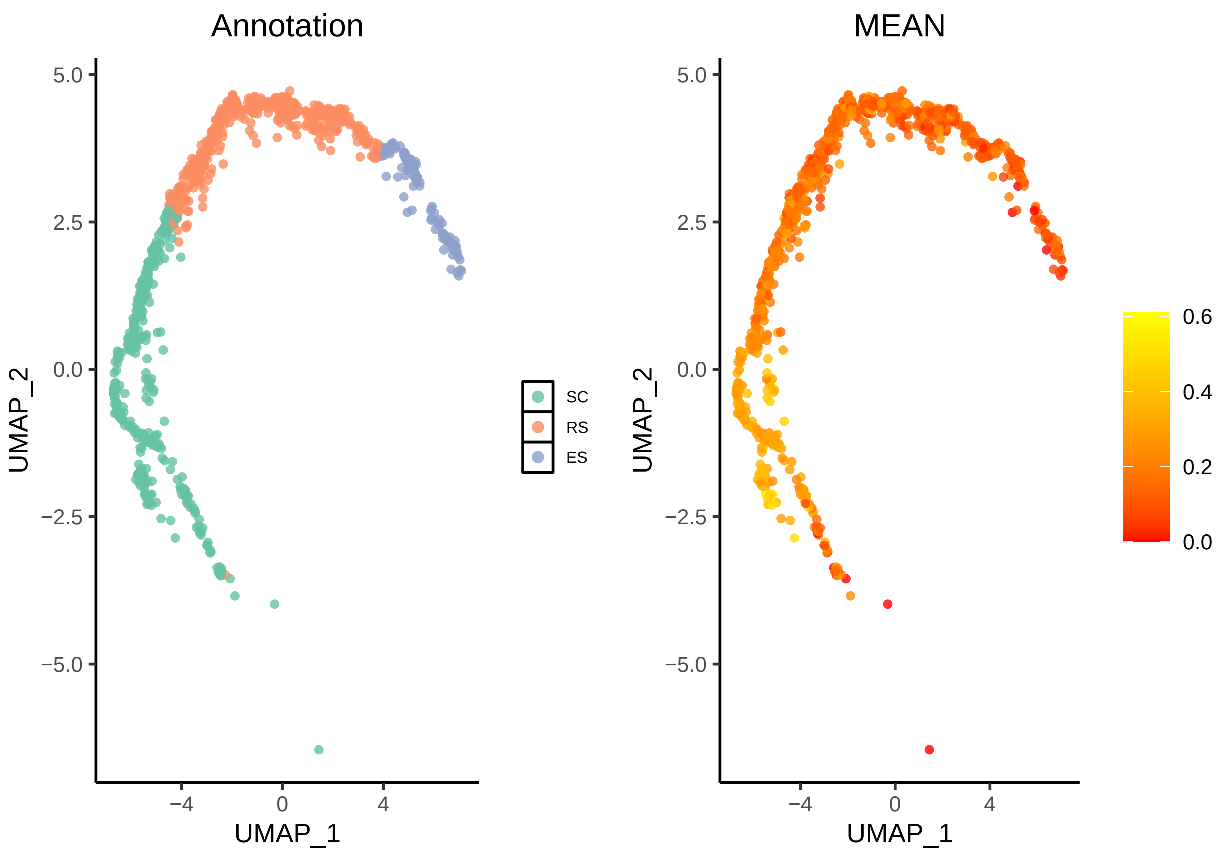

It seems that the default color gradient is not very distinguishing, we can modify the parameters of `statTHEME` to change colors for the UMAP. Please see the help document of `?setStatTheme` for details.

After changing color, tt is clearer that the usage of the distal poly(A) site denoted by RUD score is varied across stages. The RUD score of this gene is decreased from SC to ES, suggesting the APA dynamics of of this gene during mouse spermatogenesis. It is also observed that the color of cells in the same cell type is not consistent, suggesting heterogeneous APA isoform expression among individual cells.

```
vizUMAP(RUD,
        group='celltype', xcol='UMAP_1', ycol='UMAP_2',
        annoUMAP=TRUE,
        genes=gene,
        statTheme = list(scale.low.col='green', scale.high.col='red'))
```

```
## vizUMAP: group=celltype, x=UMAP_1, y=UMAP_2
```

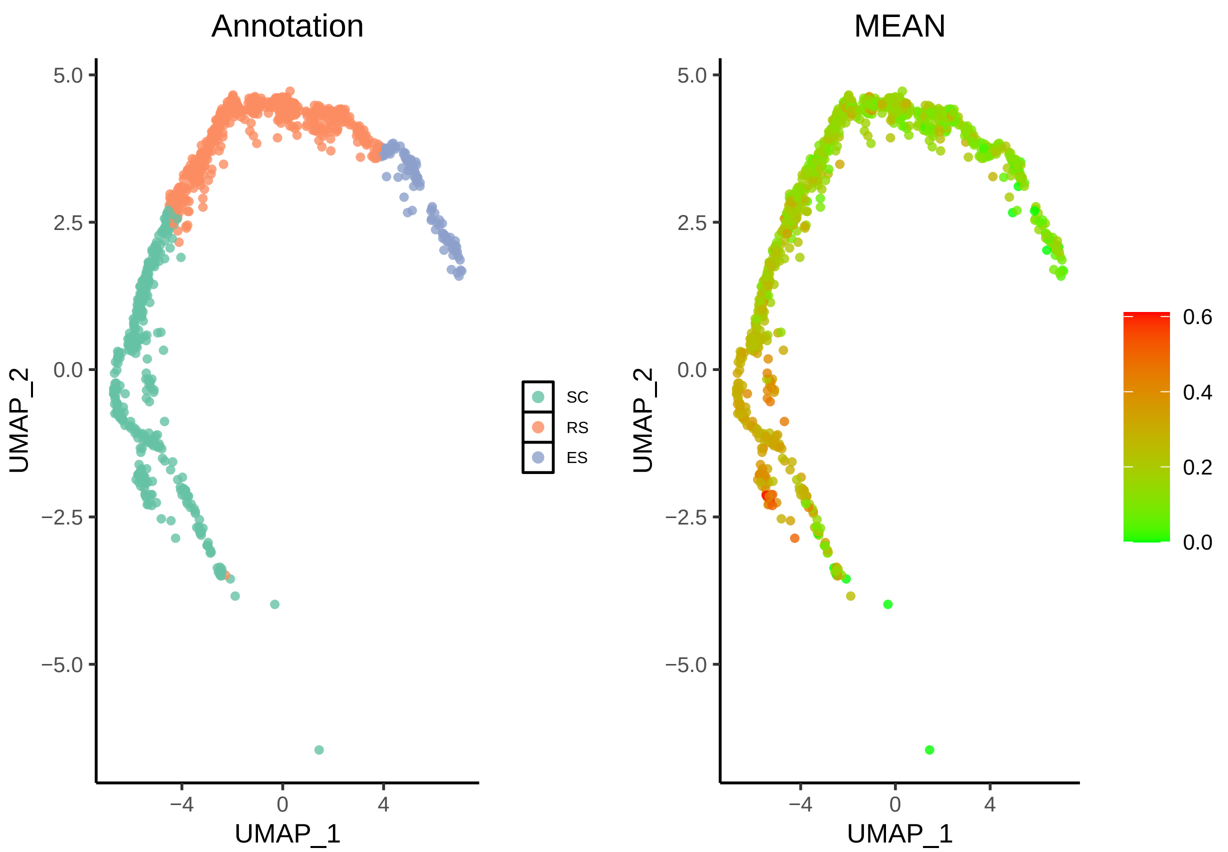

Further, we can use `vizStats` to show RUD distributions using other plots, e.g., dot plot. It is clear that the overall higher RUD score in the SC stage and the heterogeneous RUD scores in each cell type.

```
vizStats(RUD, group='celltype', figType="dot", gene=gene)
```

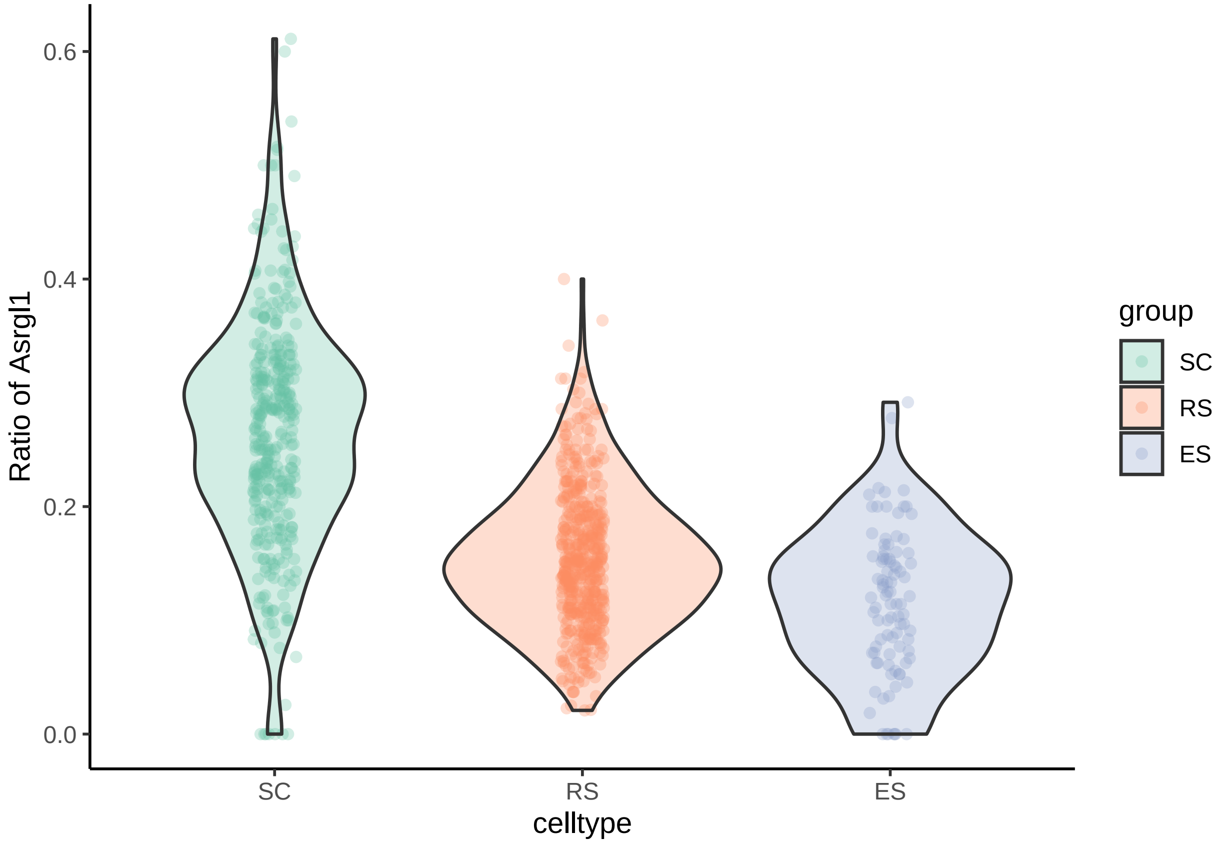

In addition to gene IDs or symbols, we can provide ids of pAs corresponding to the rownames in the PACdataset instead of genes. For example, here we only overlay one pA (PA16510) in the Asrgl1 gene.

```
PAids='PA16510'
vizUMAP(scPACds, group='celltype', xcol='UMAP_1', ycol='UMAP_2',
        PAs=PAids, annoUMAP = FALSE)
```

```
## vizUMAP: group=celltype, x=UMAP_1, y=UMAP_2
```

```
## $MEAN
```

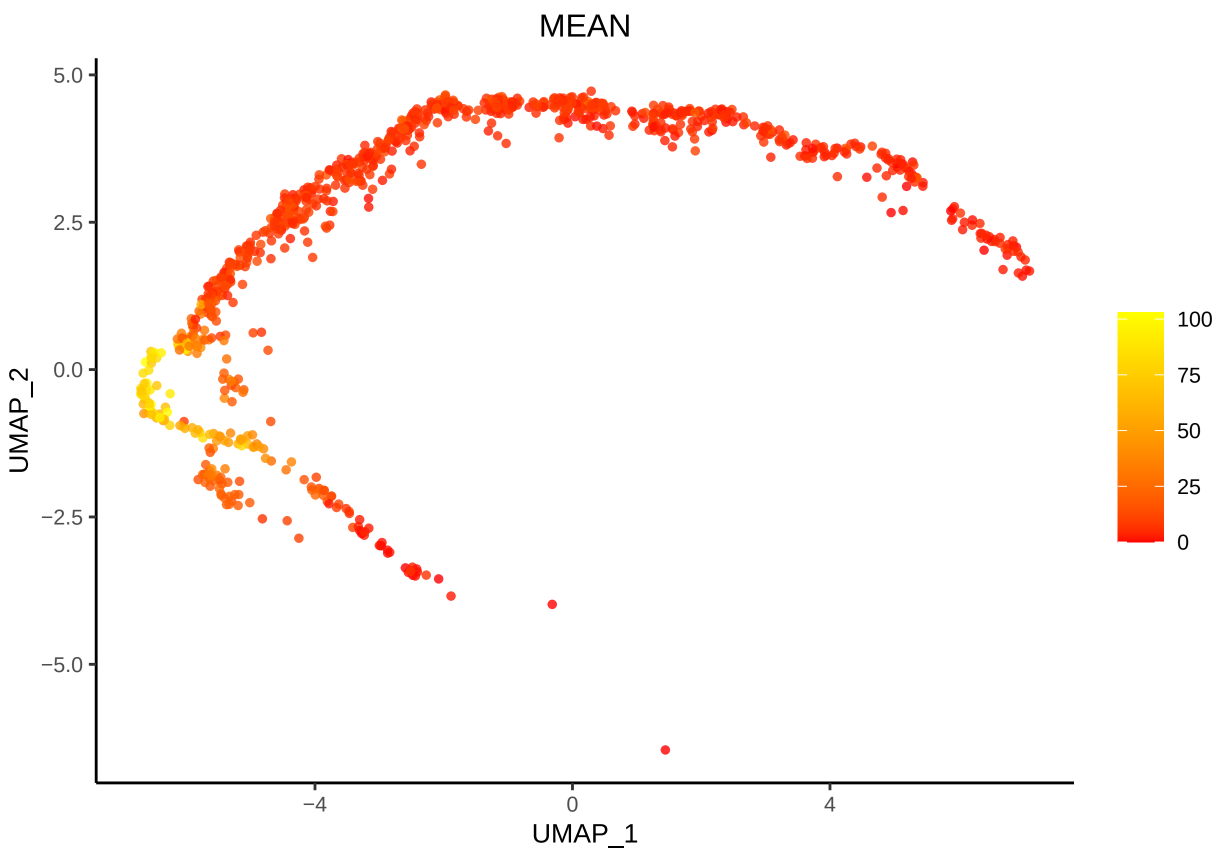

vizAPAmarkers to visualize APA markers across cell categories

getAPAmarkers to get APA markers

An APA marker is an APA gene with differential APA usage between two pAs in the 3'UTR of the gene. Leveraging `FindMarkers` used in `Seurat`, `getAPAmarkers` of vizAPA used different statistical tests (e.g., wilcoxon rank sum test) to test the significance of difference of RUD scores of a gene between two cell groups.

Of note, `getAPAmarkers` of vizAPA also allows using the read counts instead of RUD score to get APA markers, which treats each pA as a gene for differential expression (DE) detection.

```
m=getAPAmarkers(scPACds,  group='celltype', everyPair = TRUE)
```

However, normally we use APA index/ratio (e.g., RUD) to get APA markers. Here we obtain APA markers for each pair of cell types using the RUD object. The resulted table lists all APA markers between each two cell types. The column `avg_log2FC` stores the fold change of mean RUD score between cell types, with >0 means positive marker and <0 means negative marker.

```
## obtain APA markers by wilcox.test for each pair of cell types
m=getAPAmarkers(RUD,  group='celltype', everyPair = TRUE,
                min.pct = 0.1, logFC=0.12)
```

```
## PACds row = gene, PACds dataType = ratio
```

```
## It seems that PACds is APA ratio,
## will apply FindMarkers (default is wilcox-test) on the APA index
## to get DE APA events (each row is an APA gene).
```

```r
## show marker details
head(m)
```

```
##           p_val avg_log2FC pct.1 pct.2    p_val_adj cluster1 cluster2   rowid
## 64 9.196023e-70 -0.4378888 0.156 0.722 3.797958e-67       RS       SC    Slbp
## 65 2.086312e-68 -0.2711788 0.341 0.785 8.616468e-66       RS       SC   H3f3b
## 66 4.505883e-68 -0.3871289 0.449 0.931 1.860930e-65       RS       SC   Rdh11
## 67 2.155597e-64 -0.2836375 0.124 0.661 8.902618e-62       RS       SC   Pole3
## 68 2.282423e-64 -0.3367513 0.150 0.678 9.426408e-62       RS       SC Arl6ip6
## 69 3.128266e-63 -0.1314289 1.000 0.981 1.291974e-60       RS       SC  Asrgl1
##    direction
## 64  negative
## 65  negative
## 66  negative
## 67  negative
## 68  negative
## 69  negative
```

Next, we can count the number of APA markers. Apparently, there are most number of markers between ES and SC, with much higher of negative markers between them. The higher nagative markers between ES and SC means these markers have lower RUD scores (avg_log2FC<0, shorter 3'UTR) in ES than in SC. This is consistent with the fact that 3' UTR shortening is observed during sperm cell differentiation (SC > RS > ES).

```r
countAPAmarkers(m)
```

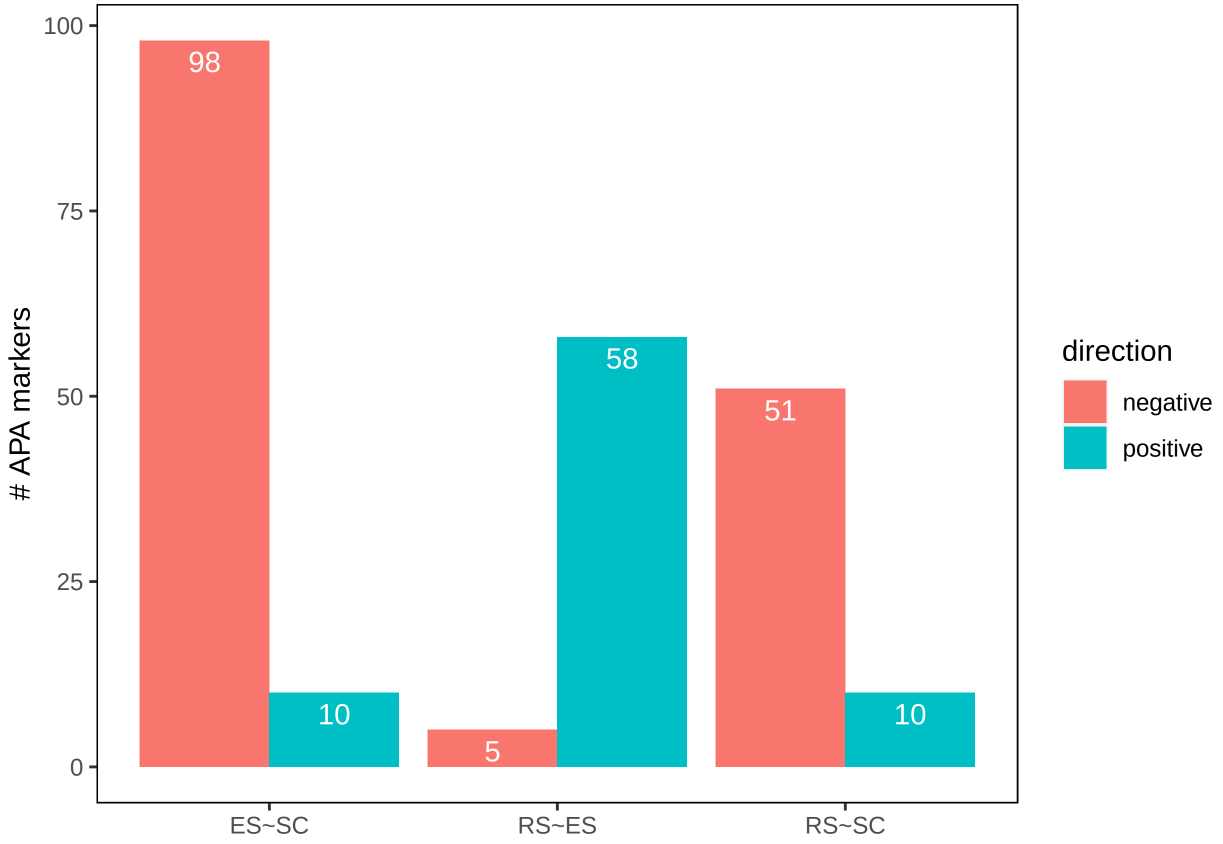


```
## # A tibble: 6 x 3
##   pair  direction Count
##   <chr> <chr>     <int>
## 1 ES~SC negative     98
## 2 ES~SC positive     10
## 3 RS~ES negative      5
## 4 RS~ES positive     58
## 5 RS~SC negative     51
## 6 RS~SC positive     10
```

```
#eoffice::topptx(filename = 'figures.pptx', title="marker_num",
#                width = 8, height = 4, append=TRUE)
```

`vizAPAMarkers` to visualize multiple APA markers

It is easy to visualize given APA markers by setting `markers` in `vizAPAMarkers`, with different plots including violin plot, heatmap, and bubble plot. For example, here we plot the top 6 markers. From the above markers' table, these markers are with differential RUD scores between RS and SC, and all these markers are with higher RUD scores in SC.

```
markers=m$rowid[1:6]
```

Here we visualize the top 6 APA markers in all the three cell types. In this plot, each marker corresponds to one row, with overall higher RUD scores in SC than in RS.

```r
vizAPAMarkers(RUD, group='celltype',
              markers=markers,
              figType = 'violin')
```

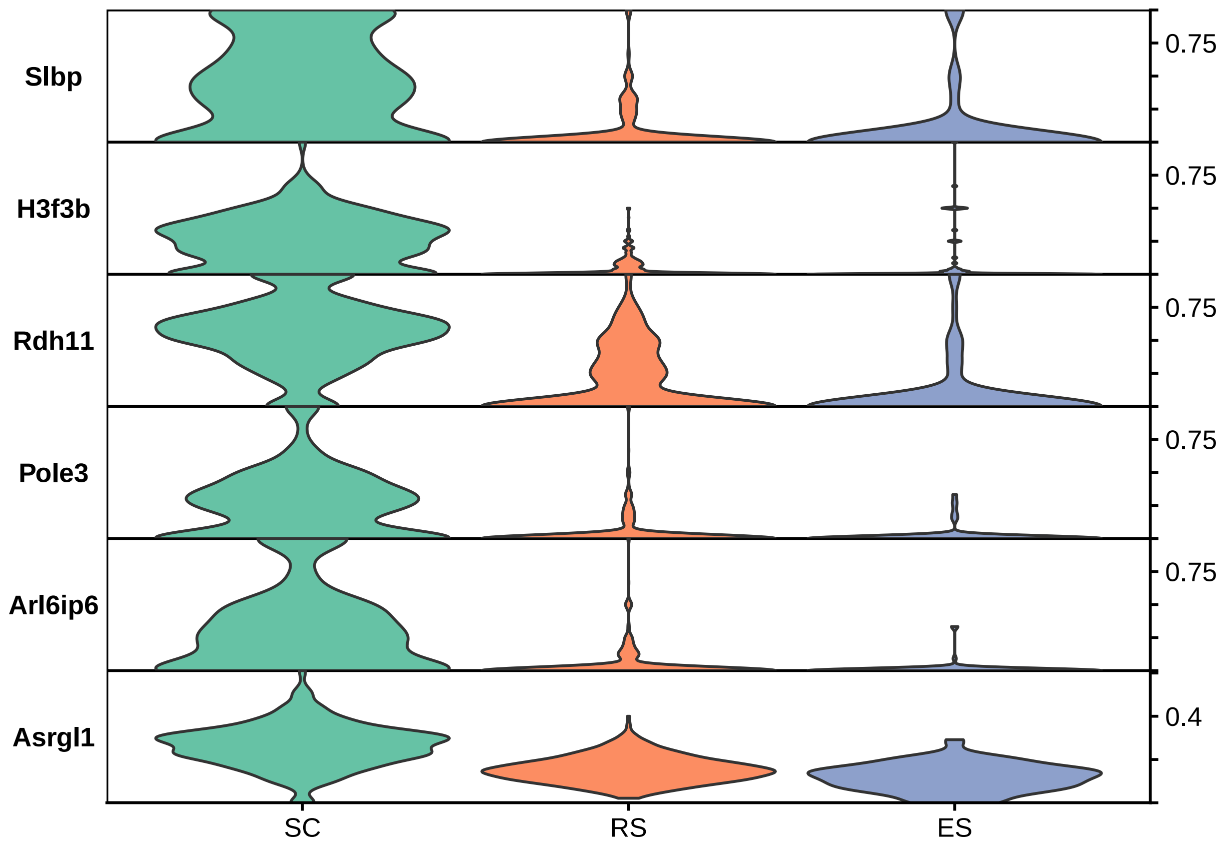


```r
#eoffice::topptx(filename = 'figures.pptx', title="markers_violin",
#                 width = 8, height = 6, append=TRUE)
```

It is easy to plot other types of figures to show these markers.

```r
# we can plot only two cell types, here SC and RS
vizAPAMarkers(RUD, group='celltype',
              markers=markers,
              selGroups = c('SC','RS'),
              figType = 'violin')
```

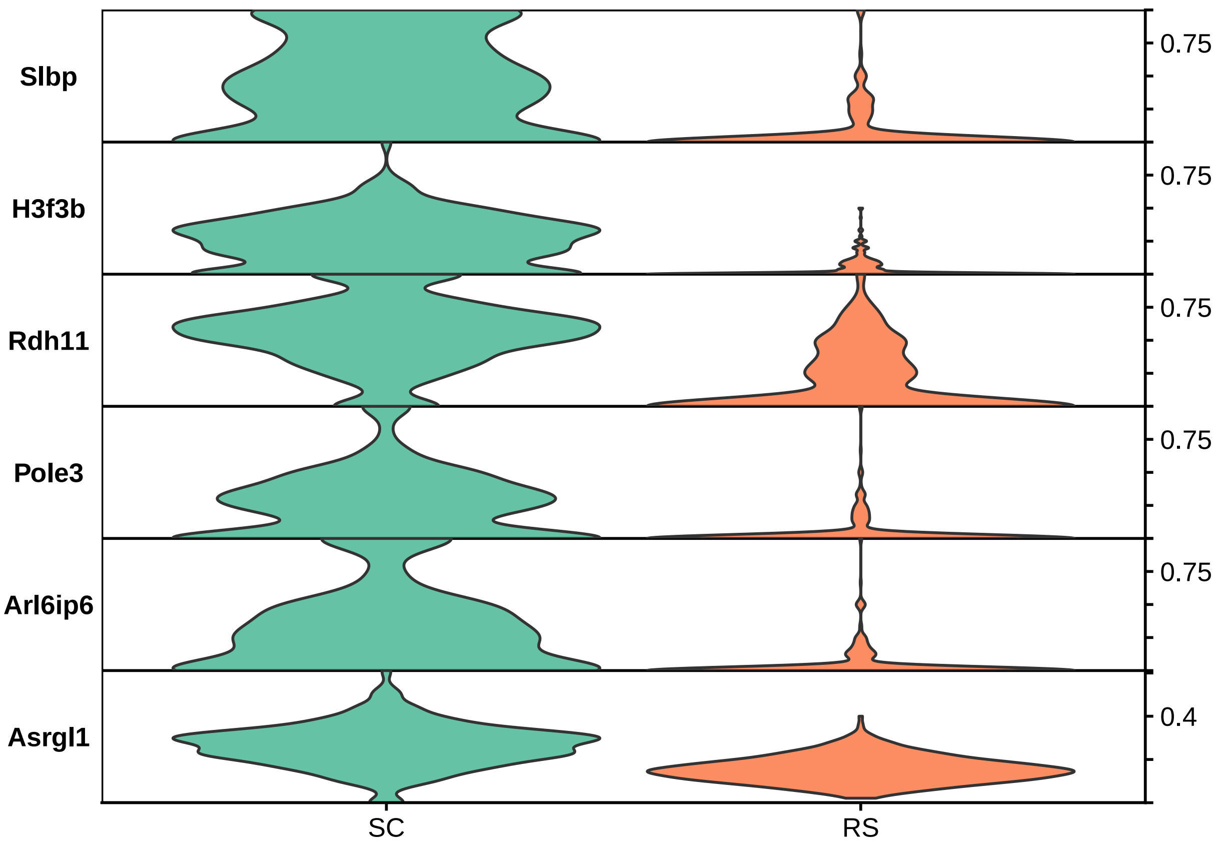


```
# Plot a heatmap for APA markers
vizAPAMarkers(RUD, group='celltype',
              markers=markers,
              figType = 'heatmap')
```

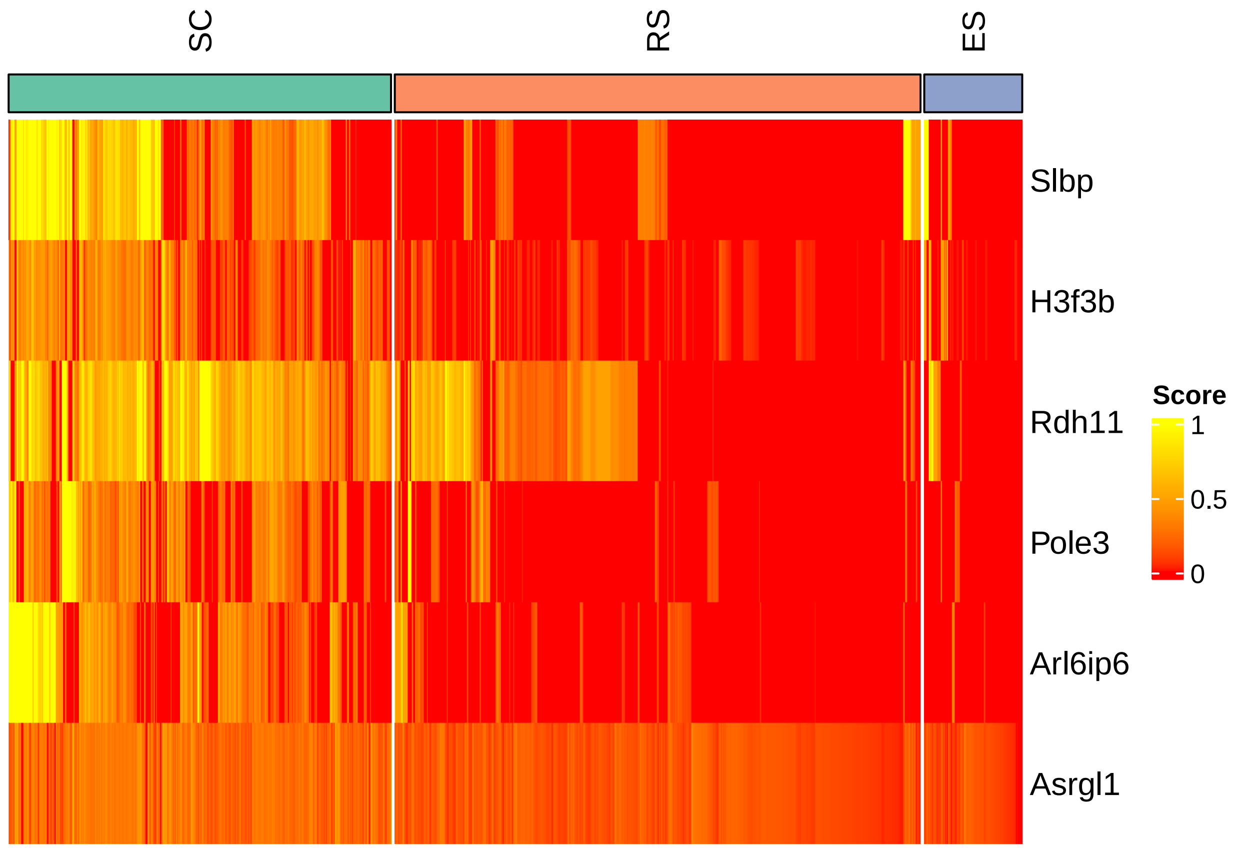


```r
# Plot a bubble plot for markers
vizAPAMarkers(RUD, group='celltype',
              markers=markers,
              figType = 'bubble')
```

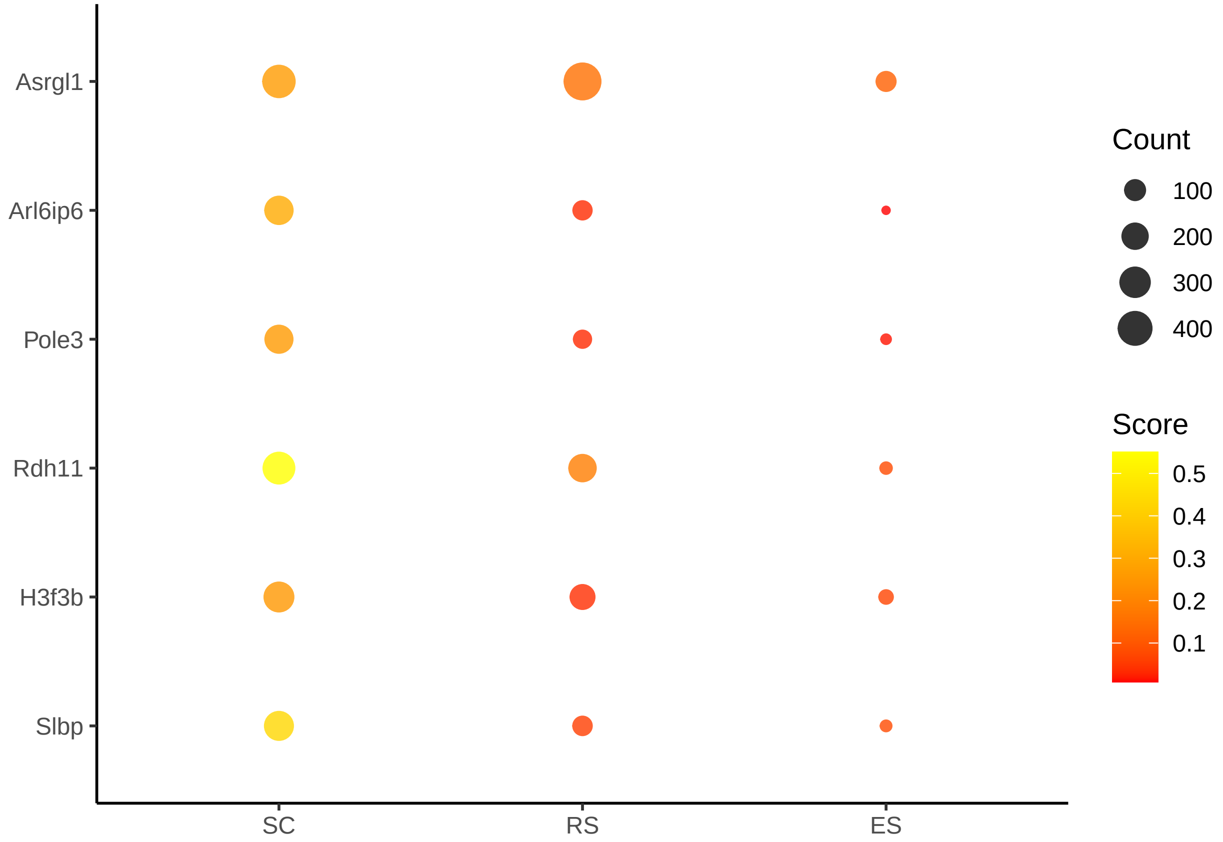


```r
# Switch the x/y axis of the bubble plot
vizAPAMarkers(RUD, group='celltype',
              markers=markers,
              figType = 'bubble',
              statTheme=list(xgroup=FALSE))
```

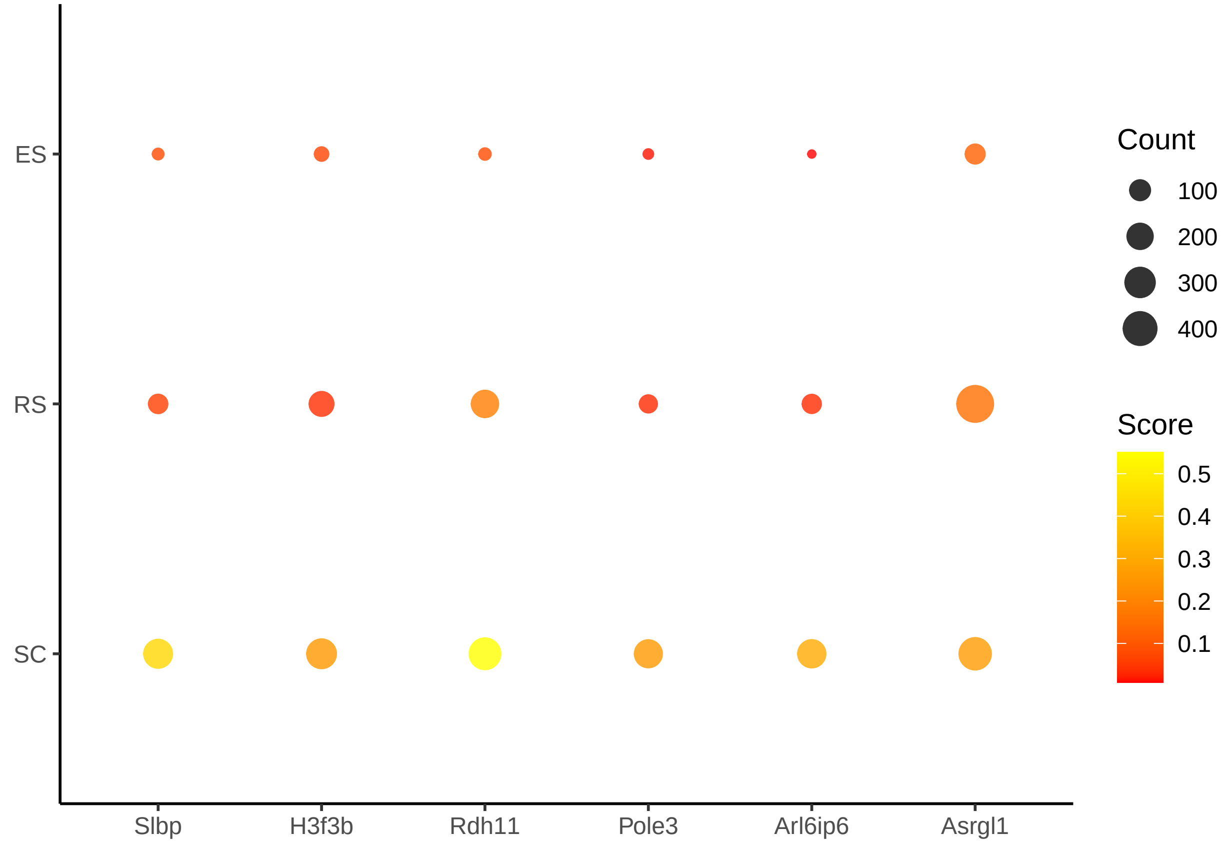

Next, we can plot UMAP for these APA markers. It is clear that the mean RUD scores of these markers in the SC group is much higher than in the RS group.

```
# Plot the UMAP plot
vizAPAMarkers(RUD,
              group='celltype',
              markers=markers,
              figType="umap",
              umap.x='UMAP_1', umap.y='UMAP_2')
```

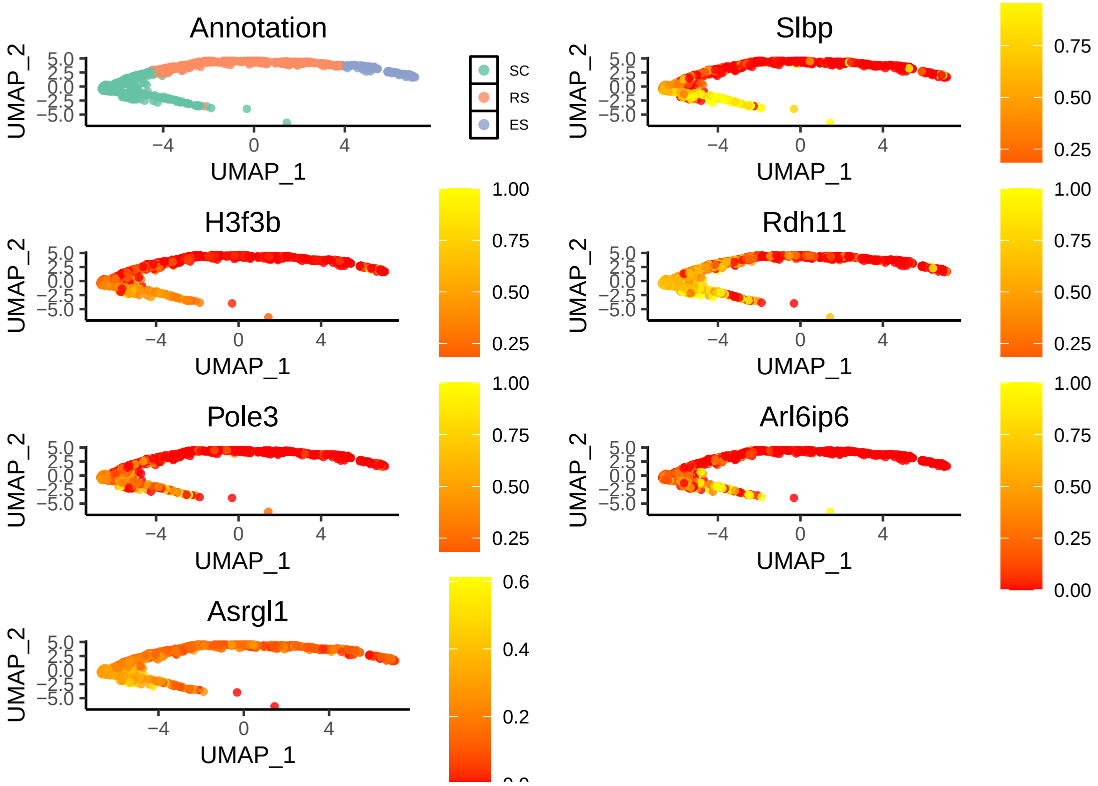

Visualize single APA marker

`vizAPAMarkers` is used for visualizing multiple APA markers; When only one marker is provided, we can use `vizStats` instead.

```
vizStats(RUD, group='celltype', figType="dot", gene=markers[1])
```

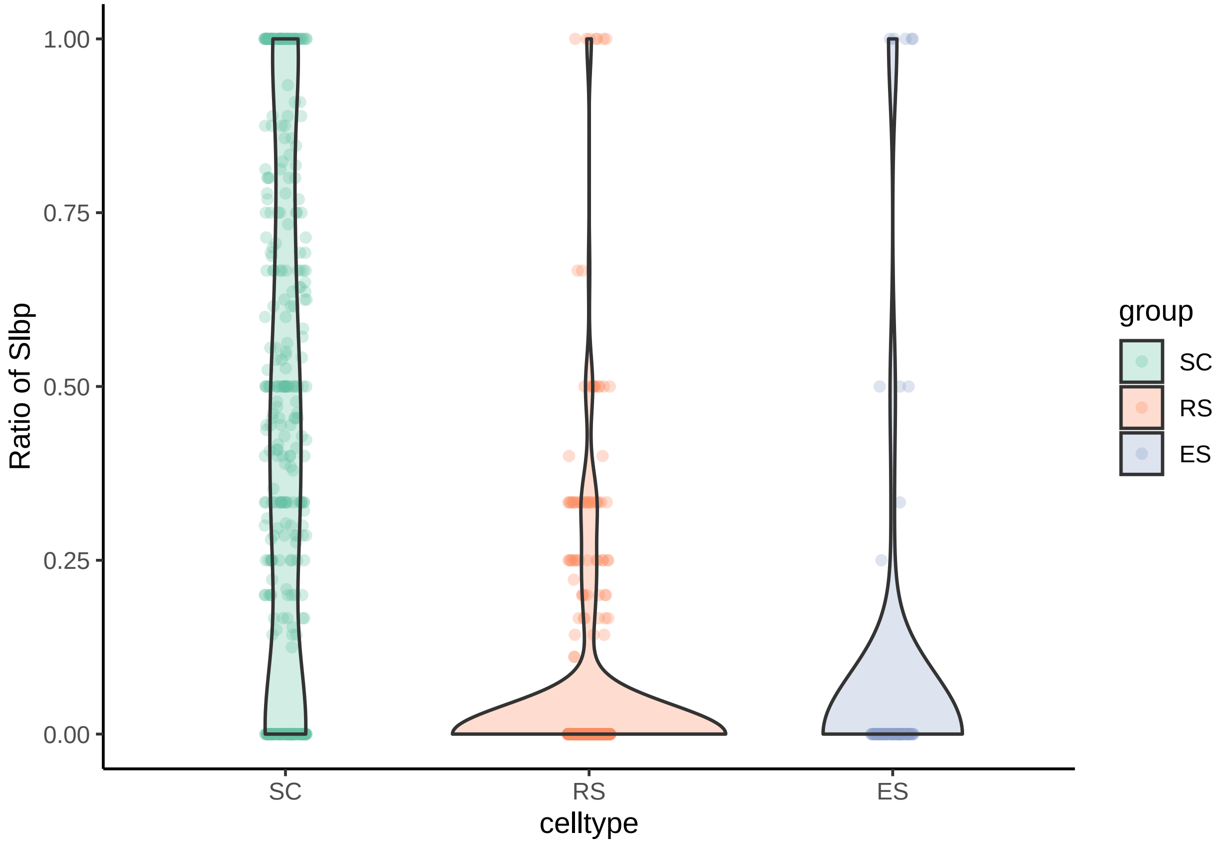


```
## To plot only the two cell types involved in the marker
vizStats(RUD, group='celltype', figType="dot", gene=markers[1],
         selGroups=c('SC','RS'))
```

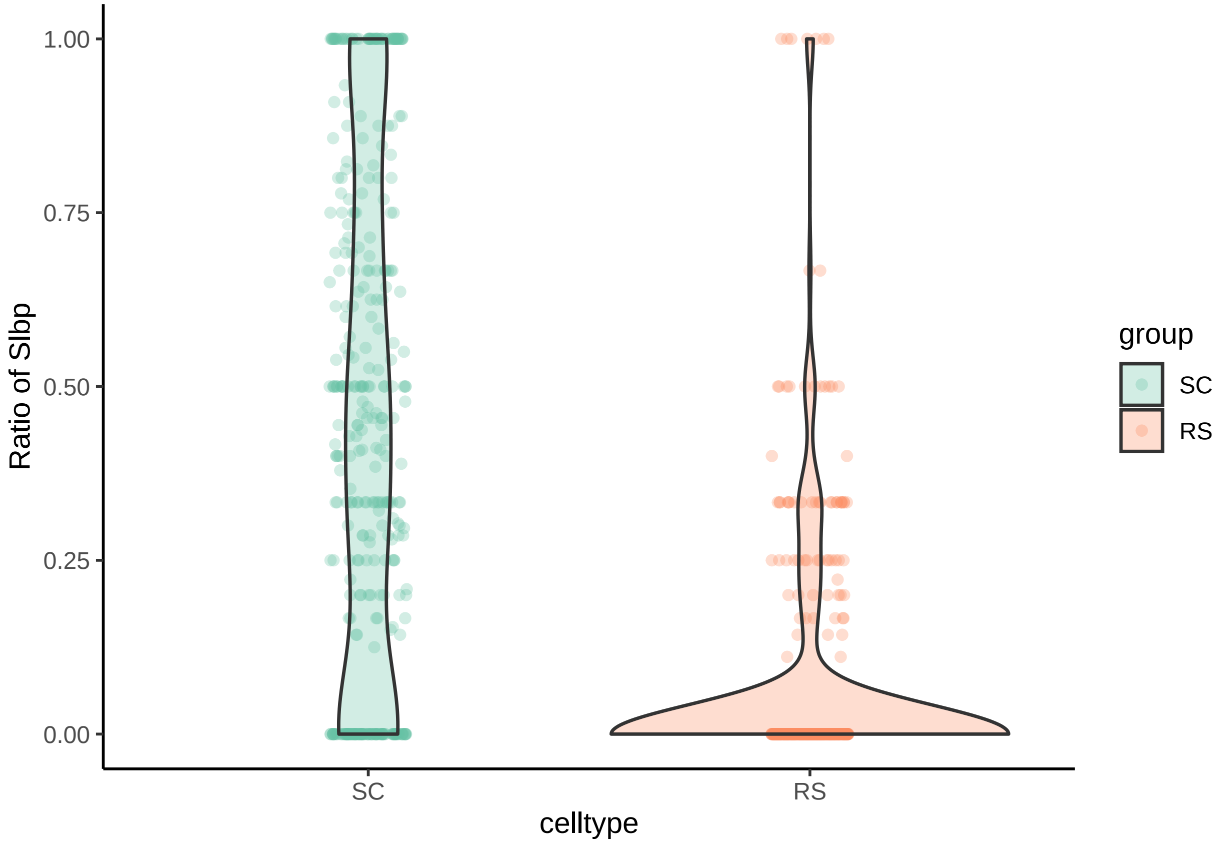


```r
## plot a heatmap to average RUD values across cell types
vizStats(RUD, group='celltype', figType="heatmap", gene=markers[1])
```

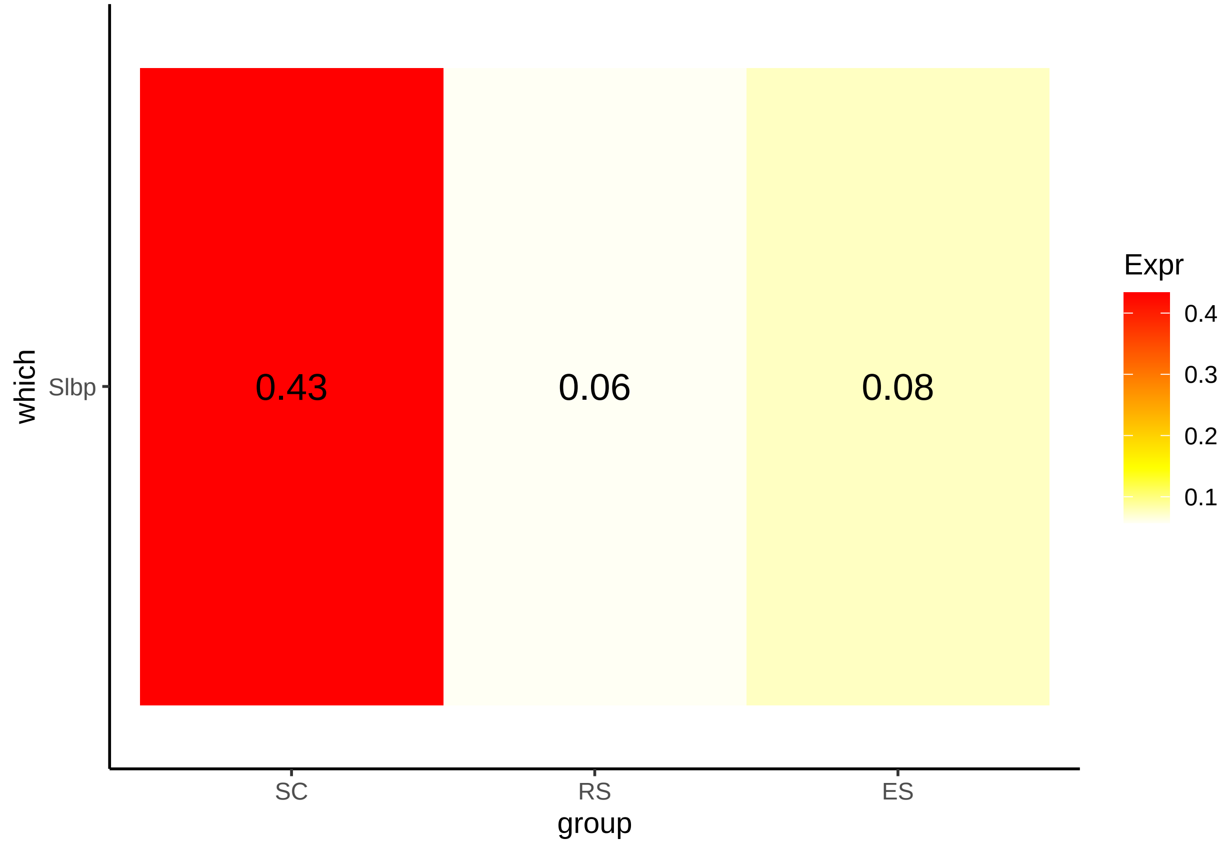

APA markers for one cell type

The above examples detect markers between every pair of cell types. It is also possible to compare one cell type with all other cells. Here we detect markers between SC and all other cells, retaining those markers only with higher RUD in SC (`only.pos=TRUE`).

```
m=getAPAmarkers(RUD,  group='celltype', cluster1='SC', only.pos = TRUE)
```

```
## PACds row = gene, PACds dataType = ratio
## It seems that PACds is APA ratio,
## will apply FindMarkers (default is wilcox-test) on the APA index
## to get DE APA events (each row is an APA gene).
```

```
head(m)
```

```
##          p_val avg_log2FC pct.1 pct.2    p_val_adj cluster1 cluster2   rowid
## 1 6.548303e-79  0.4079611 0.931 0.400 2.704449e-76       SC   non-SC   Rdh11
## 2 7.890439e-77  0.4340236 0.722 0.149 3.258751e-74       SC   non-SC    Slbp
## 3 2.590270e-75  0.3424751 0.678 0.132 1.069782e-72       SC   non-SC Arl6ip6
## 4 3.711976e-74  0.2877021 0.661 0.115 1.533046e-71       SC   non-SC   Pole3
## 5 1.388778e-71  0.2646926 0.785 0.331 5.735652e-69       SC   non-SC   H3f3b
## 6 1.914996e-68  0.2617507 0.653 0.076 7.908935e-66       SC   non-SC   Gmcl1
##   direction
## 1  positive
```

```
## 2  positive
## 3  positive
## 4  positive
## 5  positive
## 6  positive
```

```r
countAPAmarkers(m, plot=F)
```

```
## # A tibble: 1 x 3
##   pair      direction Count
##   <chr>     <chr>     <int>
## 1 SC~non-SC positive     22
```

Similarly, we can plot top markers. Here we used violin plot to show the top 10 markers for demonstration. It can be seen that these markers are all with higher RUD scores in SC.

```r
vizAPAMarkers(RUD, group='celltype',
              markers=m$rowid[1:10],
              figType = 'violin')
```

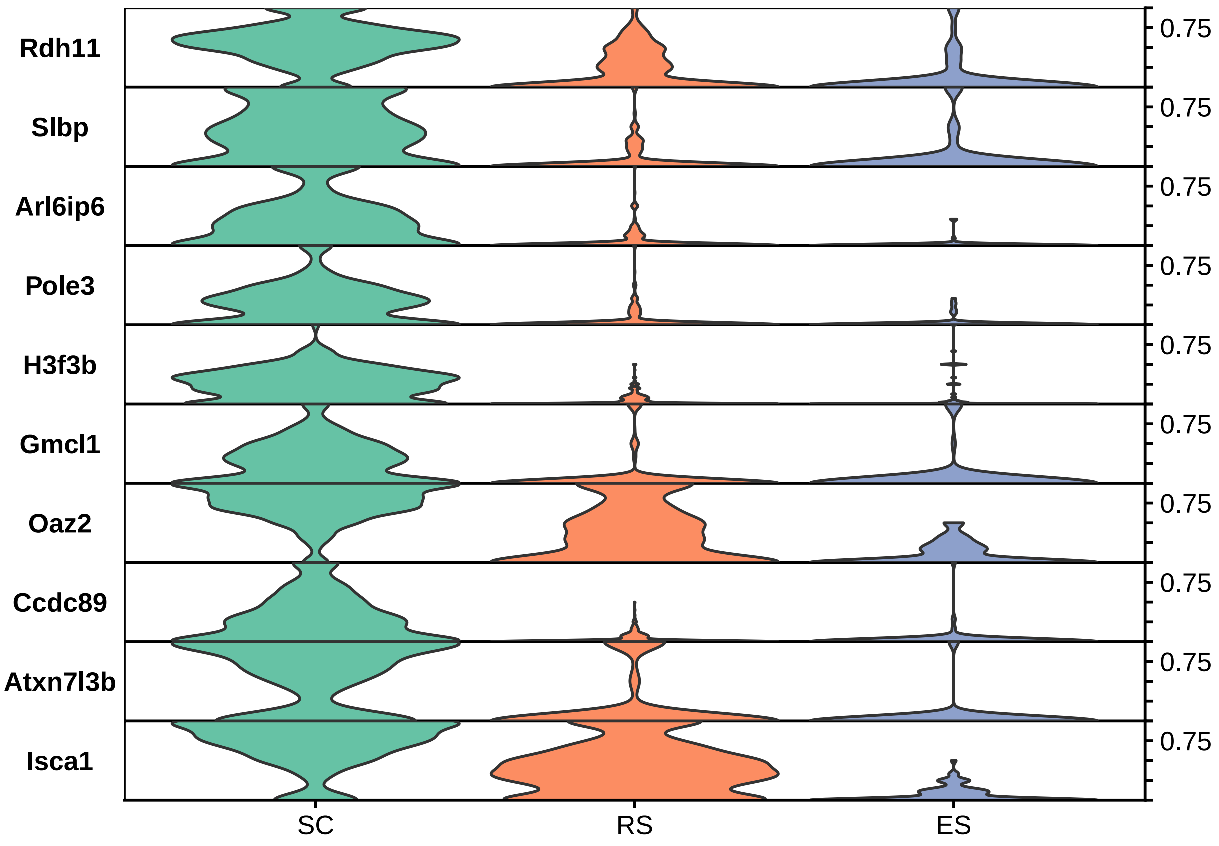

vizTracks to plot gene model, pAs and BAM tracks

One unique feature of vizAPA is plotting a genome-browser-like plot, including gene models, pA positions and BAM coverages.

Prepare BAM files

The BAM files and the corresponding index (.bai) files for the following analysis can be downloaded from the GitHub site of vizAPA: mouse.sperm.bam. For demonstration, these BAM files contain only three genes [66514 (Asrgl1), 21463 (Tcp11), 27058 (Srp9)] extracted from the original BAM file (accession number: GSM2803334).

```
#Create the list of BAM files
bam.files=c("dedup_GSM2803334.ES.mini.sorted.bam",
            "dedup_GSM2803334.RS.mini.sorted.bam",
            "dedup_GSM2803334.SC.mini.sorted.bam")
bam.groups=c("ES","RS","SC")
bam.labels=c("ES" ,"RS","SC")
bam.path='./'

# here we set bam.order to make BAM colors consistent with PACdataset
bams<-readBAMFileNames(bam.files=bam.files,
                       bam.path=bam.path,
                       bam.labels = bam.labels,
                       bam.groups = bam.groups,
                       bam.order=c('SC','RS','ES'))
bams
```

```
##                                  fileName group label
## 3 ./dedup_GSM2803334.SC.mini.sorted.bam    SC    SC
## 2 ./dedup_GSM2803334.RS.mini.sorted.bam    RS    RS
## 1 ./dedup_GSM2803334.ES.mini.sorted.bam    ES    ES
```

Load genome annotation to an annoHub

In vizAPA, the genome annotation is used for the track plots to show gene models in a genomic region. The genome annotation could be retrieved from several sources, including gff3/gtf file, TxDb, EnsDb, BioMart, and OrganismDb. Users can provide one or more annotation sources.

We can make an `annoHub` object storing different annotation sources, which can be used by many functions in vizAPA. In the following, we used the `TxDB` annotation for demonstration.

```
annoSource=new("annoHub")
library(TxDb.Mmusculus.UCSC.mm10.knownGene, quietly = TRUE)
txdb=TxDb.Mmusculus.UCSC.mm10.knownGene
annoSource=addAnno(annoSource, txdb)
annoSource
```

```
## @annos [annotation sources]:
## txdb=TxDb
## @defaultAnno:
##  txdb
```

Plot tracks for a specified gene

Having prepared the `PACdataset`, `annoHub`, and BAM files, we can easily plot an example gene (Asrgl1), with gene model, pA coordinates, and BAM coverages.

```r
vizTracks(gene=geneid,
          bams=bams,
          PACds.list=list(PA=scPACds),
          PA.show=c("pos"),
          cells=TRUE, cells.group='celltype',
          cells.width=200, cells.annoCoord=NULL,
          cells.method=c('sum'), cells.sort=c('group'),
          annoSource=annoSource,
          PA.columns="coord", PA.width=10,
          space5=0, space3=100,
          )
```

```
## Plot tracks for region: chr19:-:9109768:9135636
## Get gene model track from annoSource[ txdb ]...
## Get PACds track...
## chr19:-:9109768:9135636
## Get cells track...
## Get BAM tracks...
```

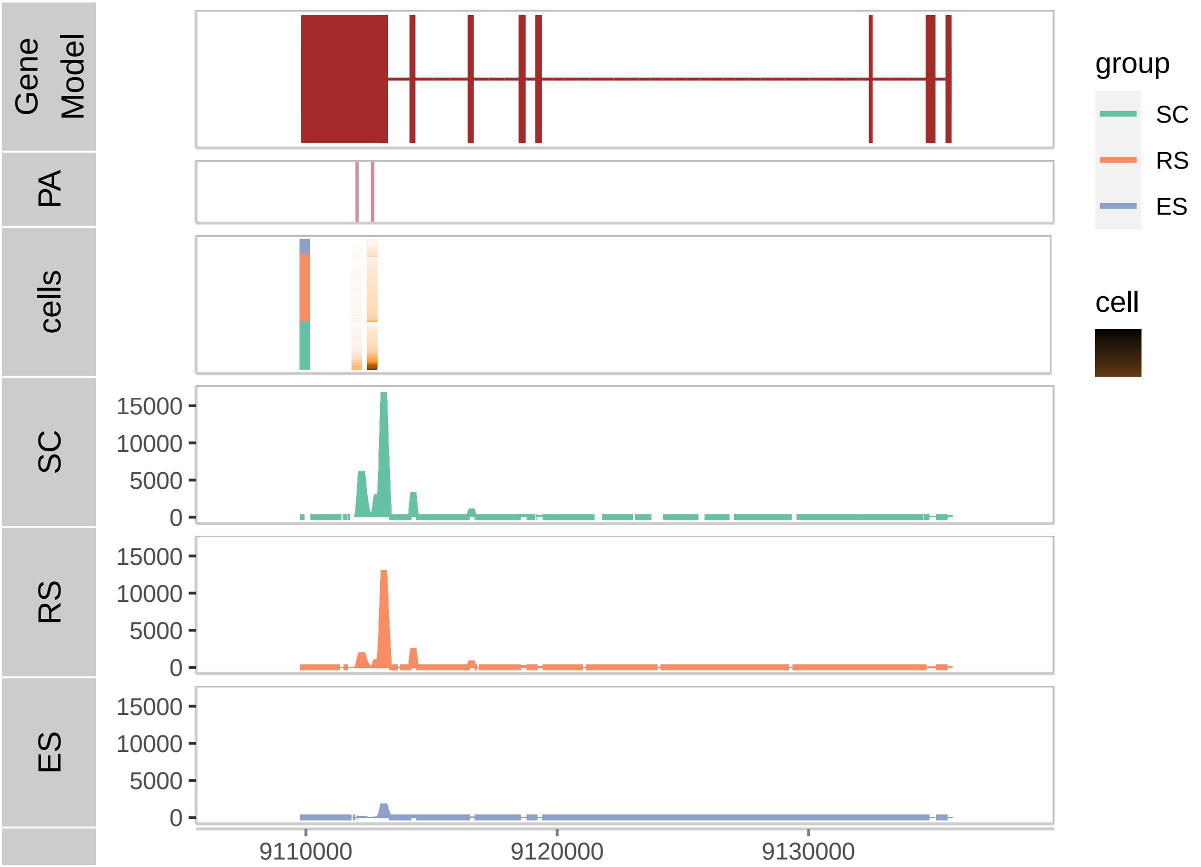

We can also plot and customize individual tracks separately and then combine all tracks together.

```r
# gene model track
gmtrack=getTrackGeneModel(gene=geneid, annoSource=annoSource, title=gene)
```

```r
# PA track
pactrack=getTrackPACds(scPACds, gene=geneid,
                       PA.show=c("pos"),
                       PA.columns="coord", PA.width=10,
                       title='PA')
```

```r
# we can customize the track using ggplot2's grammer
# pactrack$PA=pactrack$PA+ggplot2::theme_minimal()

# cell track
celltrack<-getTrackCells(scPACds, group='celltype', gene=geneid,
                          sortMethod='sum', sortCells='group', log=FALSE,
                          PA.columns="coord", PA.width=50, title='sum.cells')

# bam track
genomicRegion=getGenesRange(gene=geneid, annoSource, rt='list')
# add 1000 bp to both ends of the region
genomicRegion$start=genomicRegion$start-1000
genomicRegion$end=genomicRegion$end+1000
genomicRegion=paste0(unlist(genomicRegion), collapse=':')
bamtrack<-getTrackBams(bams, genomicRegion=genomicRegion)

# combine tracks
tks<-c(gmtrack, pactrack, celltrack, bamtrack)
names(tks)[1:3]=c(gene, 'pA', 'cells')
ggbio::tracks(tks)
```

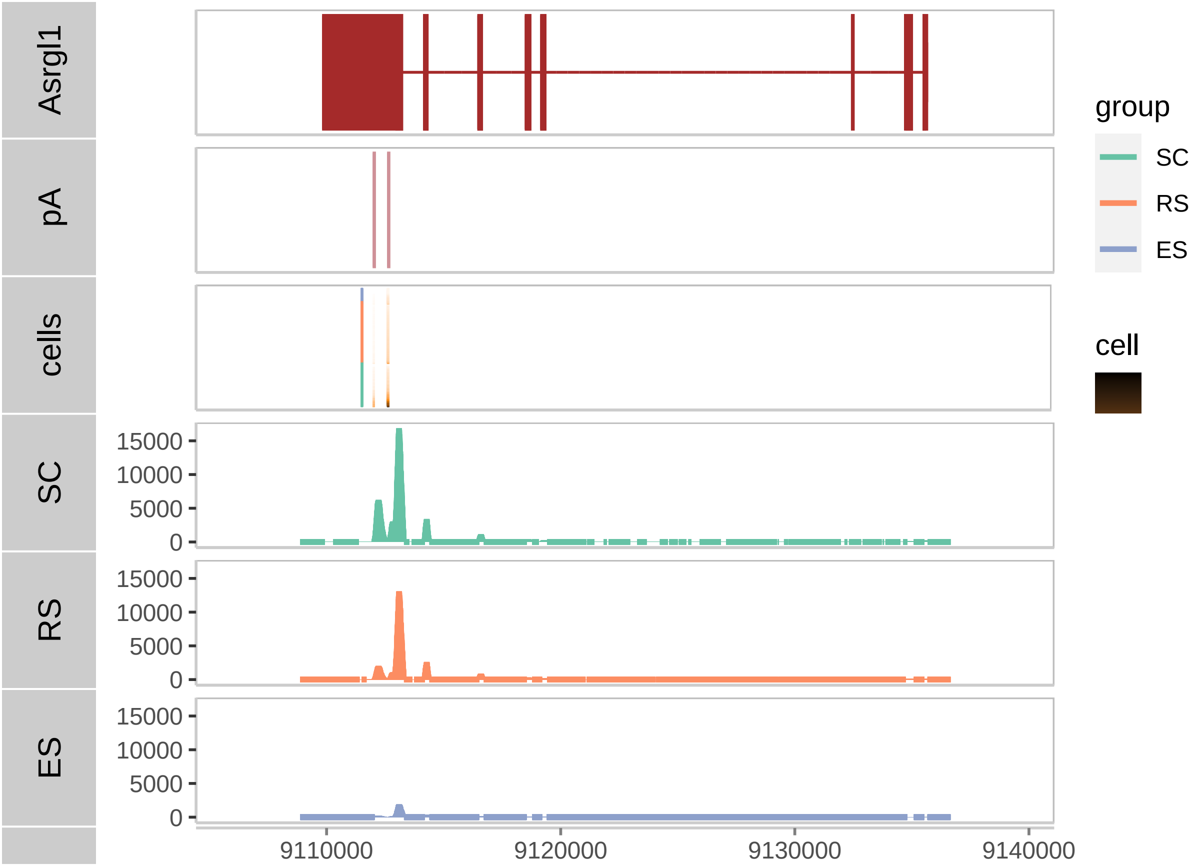

We can zoom in on a genomic region by specifying `xlim` and set the height of each track. For example, here we only display the 3'UTR area.

```
# plot legend 'horizontal' or 'vertical'
ggbio::tracks(tks, heights=c(0.7, 0.7, 1.2, 1.2, 1.2, 1.2),
              xlim=c(9111000, 9113500))+
  ggplot2::theme(legend.direction = 'vertical')
```

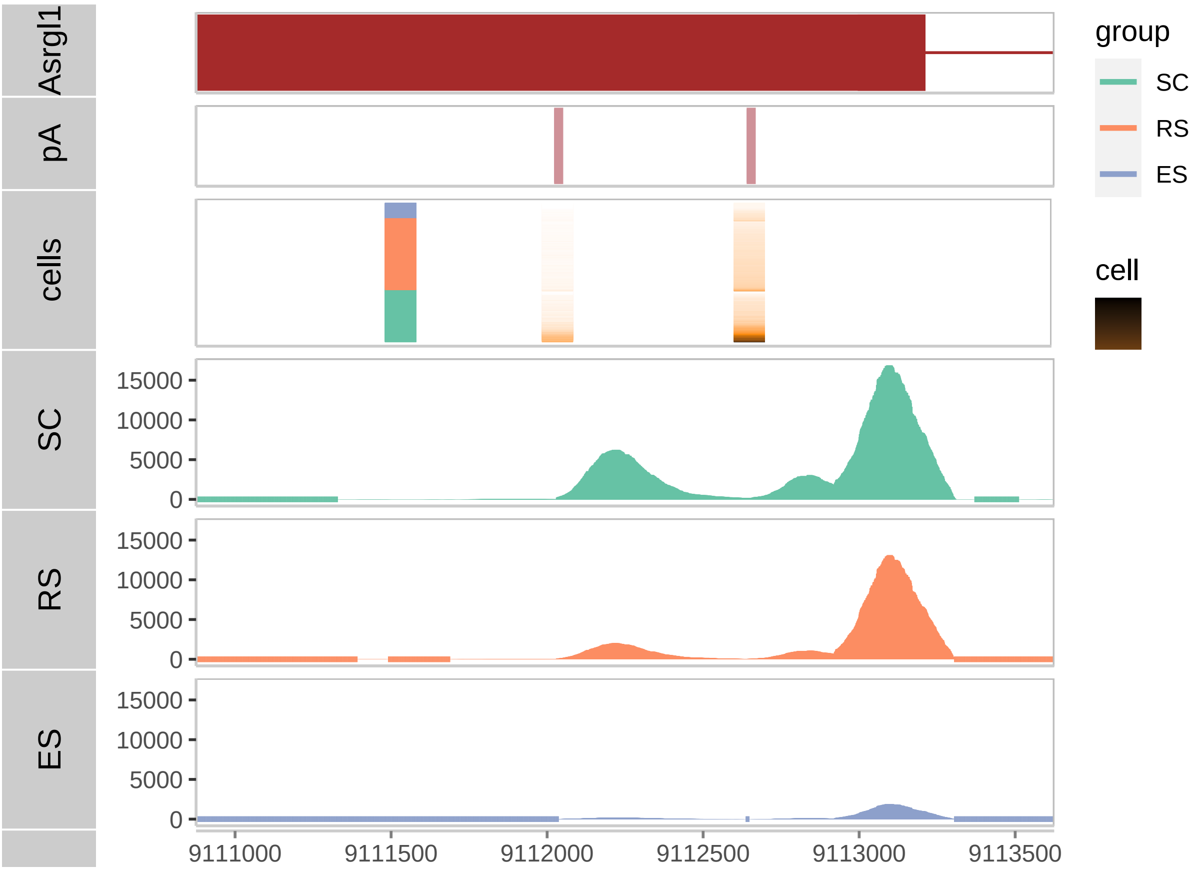


```
#eoffice::topptx(filename = 'figures.pptx', title="tracks",
#                 width = 8, height = 8, append=TRUE)
```

Session information

The session information records the versions of all the packages used in the generation of the present document.

```
sessionInfo()
```

```
## R version 4.2.2 (2022-10-31 ucrt)
## Platform: x86_64-w64-mingw32/x64 (64-bit)
## Running under: Windows 10 x64 (build 22621)
##
## Matrix products: default
##
## locale:
## [1] LC_COLLATE=Chinese (Simplified)_China.utf8
## [2] LC_CTYPE=Chinese (Simplified)_China.utf8
## [3] LC_MONETARY=Chinese (Simplified)_China.utf8
## [4] LC_NUMERIC=C
## [5] LC_TIME=Chinese (Simplified)_China.utf8
##
```

```
## attached base packages:
## [1] stats4    stats     graphics  grDevices utils     datasets  methods
## [8] base
##
## other attached packages:
##  [1] Mus.musculus_1.3.1
##  [2] TxDb.Mmusculus.UCSC.mm10.knownGene_3.10.0
##  [3] org.Mm.eg.db_3.16.0
##  [4] GO.db_3.16.0
##  [5] OrganismDbi_1.40.0
##  [6] GenomicFeatures_1.50.2
##  [7] GenomicRanges_1.50.1
##  [8] GenomeInfoDb_1.34.9
##  [9] AnnotationDbi_1.60.0
## [10] IRanges_2.32.0
## [11] S4Vectors_0.36.0
## [12] Biobase_2.58.0
## [13] BiocGenerics_0.44.0
## [14] vizAPA_0.1.0
##
## loaded via a namespace (and not attached):
##   [1] rappdirs_0.3.3             rtracklayer_1.58.0
##   [3] scattermore_0.8            GGally_2.1.2
##   [5] SeuratObject_4.1.3         tidyr_1.2.1
##   [7] ggplot2_3.4.0              bit64_4.0.5
##   [9] knitr_1.41                 irlba_2.3.5.1
##  [11] DelayedArray_0.24.0        data.table_1.14.6
##  [13] rpart_4.1.19               KEGGREST_1.38.0
##  [15] RCurl_1.98-1.9             AnnotationFilter_1.22.0
##  [17] doParallel_1.0.17          generics_0.1.3
##  [19] movAPA_0.2.0               cowplot_1.1.1
##  [21] RSQLite_2.2.18             RANN_2.6.1
##  [23] future_1.30.0              bit_4.0.5
##  [25] spatstat.data_3.0-0        xml2_1.3.3
##  [27] httpuv_1.6.6               SummarizedExperiment_1.28.0
##  [29] assertthat_0.2.1           xfun_0.35
##  [31] hms_1.1.2                  evaluate_0.18
##  [33] promises_1.2.0.1           fansi_1.0.3
##  [35] restfulr_0.0.15            progress_1.2.2
##  [37] dbplyr_2.2.1               igraph_1.3.5
##  [39] DBI_1.1.3                  htmlwidgets_1.5.4
##  [41] reshape_0.8.9              spatstat.geom_3.0-3
##  [43] purrr_1.0.2                ellipsis_0.3.2
##  [45] ggnewscale_0.4.8           dplyr_1.0.10
##  [47] ggpubr_0.5.0               backports_1.4.1
##  [49] biomaRt_2.54.0             deldir_1.0-6
##  [51] MatrixGenerics_1.10.0      vctrs_0.6.5
##  [53] Cairo_1.6-0                ensembldb_2.22.0
##  [55] ROCR_1.0-11                abind_1.4-5
##  [57] cachem_1.0.6               withr_2.5.0
##  [59] BSgenome_1.66.2            progressr_0.12.0
##  [61] checkmate_2.1.0            sctransform_0.3.5
##  [63] GenomicAlignments_1.34.0   prettyunits_1.1.1
##  [65] goftest_1.2-3              cluster_2.1.4
```

```
##  [67] lazyeval_0.2.2              crayon_1.5.2
##  [69] spatstat.explore_3.0-5      pkgconfig_2.0.3
##  [71] labeling_0.4.2              nlme_3.1-160
##  [73] ProtGenerics_1.30.0         nnet_7.3-18
##  [75] rlang_1.1.2                 globals_0.16.2
##  [77] lifecycle_1.0.3             miniUI_0.1.1.1
##  [79] filelock_1.0.2              BiocFileCache_2.6.0
##  [81] dichromat_2.0-0.1           polyclip_1.10-4
##  [83] matrixStats_0.63.0          lmtest_0.9-40
##  [85] graph_1.76.0                Matrix_1.5-3
##  [87] carData_3.0-5               zoo_1.8-11
##  [89] base64enc_0.1-3             ggridges_0.5.4
##  [91] GlobalOptions_0.1.2         png_0.1-7
##  [93] viridisLite_0.4.1           rjson_0.2.21
##  [95] bitops_1.0-7                KernSmooth_2.23-20
##  [97] Biostrings_2.66.0           blob_1.2.3
##  [99] shape_1.4.6                 stringr_1.4.1
## [101] spatstat.random_3.0-1       parallelly_1.33.0
## [103] jpeg_0.1-10                 rstatix_0.7.1
## [105] ggsignif_0.6.4              scales_1.2.1
## [107] memoise_2.0.1               magrittr_2.0.3
## [109] plyr_1.8.8                  ica_1.0-3
## [111] zlibbioc_1.44.0             compiler_4.2.2
## [113] BiocIO_1.8.0                RColorBrewer_1.1-3
## [115] clue_0.3-63                 fitdistrplus_1.1-8
## [117] Rsamtools_2.14.0            cli_3.6.1
## [119] XVector_0.38.0              listenv_0.9.0
## [121] patchwork_1.1.2             pbapply_1.6-0
## [123] htmlTable_2.4.1             Formula_1.2-4
## [125] MASS_7.3-58.1               tidyselect_1.2.0
## [127] stringi_1.7.8               highr_0.9
## [129] yaml_2.3.6                  latticeExtra_0.6-30
## [131] ggrepel_0.9.2               grid_4.2.2
## [133] VariantAnnotation_1.44.0    tools_4.2.2
## [135] future.apply_1.10.0         parallel_4.2.2
## [137] circlize_0.4.15             rstudioapi_0.14
## [139] foreach_1.5.2               foreign_0.8-83
## [141] gridExtra_2.3               farver_2.1.1
## [143] Rtsne_0.16                  digest_0.6.30
## [145] BiocManager_1.30.19         shiny_1.7.3
## [147] Rcpp_1.0.9                  car_3.1-1
## [149] broom_1.0.2                 later_1.3.0
## [151] RcppAnnoy_0.0.20            httr_1.4.4
## [153] ggbio_1.46.0                biovizBase_1.46.0
## [155] ComplexHeatmap_2.14.0       colorspace_2.0-3
## [157] XML_3.99-0.12               tensor_1.5
## [159] reticulate_1.30             splines_4.2.2
## [161] uwot_0.1.14                 RBGL_1.74.0
## [163] spatstat.utils_3.0-1        sp_1.5-1
## [165] plotly_4.10.1               xtable_1.8-4
## [167] jsonlite_1.8.3              R6_2.5.1
## [169] Hmisc_5.0-0                 pillar_1.9.0
## [171] htmltools_0.5.3             mime_0.12
## [173] glue_1.6.2                  fastmap_1.1.0
```

```
## [175] BiocParallel_1.32.1         codetools_0.2-18
## [177] utf8_1.2.2                  lattice_0.20-45
## [179] spatstat.sparse_3.0-0       tibble_3.2.1
## [181] curl_4.3.3                  leiden_0.4.3
## [183] magick_2.7.3                interp_1.1-3
## [185] limma_3.54.0                survival_3.4-0
## [187] rmarkdown_2.18              munsell_0.5.0
## [189] GetoptLong_1.0.5            GenomeInfoDbData_1.2.9
## [191] iterators_1.0.14            reshape2_1.4.4
## [193] gtable_0.3.1                Seurat_4.3.0
```
