# Supplementary material for: vizAPA: visualizing dynamics of alternative polyadenylation from bulk and single-cell data
Source: Bioinformatics. 2024 Mar 14;40(3):btae099. doi: 10.1093/bioinformatics/btae099 (PMC10950478; doi:10.1093/bioinformatics/btae099)
Supplement: btae099_Supplementary_Data [file btae099_supplementary_data.zip › vizAPA supplementary material.pdf]

vizAPA: visualizing dynamics of alternative polyadenylation from bulk and single-cell data

Supplementary Material

Schema of vizAPA

The vizAPA package mainly consists of six modules (Supplementary Fig. S1).

(1) Data input module. vizAPA imports different types of APA data through a unified interface. Poly(A) sites from bulk samples or single cells obtained by different tools or pipelines can be stored in a compact data structure *PACdataset*. BAM files with read alignment information are also stored, with each file corresponding to one cell type or biological sample.

(2) Genome annotation module. vizAPA builds internally a very flexible data structure called *annoHub*, which can adapt easily to different genome annotation sources from different species. This enables automatic unification of gene IDs and chromosome names among different data sources, i.e., *PACdataset*, BAM files, and different genome annotations. Even for species without any publicly available genome annotation, it is allowed providing a customized file recording region information of poly(A) sites instead.

(3) Visualization module *vizTracks*. This module generates a genome-browser-like plot, which utilizes tracks to display different types of information related to APA, including gene models, positions and expression levels or usages of poly(A) sites, and read alignments along genomic coordinates in individual cell groups. Particularly, an additional track placing heat maps with gradient colors is also provided to represent single-cell read coverage, which helps decipher cell-to-cell heterogeneity of poly(A) site usages within homologous cell populations. The track plot is highly flexible in that tracks in the plot can be easily reordered or customized, through an internal parameter container called *vizTHEME*.

(4) Visualization module *vizStats*. This module visualizes usages (read counts or ratio) of any given poly(A) site(s) (e.g., proximal and distal sites) in a gene across different cell groups. Different types of plots are provided, including heatmap, violin plot, boxplot, bubble plot, dot plot, and heatmap. The plot can be easily customized through an internal parameter container called *statTHEME*.

(5) Visualization module *vizUMAP*. The nonlinear dimensional reduction technique UMAP was utilized to learn two-dimensional embeddings for visualizing clusters of cells with similar APA expression or usage profiles. Moreover, the (mean) gene expression level, poly(A) site expression level, and APA usage of given genes or poly(A) sites can be overlaid on the UMAP embeddings to discern cell-to-cell and cluster-to-cluster heterogeneity of poly(A) site usages.

(6) Visualization module *vizAPAmarkers*. First, a certain APA metric, such as Relative Usage of Distal poly(A) site (RUD) (Chen, et al., 2018; Ji, et al., 2009; Wu, et al., 2021) or other metrics (Ye, et al.,

2021), can be used for quantifying APA site usage of a gene (or 3′ UTR lengthening/shortening). Then the FindMarkers function in Seurat (Stuart, et al., 2019) is employed to identify genes with differential APA usages (called APA markers), which applies statistical tests, including Wilcoxon Rank Sum test, Student's t-test, and likelihood ratio test on two groups of APA scores. Finally, rich plots, including violin plot, heat map, bubble plot, and UMAP plot, are provided for the visualization of APA usages of selected APA markers across cell groups.

Overall, VizAPA is an independent R package that is easy to install and run, which provides several unified visualization interfaces, including VizStat, VizUMAP, and VizTracks, for different purposes of APA data visualization. **Supplementary Table S1** compares functions between vizAPA and other visualization tools, including PolyAMiner-Bulk (Jonnakuti, et al., 2023), UCSC Genome Browser (Nassar, et al., 2023), Millefy (Ozaki, et al., 2020). APA-Scan (Fahmi, et al., 2022), scDAPA (Ye, et al., 2019), movAPA (Ye, et al., 2021), VALERIE (Wen, et al., 2020), and RNA-Scoop (Stephenson, et al., 2021).

Use of vizAPA

vizAPA is available at https://github.com/BMILAB/vizAPA.

Three user manuals of vizAPA are provided as Supplementary Material:

(1) vizAPA_user_mannual_1_A_minimal_tutorial.pdf
This tutorial takes a *PACdataset* object storing a list of poly(A) sites as input and describes how to quickly get started with vizAPA.

(2) vizAPA_user_mannual_2_Using_vizStats_vizUMAP_vizAPAmarkers.pdf
This tutorial describes full usages of series functions related to *vizStats*, *vizUMAP*, and *vizAPAmarkers* in vizAPA. These functions are used for making statistics and visualization of poly(A) read counts and APA usages across cells or cell types.

(3) vizAPA_user_mannual_3_Using_vizTracks.pdf
This tutorial takes a *PACdataset* object storing a list of poly(A) sites and BAM files as input and describes full usages of series functions related to *vizTracks* in vizAPA. The vizTracks function generates genome-browser-like plot, which utilizes tracks to display different types information related to APA.

Additional user manuals describing how to read and analyze data generated by single-cell APA identification tools -- Sierra and scAPAtrap with vizAPA are available at vizAPA’s GitHub site. All data used in the manual and more examples can also be found there.

Supplementary Figures

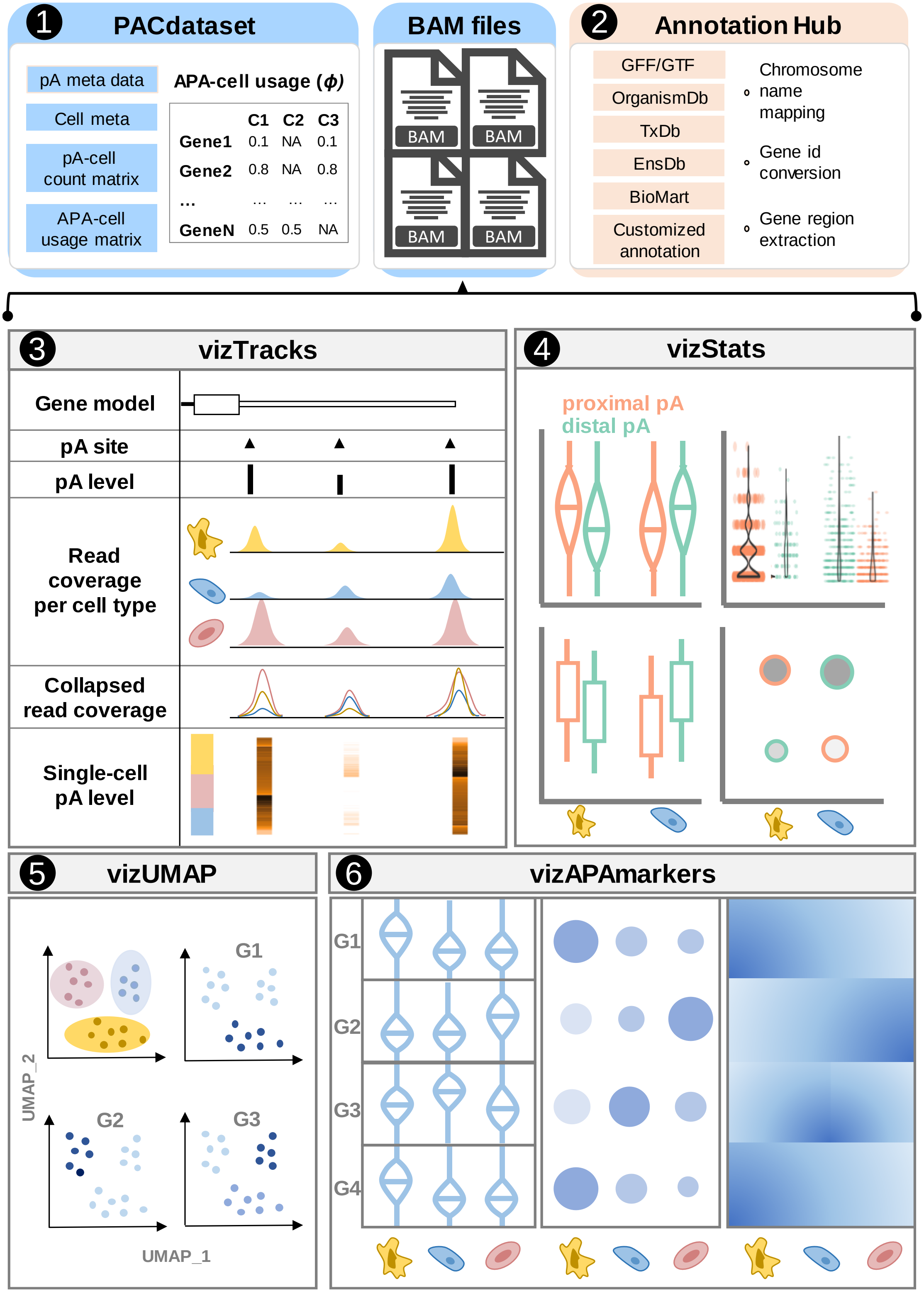

Figure S1. Schema of vizAPA. (1) Data input module; (2) genome annotation module; (3) visualization module *vizTracks*; (4) visualization module *vizStats*; (5) visualization module *vizUMAP*; (6) visualization module *vizAPAmarkers*. pA, polyadenylation site; G, gene.

References

Chen, M., *et al.* 3' UTR lengthening as a novel mechanism in regulating cellular senescence. *Genome Res.* 2018.

Fahmi, N.A., *et al.* APA-Scan: detection and visualization of 3'-UTR alternative polyadenylation with RNA-seq and 3'-end-seq data. *BMC Bioinf.* 2022;23(Suppl 3):396.

Ji, Z., *et al.* Progressive lengthening of 3' untranslated regions of mRNAs by alternative polyadenylation during mouse embryonic development. *Proc. Natl. Acad. Sci. USA* 2009;106(17):7028-7033.

Jonnakuti, V.S., *et al.* PolyAMiner-Bulk: A Machine Learning Based Bioinformatics Algorithm to Infer and Decode Alternative Polyadenylation Dynamics from bulk RNA-seq data. *bioRxiv* 2023:2023.2001.2023.523471.

Nassar, L.R., *et al.* The UCSC Genome Browser database: 2023 update. *Nucleic Acids Res.* 2023;51(D1):D1188-d1195.

Ozaki, H., *et al.* Millefy: visualizing cell-to-cell heterogeneity in read coverage of single-cell RNA sequencing datasets. *BMC Genomics* 2020;21(1):177.

Stephenson, M., *et al.* RNA-Scoop: interactive visualization of transcripts in single-cell transcriptomes. *NAR Genom Bioinform* 2021;3(4):lqab105.

Stuart, T., *et al.* Comprehensive integration of single-cell data. *Cell* 2019;177(7):1888-1902.e1821.

Wen, W.X., Mead, A.J. and Thongjuea, S. VALERIE: Visual-based inspection of alternative splicing events at single-cell resolution. *PLoS Comput. Biol.* 2020;16(9):e1008195.

Wu, X., *et al.* scAPAtrap: identification and quantification of alternative polyadenylation sites from single-cell RNA-seq data. *Briefings Bioinf.* 2021;22(4).

Ye, C., *et al.* scDAPA: detection and visualization of dynamic alternative polyadenylation from single cell RNA-seq data. *Bioinformatics* 2019;36(4):1262-1264.

Ye, W., *et al.* movAPA: Modeling and visualization of dynamics of alternative polyadenylation across biological samples. *Bioinformatics* 2021;37(16):2470–2472.
