# Supplementary material for: vizAPA: visualizing dynamics of alternative polyadenylation from bulk and single-cell data
Source: Bioinformatics. 2024 Mar 14;40(3):btae099. doi: 10.1093/bioinformatics/btae099 (PMC10950478; doi:10.1093/bioinformatics/btae099)
Supplement: btae099_Supplementary_Data [file btae099_supplementary_data.zip › vizAPA_user_mannual_2_Using_vizStats_vizUMAP_vizAPAmarkers.pdf]

Using vizStats, vizUMAP, vizAPAmarkers in vizAPA: a full tutorial

Xiaohui Wu, Xingyu Bi, Wenbin Ye

2024-01-10

Contents

Overview **2**

Data preparation **2**

Demo PACdataset 2

`vizStats` to summarize pA usages across cell categories **3**

The example Odf4 gene 3

Violin plot 4

boxplot and bubble plot 6

Plot given pAs in a gene 9

Plot all pAs 10

Modify plot by `statTheme` 11

`vizUMAP` to plot 2D-embeddings **13**

UMAP plot for all genes 13

UMAP plot for given genes or pAs 15

`vizAPAmarkers` to visualize APA markers across cell categories **19**

Get APA markers by RUD index 19

Plot APA markers 20

UMAP plot for APA markers 27

Get APA markers based on other APA index 29

Get APA markers by read counts 31

Get APA markers for each cell type 33

Session information **35**

Overview

This tutorial takes a `PACdataset` object storing a list of poly(A) sites as input and describes full usages of series function related to `vizStats`, `vizUMAP`, and `vizAPAmarkers` in `vizAPA`.

Different from vizTracks which plots a IGV-like plot, `vizStats`, `vizUMAP`, and `vizAPAmarkers` are used for making statistics and visualization of pA read counts and APA usages across cells or cell types.

Data preparation

Demo PACdataset

In the package of `vizAPA`, there is a demo `PACdataset` object of mouse sperm cells, containing 974 pAs [poly(A) sites] from 413 genes. There are total 955 cells from three cell types (SC, Spermatocytes; RS, Round spermatids; ES, Elongating spermatids). This PACdataset has been annotated, with both pAs' and cells' meta data.

```
library(vizAPA)

data(scPACds, package='vizAPA')

# summary of the PACdataset
movAPA::summary(scPACds)
```

```
## PAC# 974
## sample# 955
## summary of expression level of each PA
##    Min. 1st Qu.  Median    Mean 3rd Qu.    Max.
##       1      72     957    3151    3636   96363
## summary of expressed sample# of each PA
##    Min. 1st Qu.  Median    Mean 3rd Qu.    Max.
##    1.00   64.25  452.50  452.05  810.00  955.00
## gene# 413
##      nPAC
## 3UTR  974
```

```
# cell meta data
head(scPACds@colData)
```

```
##                  orig.ident nCount_RNA nFeature_RNA RNA_snn_res.0.5
## AAACCTGAGCTTATCG       gene      23617         5061               9
## AAACCTGGTTGAGTTC       gene      19555         4802               9
## AAACCTGTCAACGAAA       gene      23467         5009               8
## AAACGGGCACAGGTTT       gene      28832         5484               8
## AAACGGGTCATTTGGG       gene      18931         4819               8
## AAACGGGTCCTCATTA       gene      15734         3855               8
##                  seurat_clusters celltype       UMAP_1      UMAP_2
## AAACCTGAGCTTATCG               9       RS  0.361751856 4.528803031
## AAACCTGGTTGAGTTC               9       RS -0.119255482 4.563224952
## AAACCTGTCAACGAAA               8       RS  3.023034156 4.074635188
## AAACGGGCACAGGTTT               8       RS  3.322863163  3.81046788
```

```
## AAACGGGTCATTTGGG               8       ES   4.73071772 3.419416826
## AAACGGGTCCTCATTA               8       ES  5.306060375 3.274766843
##                           barcode
## AAACCTGAGCTTATCG AAACCTGAGCTTATCG
## AAACCTGGTTGAGTTC AAACCTGGTTGAGTTC
## AAACCTGTCAACGAAA AAACCTGTCAACGAAA
## AAACGGGCACAGGTTT AAACGGGCACAGGTTT
## AAACGGGTCATTTGGG AAACGGGTCATTTGGG
## AAACGGGTCCTCATTA AAACGGGTCCTCATTA
```

It is better to change the level order of the factor `celltype` in scPACds, which can make the labels in the plots consistent with sperm differentiation from SC to RS to ES.

```
scPACds@colData$celltype=factor(scPACds@colData$celltype,
                                levels=c('SC','RS','ES'))
```

`vizStats` to summarize pA usages across cell categories

`vizStats` draws different types of plots, including boxplot, violin plot, dot plot, and bubble plot, to show coordinates and expression (pA count or APA ratio) of given pAs or pAs in a gene across different conditions (e.g., cell types).

In this tutorial, we take the Odf4 gene (entrez id=252868) as an example.

The example Odf4 gene

```
gene=252868

## show the pAs in this gene
scPACds@anno[scPACds@anno$gene==gene, c(1:6, 10:12)]
```

```
##           chr strand    coord    start      end  ftr   gene gene_start gene_end
## PA13514 chr11      - 68921835 68921835 68922175 3UTR 252868   68921835 68927049
## PA13515 chr11      - 68921238 68921238 68921652 3UTR 252868         NA       NA
```

```
## show expression level of each pAs
Matrix::rowSums(scPACds@counts[scPACds@anno$gene==gene, ])
```

```
## PA13514 PA13515
##    3777    1260
```

```
## show the gene model and pAs in a track plot
library(TxDb.Mmusculus.UCSC.mm10.knownGene, quietly = TRUE)
txdb=TxDb.Mmusculus.UCSC.mm10.knownGene
annoSource=new("annoHub")
annoSource=addAnno(annoSource, txdb)

# here gm.reduce=FALSE to show full features of gene model instead of collapsed
vizTracks(gene=gene,
```

```r
          PACds.list=list(pA=scPACds), PA.show=c("pos"),
          annoSource=annoSource,
          PA.columns="coord", PA.width=10,
          space5=1000, space3=1000,
          vizTheme=list(gm.reduce=FALSE))
```

```
## Plot tracks for region: chr11:-:68920835:68928081
## Get gene model track from annoSource[ txdb ]...
## Get PACds track...
## chr11:-:68920835:68928081
```

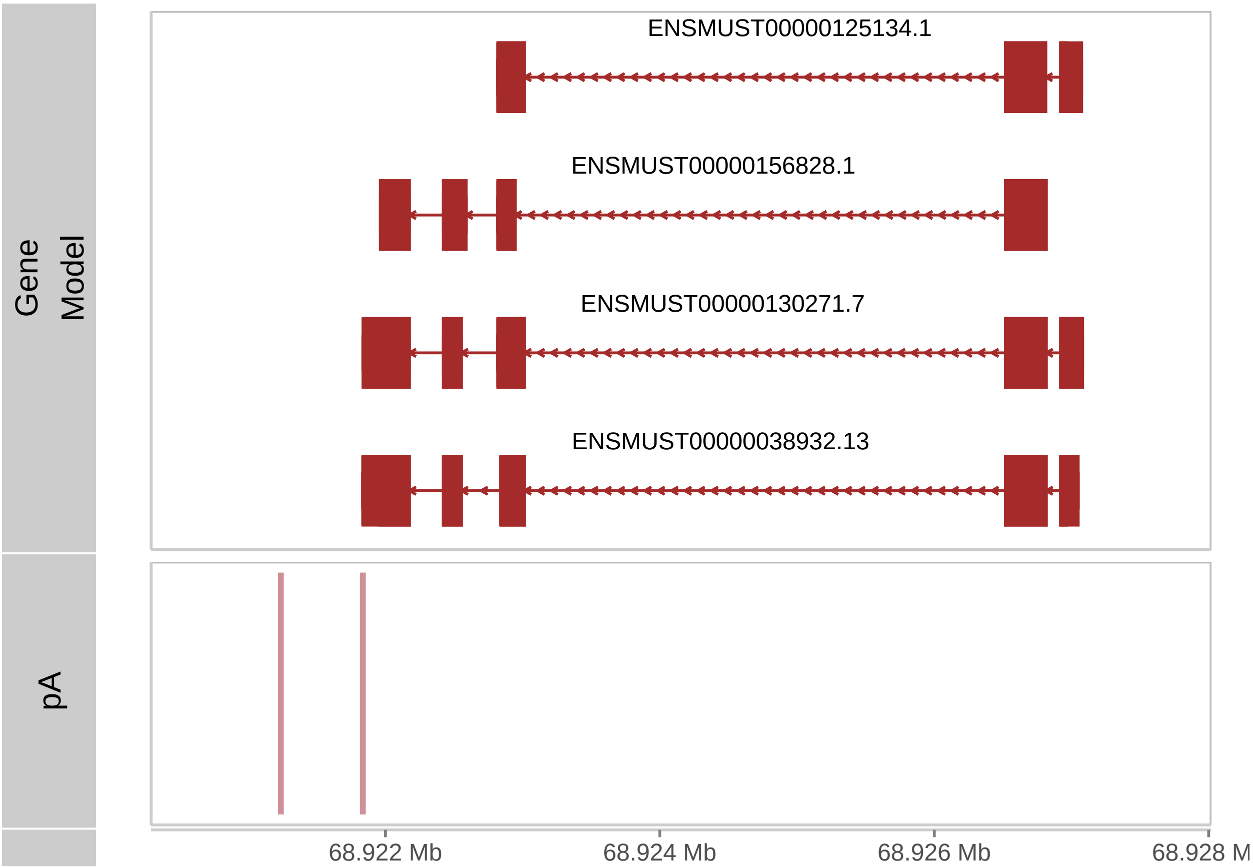

Violin plot

Here is an example to plot a violin plot to show the expression levels of pAs in a given gene across cell types.

```r
vizStats(scPACds, group='celltype', gene=gene, PAs=NULL, figType="violin")
```

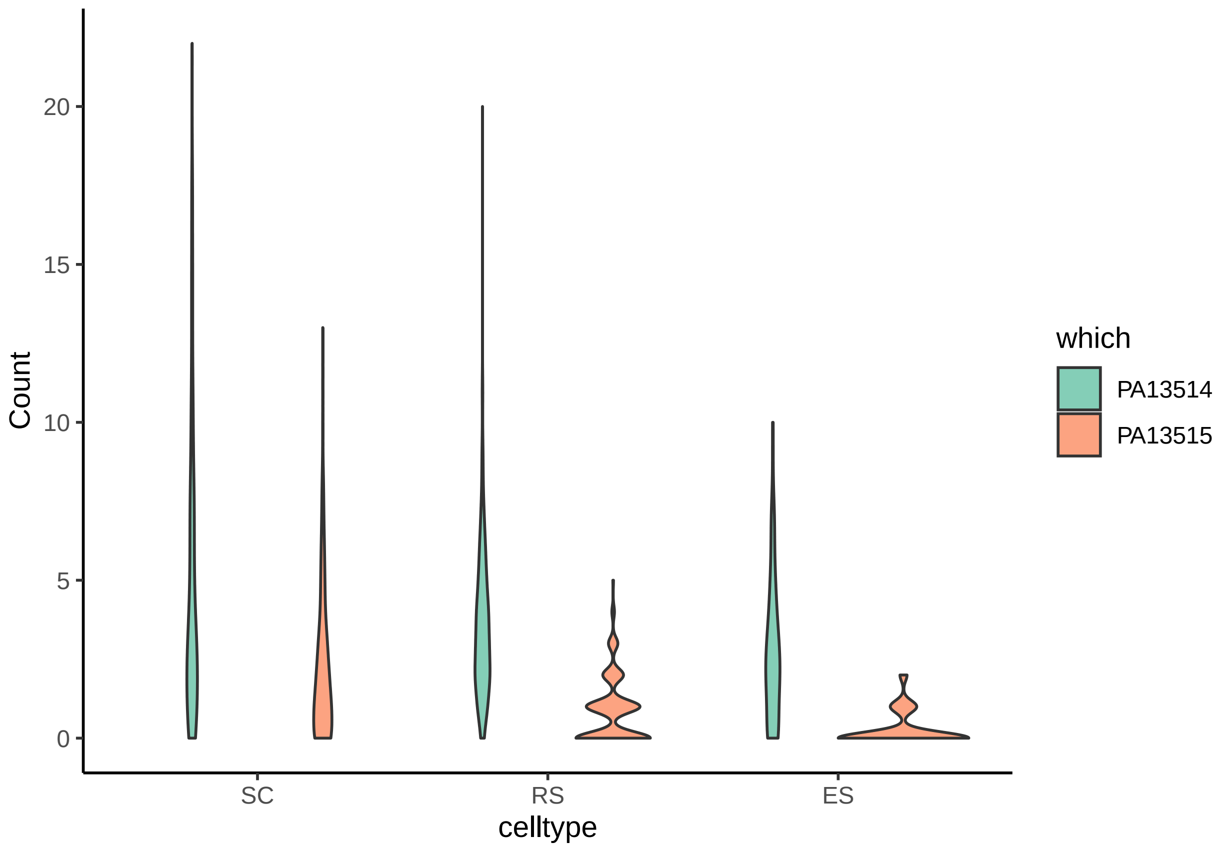

If the PACdataset's counts matrix is of count type, and it is difficult to see the expression distribution using the raw counts, we can log2 level instead.

```
vizStats(scPACds, group='celltype',
         gene=gene, PAs=NULL,
         figType="violin",
         log=TRUE)
```

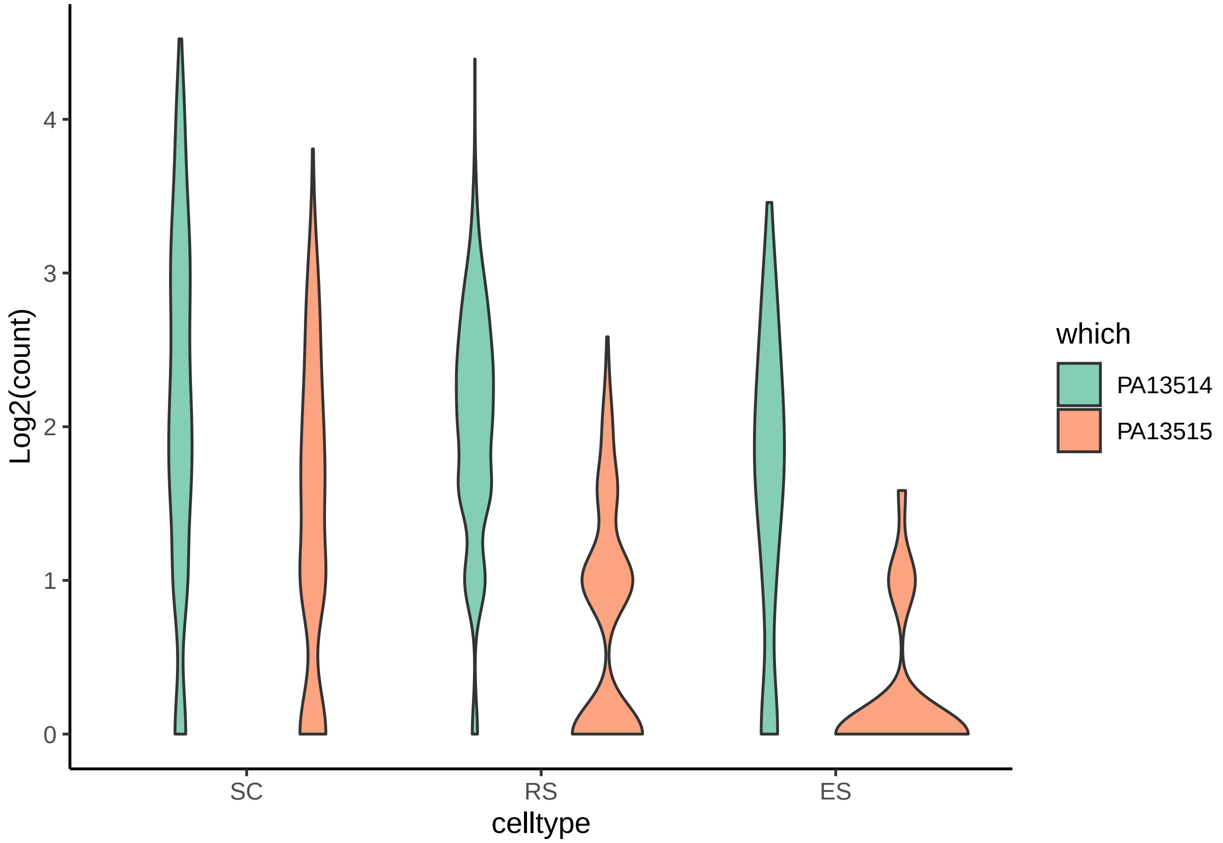

boxplot and bubble plot

Plot other types of plots.

```
# boxplot
vizStats(scPACds, group='celltype', gene=gene, PAs=NULL, figType="box")
```

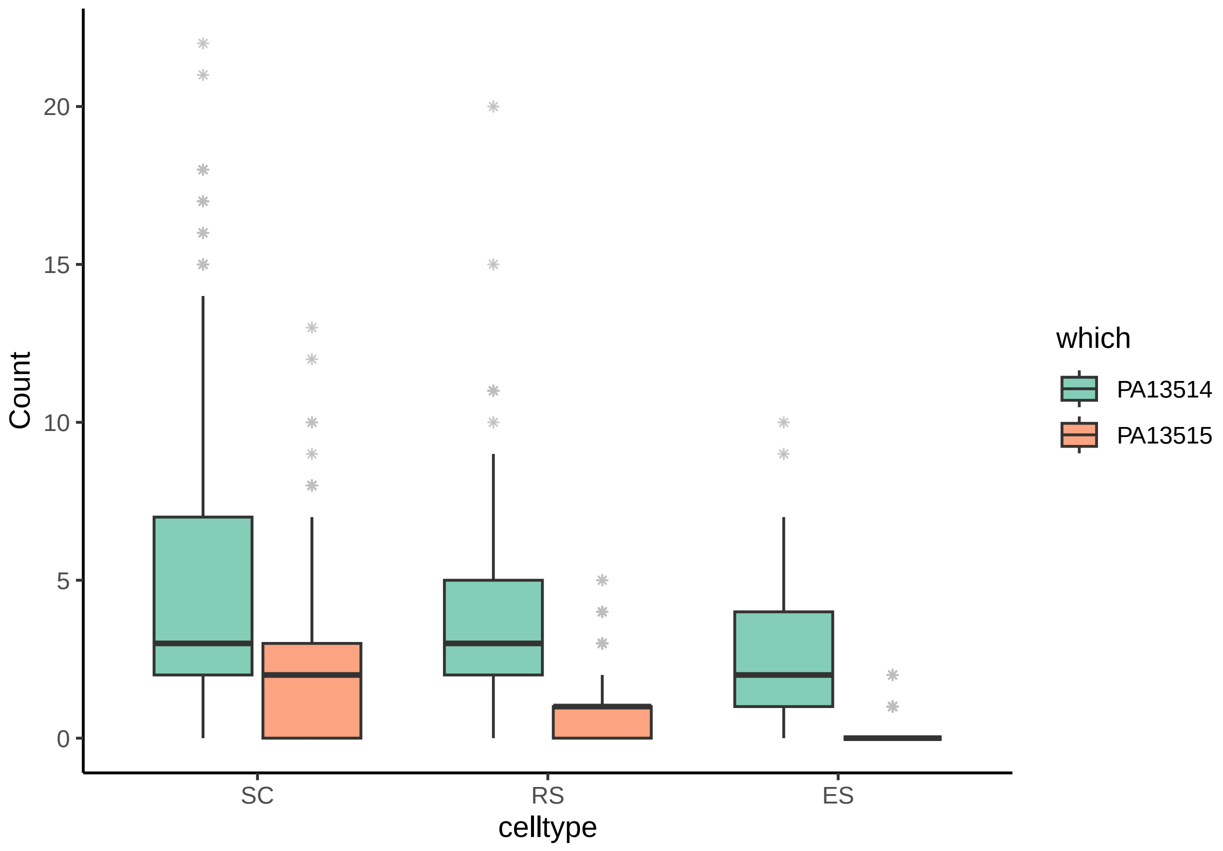


```
# violin plot with dots
vizStats(scPACds, group='celltype', gene=gene, PAs=NULL, figType="dot")
```

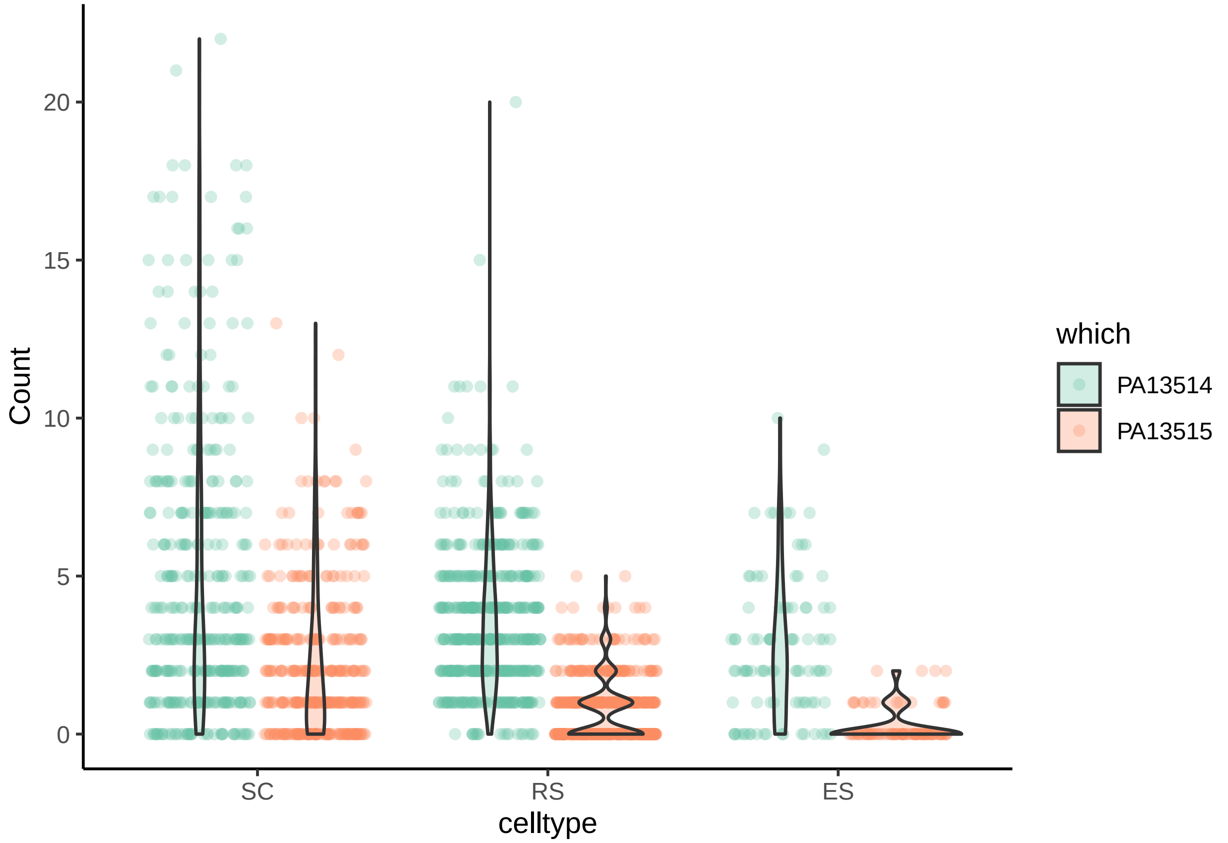


```r
# bubble plot
vizStats(scPACds, group='celltype', gene=gene, PAs=NULL,
         figType="bubble")
```

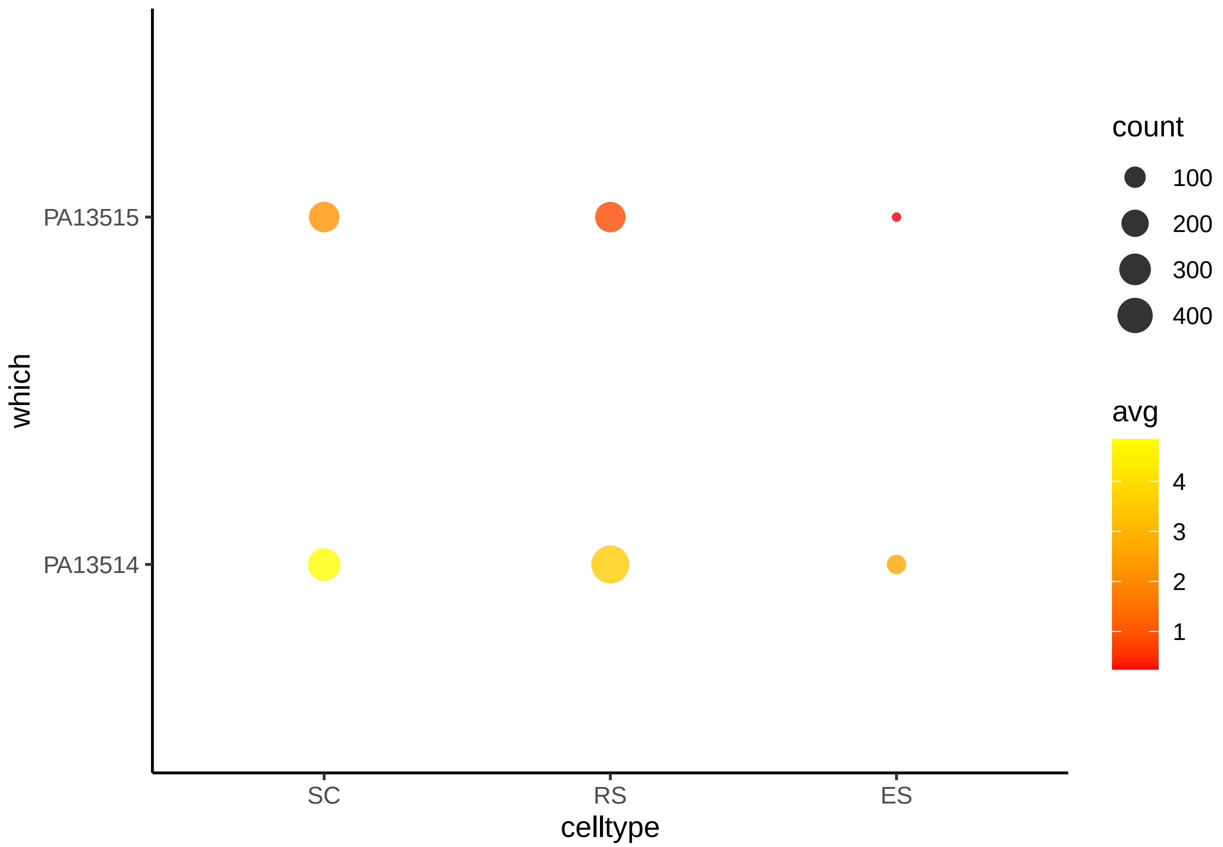

Plot given pAs in a gene

It is also able to show given PAs, by specifying the rowid of PAs in the PACdataset.

```
# For example, here we show two PAs in another gene
PAids=c('PA6085', 'PA6083')
vizStats(scPACds, group='celltype', PAs=PAids, figType="dot")
```

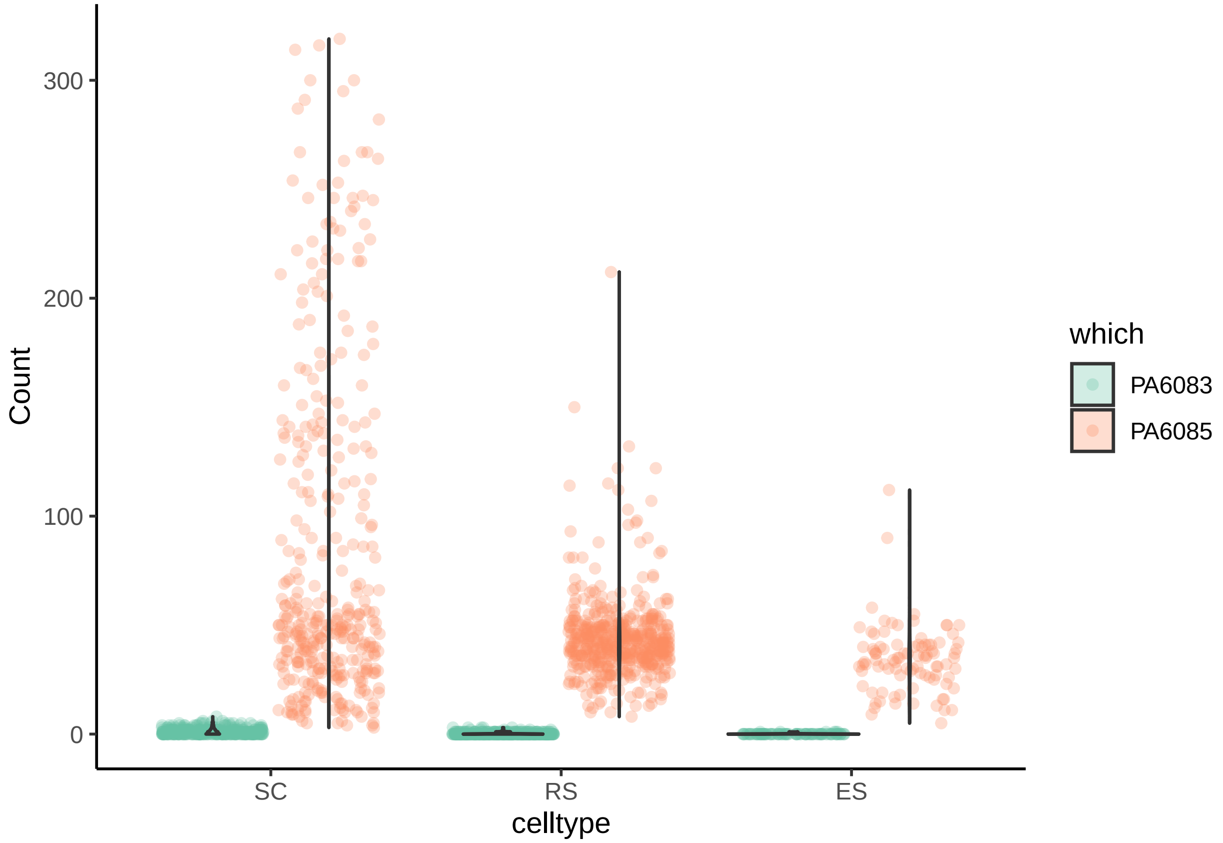

Plot all pAs

If no pA or gene is provided, then it is to plot the mean of all pAs (if it is a pA matrix) or genes (if it is a gene or APA index matrix) in the PACdataset. Here the `scPACds` is a PA-expression matrix, so `vizStats` plots the mean value of all pAs across cell types.

```
vizStats(scPACds, group='celltype', figType="violin")
```

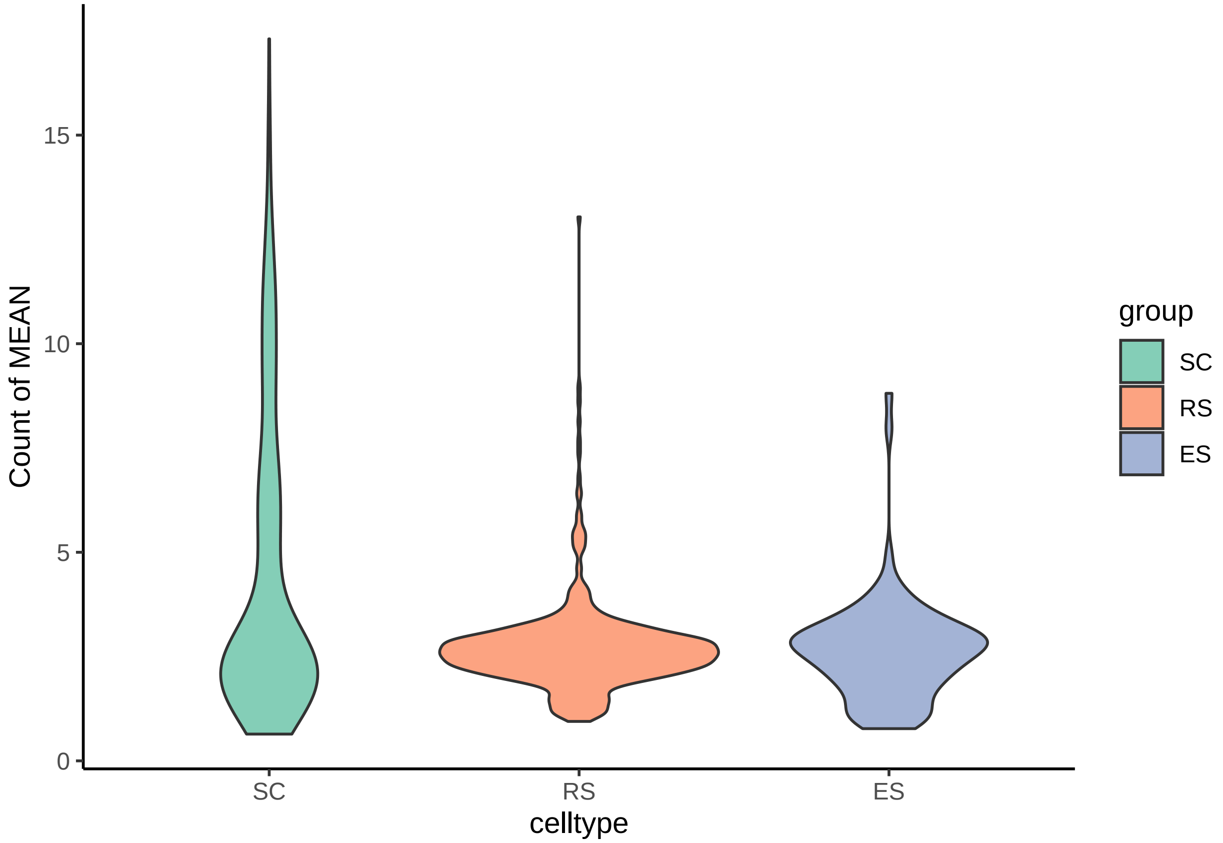

Modify plot by `statTheme`

We can modify the display of the figure by changing colors or other parameters, providing the `statTheme` parameter. Please see `?setStatTheme` for details about the parameters.

```
# display default statTHEME
# setStatTheme(NULL)

# To use another color palette
vizStats(scPACds, group='celltype', gene=gene, figType="violin",
         statTheme=list(group.cols=c(RColorBrewer::brewer.pal(8, "Set1"))))
```

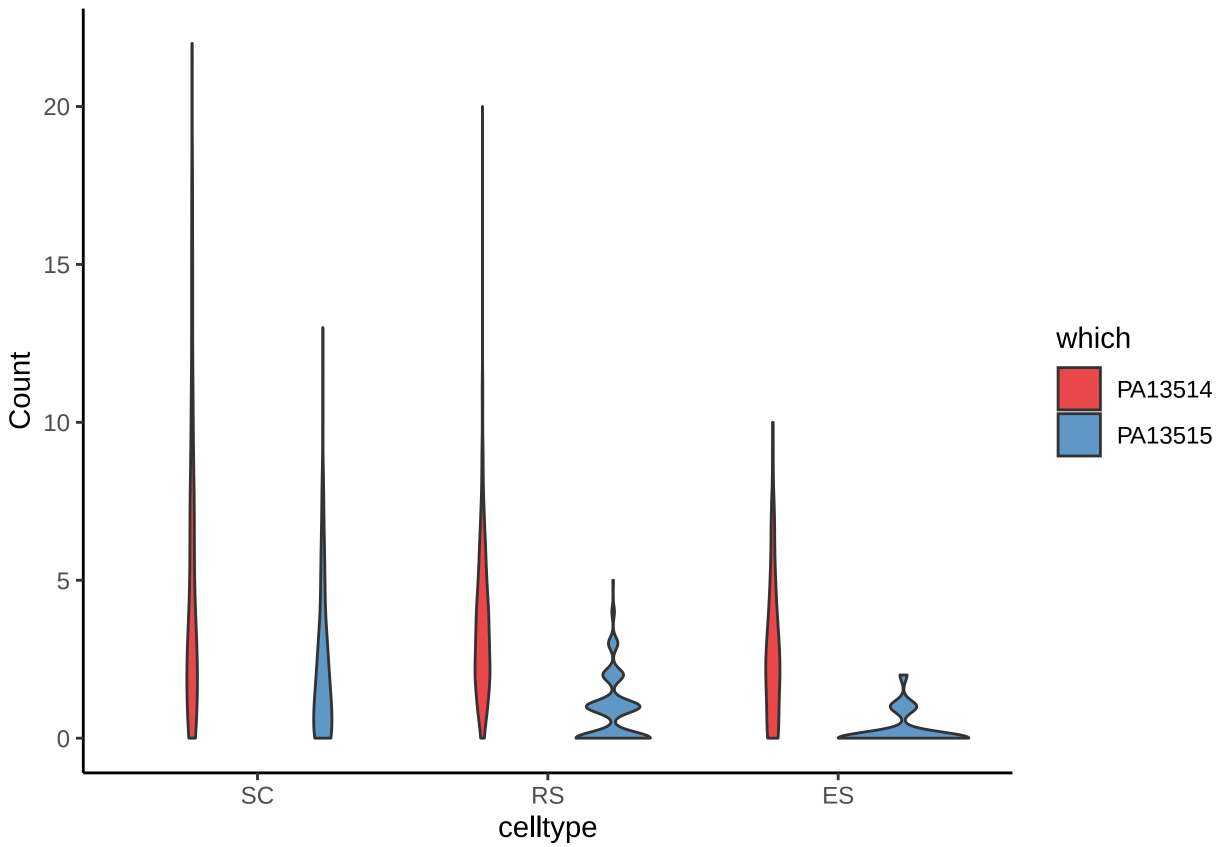

We can change the order of groups (e.g., cell types) by specifying `selGroups`.

```
# change the order to RS>SC>ES
vizStats(scPACds, group='celltype', selGroups=c('RS','SC','ES'))
```

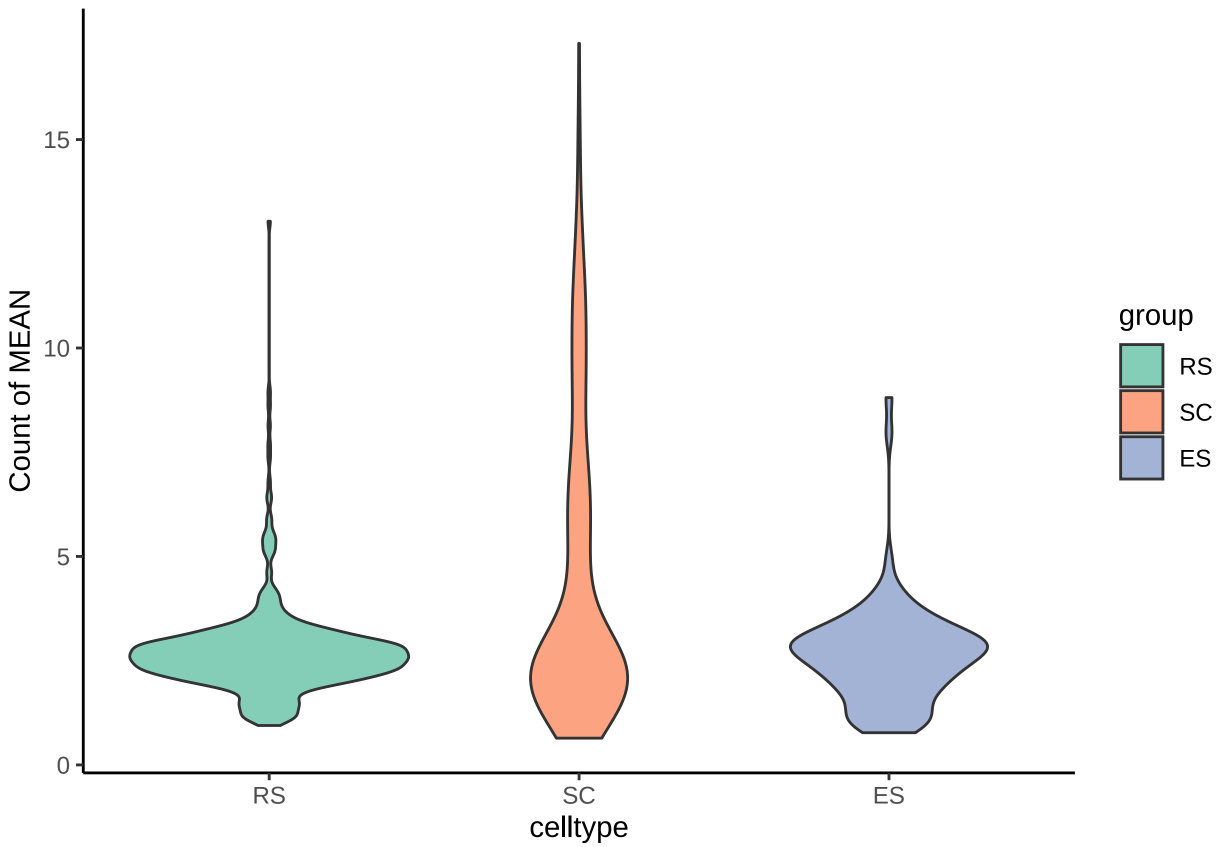

vizUMAP to plot 2D-embeddings

vizUMAP plots a UMAP plot where each point is a cell and it's positioned based on the cell embedding determined by the reduction technique.

The demo scPACds already contrains the coordinate labels of the 2D-embedding, UMAP_1 and UMAP_2.

```
colnames(scPACds@colData)
```

```
## [1] "orig.ident"      "nCount_RNA"      "nFeature_RNA"    "RNA_snn_res.0.5"
## [5] "seurat_clusters" "celltype"        "UMAP_1"          "UMAP_2"
## [9] "barcode"
```

UMAP plot for all genes

Here we plot the UMAP plot showing cell clusters and another UMAP plot overlaying with the mean expression value of pAs in each cell.

```
vizUMAP(scPACds, group='celltype', xcol='UMAP_1', ycol='UMAP_2')
```

```
## vizUMAP: group=celltype, x=UMAP_1, y=UMAP_2
```

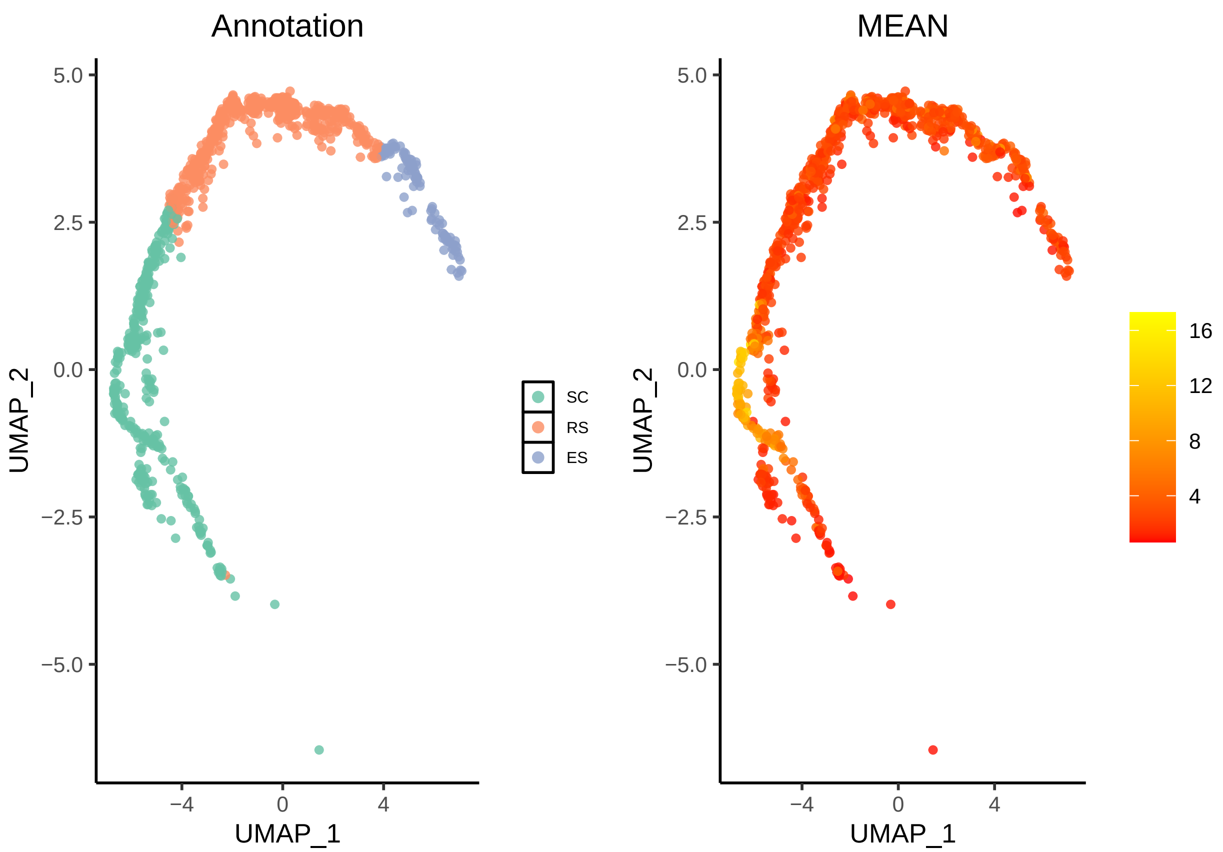


```r
# Plot only the overlaying UMAP
vizUMAP(scPACds, group='celltype', annoUMAP=FALSE, xcol='UMAP_1', ycol='UMAP_2')
```

```
## vizUMAP: group=celltype, x=UMAP_1, y=UMAP_2
```

```
## $MEAN
```

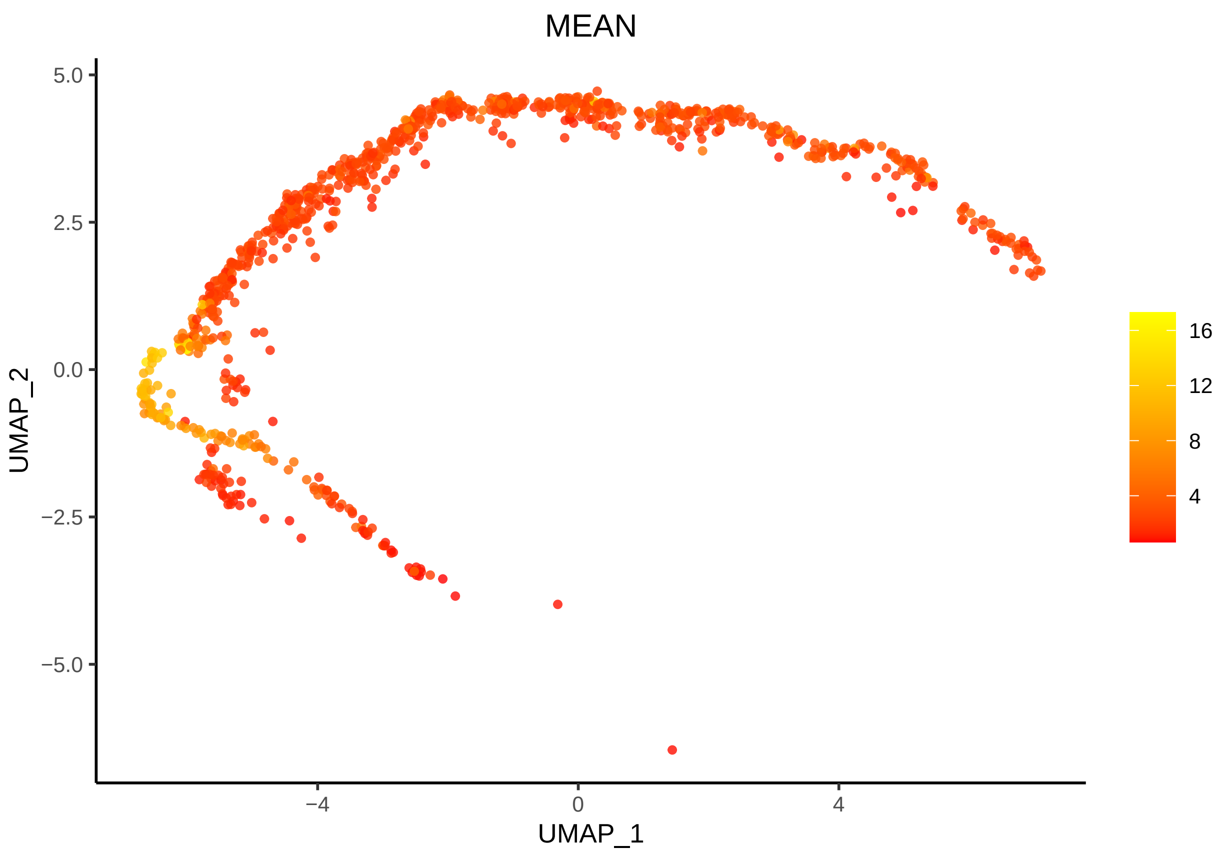

UMAP plot for given genes or pAs

Providing a gene id or a list of genes in the gene column of the `PACdataset`, we can plot a UMAP overlaying with the mean expression value of the gene(s).

```
vizUMAP(scPACds, group='celltype', xcol='UMAP_1', ycol='UMAP_2', genes=gene)
```

```
## vizUMAP: group=celltype, x=UMAP_1, y=UMAP_2
```

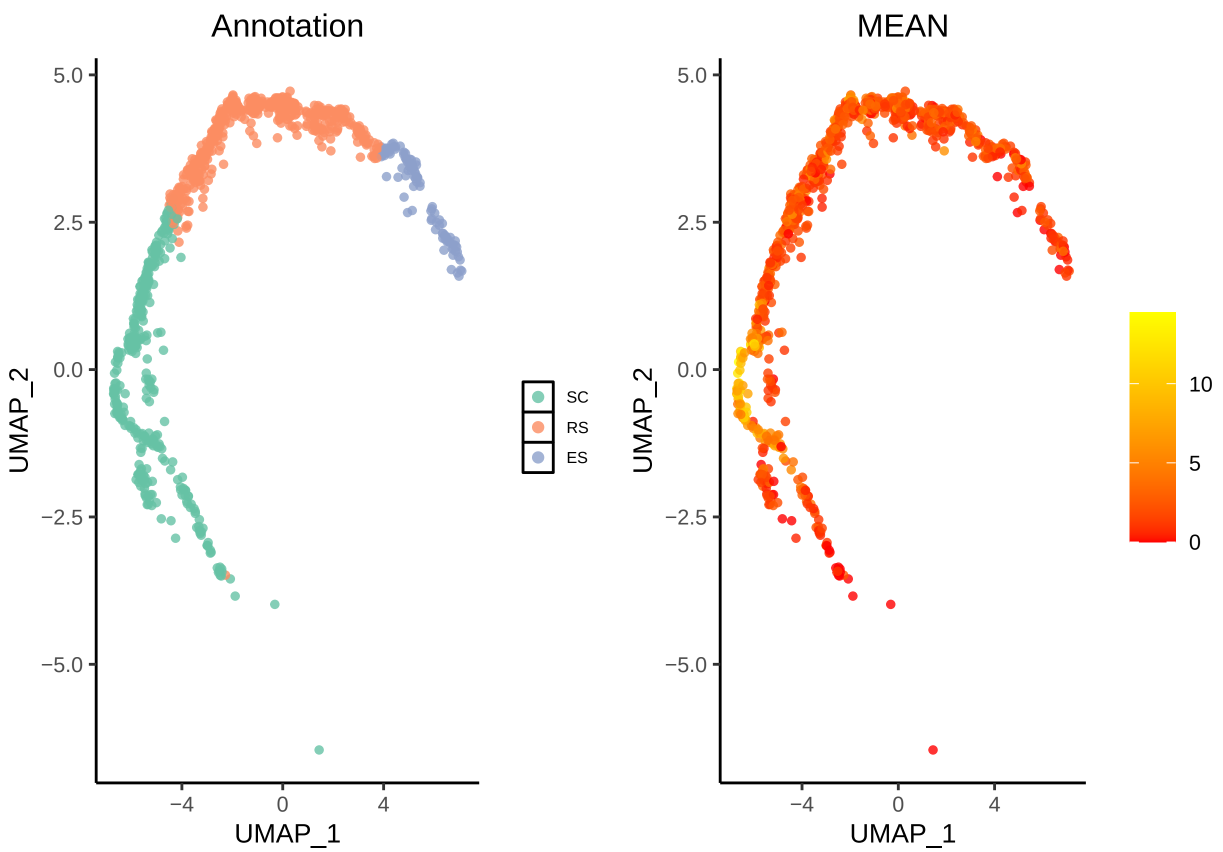

Similarly, we can provide ids of pAs corresponding to the rownames in the `PACdataset` instead of genes.

```
# here we only show two cell types by specifying selGroup
vizUMAP(scPACds, group='celltype', xcol='UMAP_1', ycol='UMAP_2',
        selGroups=c('SC','RS'), PAs=PAids)
```

```
## vizUMAP: group=celltype, x=UMAP_1, y=UMAP_2
```

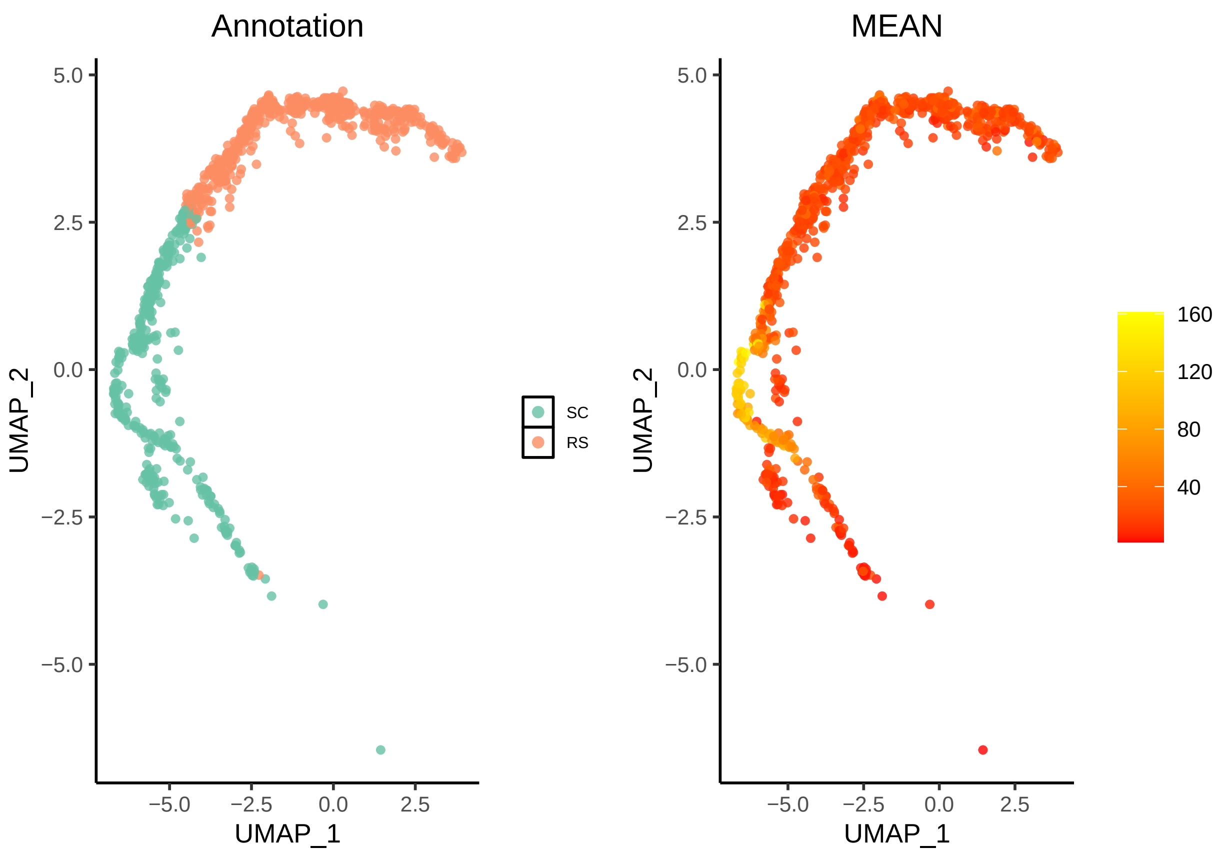

It is also possible to get embeddings based on the counts data in the `PACdataset`, using the `Seurat` package.

```
## get embeddings using the pA count matrix with normalization
scPACds=reduceDim(scPACds, dims=1:10, dimLabel='umap_norm', norm=TRUE)
```

```
## Normalize data by LogNormalize...
## Find variable features...
## Scale data...
## Run PCA...
## Run UMAP...
```

```
## without normalization
scPACds=reduceDim(scPACds, dims=1:10, dimLabel='umap_raw', norm=FALSE)
```

```
## Find variable features...
## Scale data...
## Run PCA...
## Run UMAP...
```

```
## There are new columns adding to the colData slot.
head(scPACds@colData)
```

```
##                  orig.ident nCount_RNA nFeature_RNA RNA_snn_res.0.5
## AAACCTGAGCTTATCG       gene      23617         5061               9
```

```
## AAACCTGGTTGAGTTC         gene      19555          4802               9
## AAACCTGTCAACGAAA         gene      23467          5009               8
## AAACGGGCACAGGTTT         gene      28832          5484               8
## AAACGGGTCATTTGGG         gene      18931          4819               8
## AAACGGGTCCTCATTA         gene      15734          3855               8
##                    seurat_clusters celltype       UMAP_1      UMAP_2
## AAACCTGAGCTTATCG                 9       RS   0.361751856 4.528803031
## AAACCTGGTTGAGTTC                 9       RS  -0.119255482 4.563224952
## AAACCTGTCAACGAAA                 8       RS   3.023034156 4.074635188
## AAACGGGCACAGGTTT                 8       RS   3.322863163  3.81046788
## AAACGGGTCATTTGGG                 8       ES    4.73071772 3.419416826
## AAACGGGTCCTCATTA                 8       ES   5.306060375 3.274766843
##                            barcode umap_norm1  umap_norm2 umap_raw1 umap_raw2
## AAACCTGAGCTTATCG AAACCTGAGCTTATCG  -5.620694 -2.46420018 -2.987978  3.323227
## AAACCTGGTTGAGTTC AAACCTGGTTGAGTTC  -4.992321 -2.64142004 -2.718818  1.115900
## AAACCTGTCAACGAAA AAACCTGTCAACGAAA  -8.836434 -0.09717218 -4.899041  3.001630
## AAACGGGCACAGGTTT AAACGGGCACAGGTTT  -9.173502 -0.23153750 -4.818650  4.609036
## AAACGGGTCATTTGGG AAACGGGTCATTTGGG  -9.307289  2.44096967 -6.714005  2.333385
## AAACGGGTCCTCATTA AAACGGGTCCTCATTA  -9.853778  2.75157370 -6.933767  1.390677
```

We can use the new 2D-embeddings to plot UMAP.

```
vizUMAP(scPACds, group='celltype', xcol='umap_raw1', ycol='umap_raw2')
```

```
## vizUMAP: group=celltype, x=umap_raw1, y=umap_raw2
```

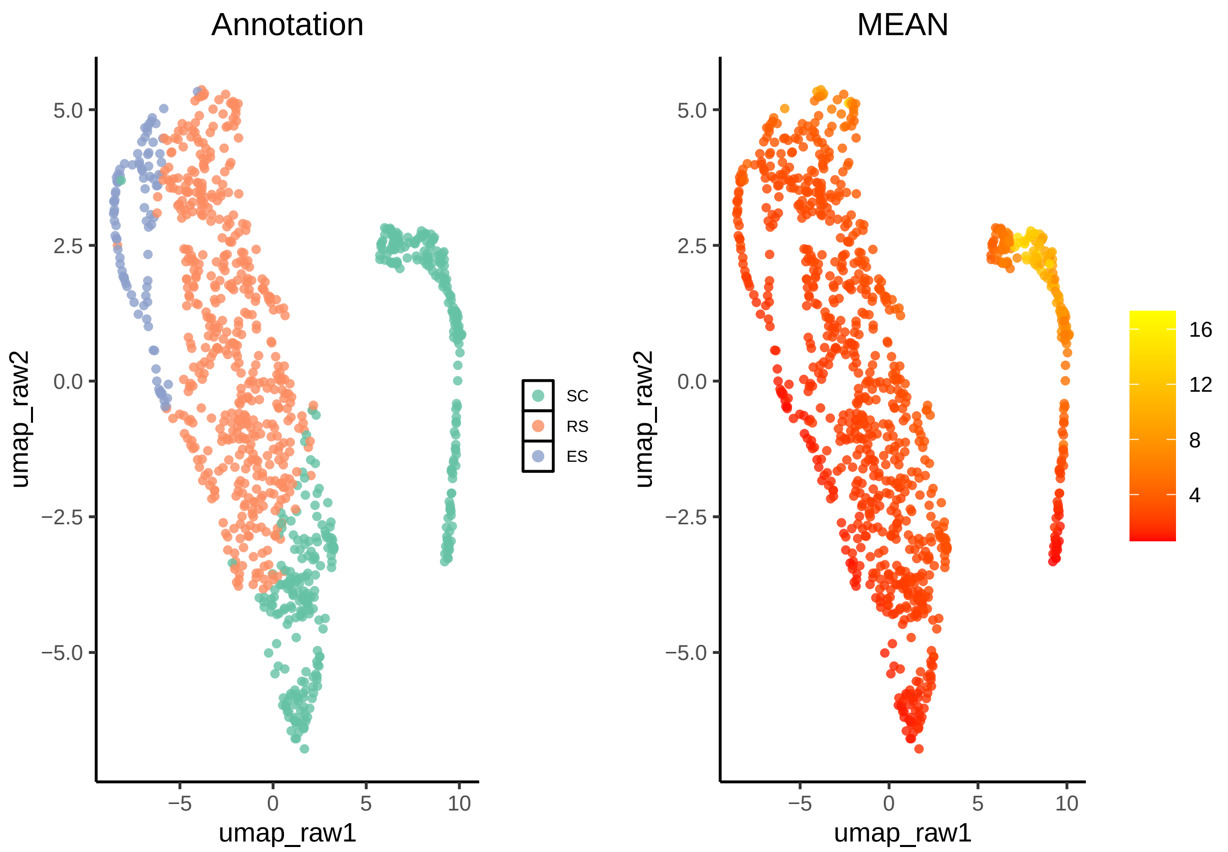

```r
vizUMAP(scPACds, group='celltype', xcol='umap_norm1', ycol='umap_norm2')
```

```
## vizUMAP: group=celltype, x=umap_norm1, y=umap_norm2
```

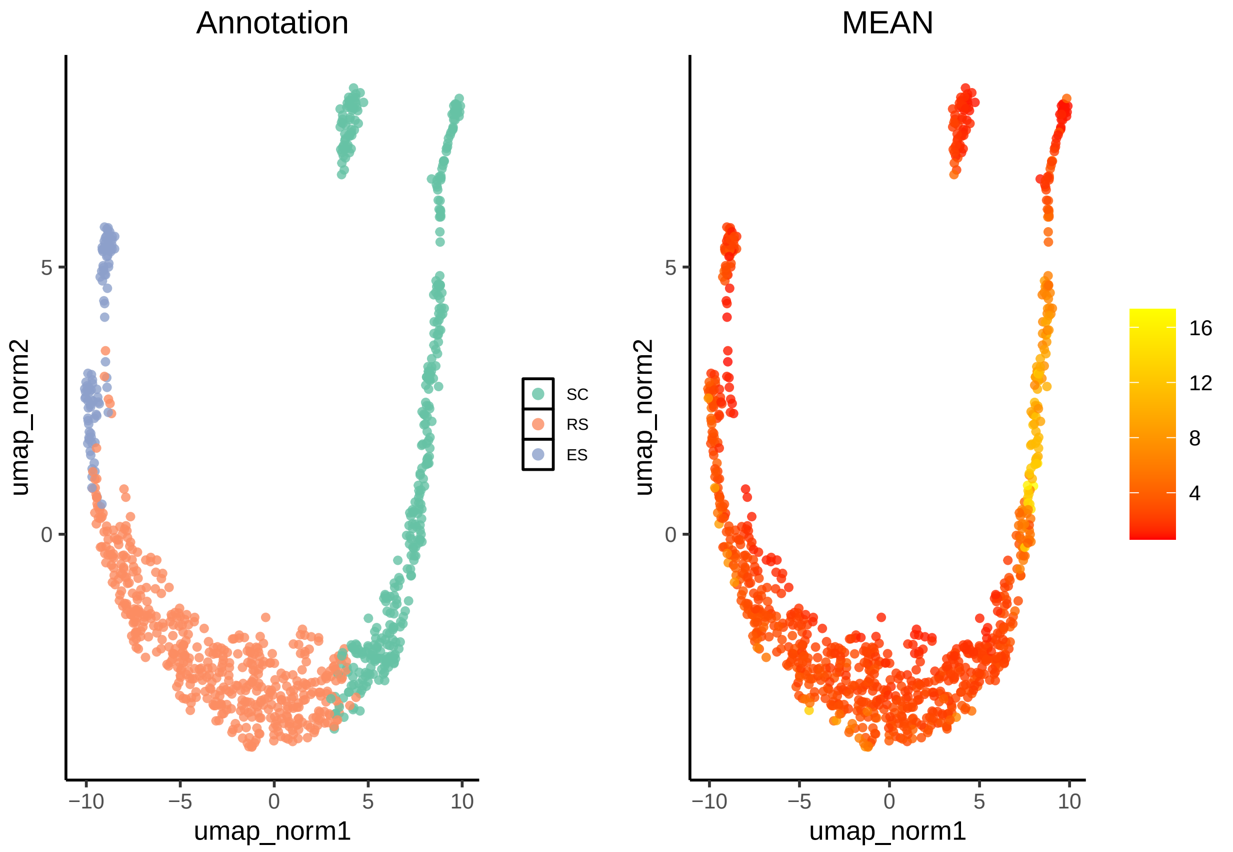

vizAPAmarkers to visualize APA markers across cell categories

An APA marker is a APA gene with differential APA usage between two pAs in the 3'UTR of the gene. Here we calculate the relative usage of distal pA (RUD) to represent the APA usage of each gene. A larger RUD means the longer 3'UTR.

Get APA markers by RUD index

`getAPAindexPACds` utilizes `movAPA::movAPAindex` to calculate the RUD index for each 3'UTR-APA gene in a `PACdataset` and returns a PACdataset. This function only implements the RUD index in movAPA, users can use `movAPA::movAPAindex` for more types of APA index.

```r
# First, calculate the RUD index for each gene.
# Only genes with 3'UTR APA can be used for RUD calculation.
iPACds=getAPAindexPACds(scPACds, choose2PA='PD')
```

Then we can obtain APA markers by wilcox.test for each pair of cell types. Actually, we can use any other tools to obtain the marker list, as long as a gene list is obtained.

```r
m=getAPAmarkers(iPACds,  group='celltype', everyPair = TRUE)
```

```
## PACds row = gene, PACds dataType = ratio
## It seems that PACds is APA ratio,
## will apply FindMarkers (default is wilcox-test) on the APA index
## to get DE APA events (each row is an APA gene).
```

Next, we can count the number of APA markers.

```r
countAPAmarkers(m)
```

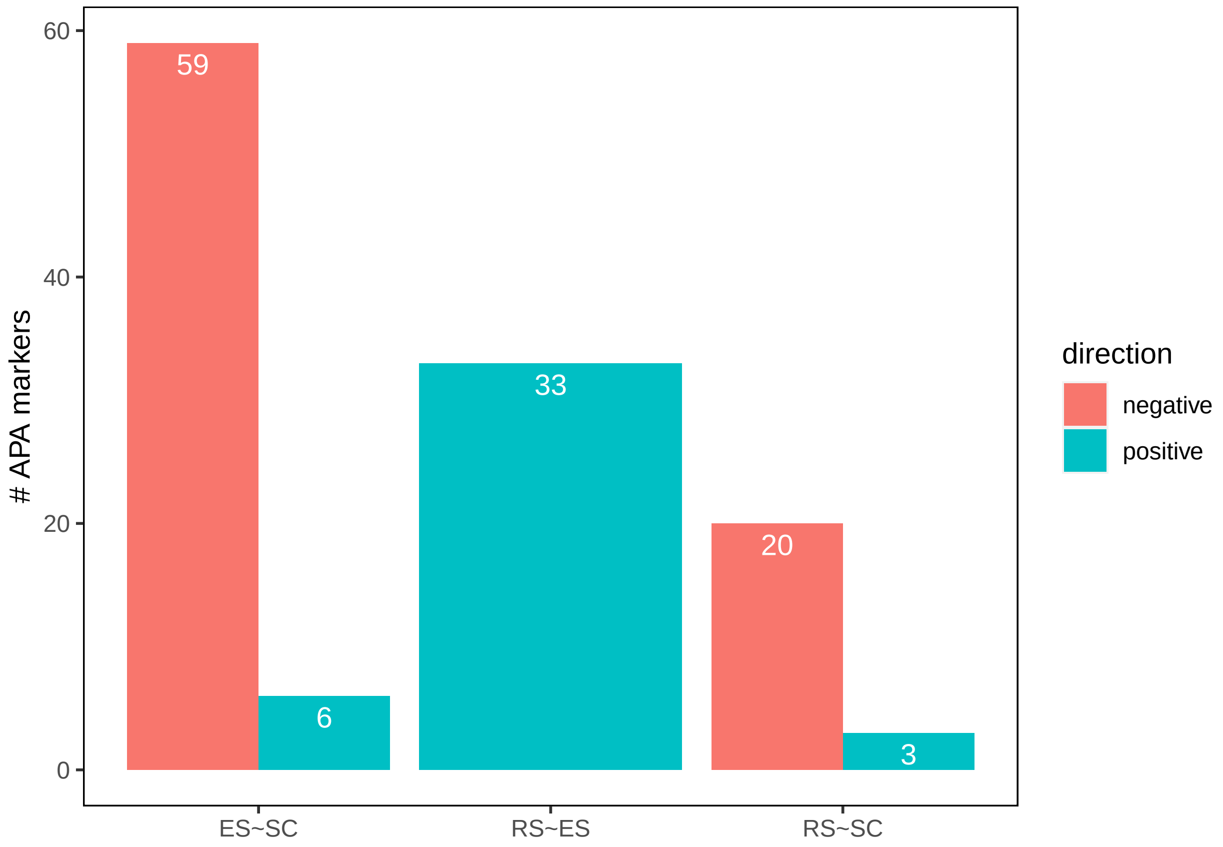


```
## # A tibble: 5 x 3
##   pair  direction Count
##   <chr> <chr>     <int>
## 1 ES~SC negative     59
## 2 ES~SC positive      6
## 3 RS~ES positive     33
## 4 RS~SC negative     20
## 5 RS~SC positive      3
```

Plot APA markers

Having a list of APA markers (i.e., gene ids), it is easy to visualize them by `vizAPAMarkers`.

```
# Visualize the top 6 APA markers, showing all the three cell types
vizAPAMarkers(iPACds, group='celltype',
              markers=m$rowid[1:6],
              figType = 'violin')
```

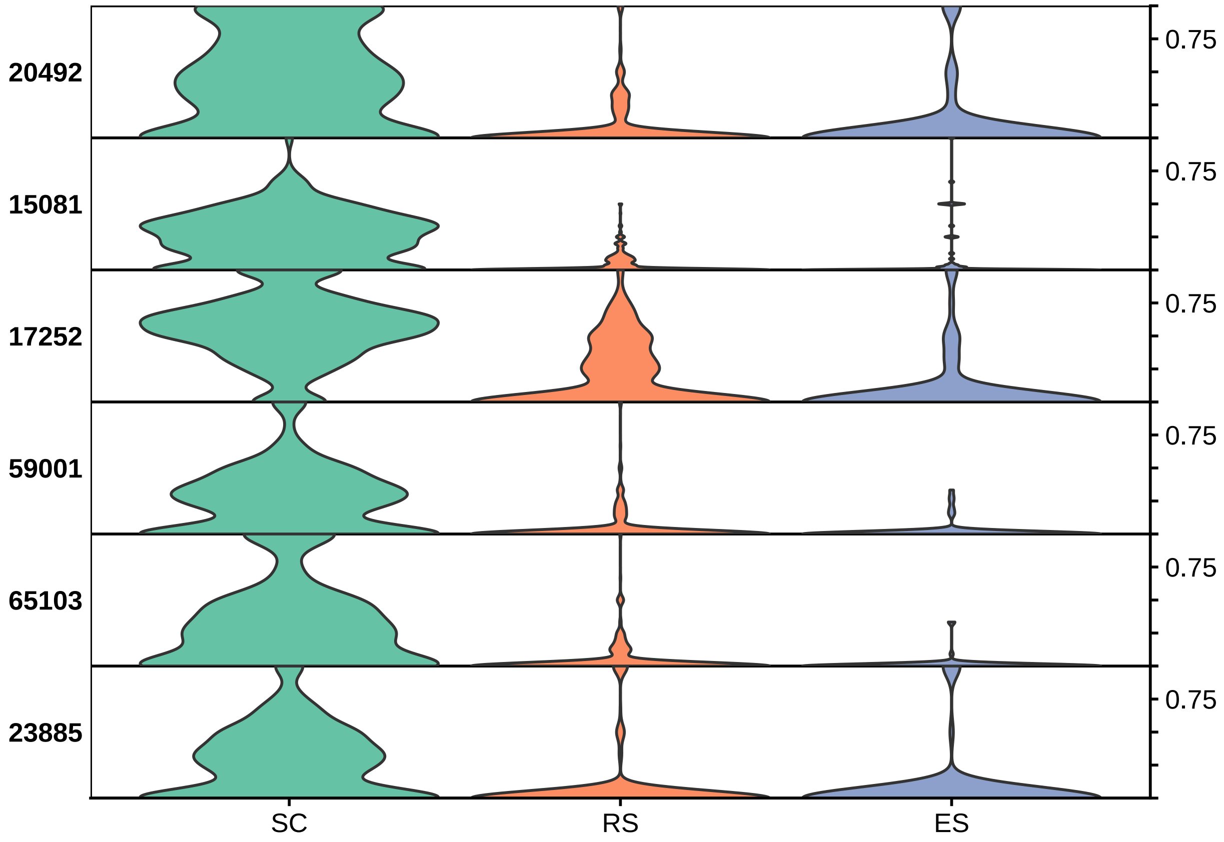

It is easy to plot many other kinds of plots for visualize APA markers.

```
# Actually the top 6 markers are between ES and SC,
# so we can only show these two cell types
vizAPAMarkers(iPACds, group='celltype', markers=m$rowid[1:6],
              selGroups=c('ES','SC'), figType = 'violin')
```

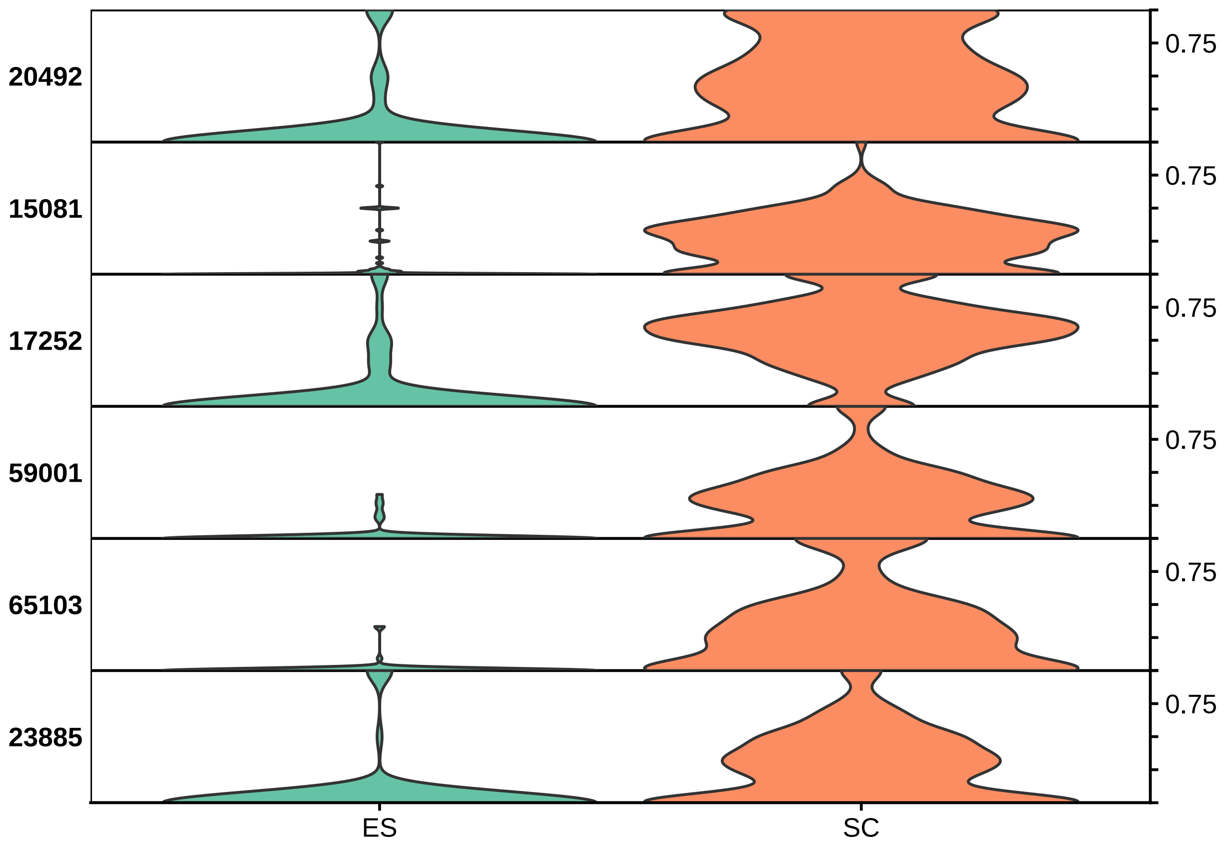


```
# To show genes as columns and cell types as rows
vizAPAMarkers(iPACds, group='celltype', markers=m$rowid[1:6],
              figType = 'violin', statTheme=list(xgroup=FALSE))
```

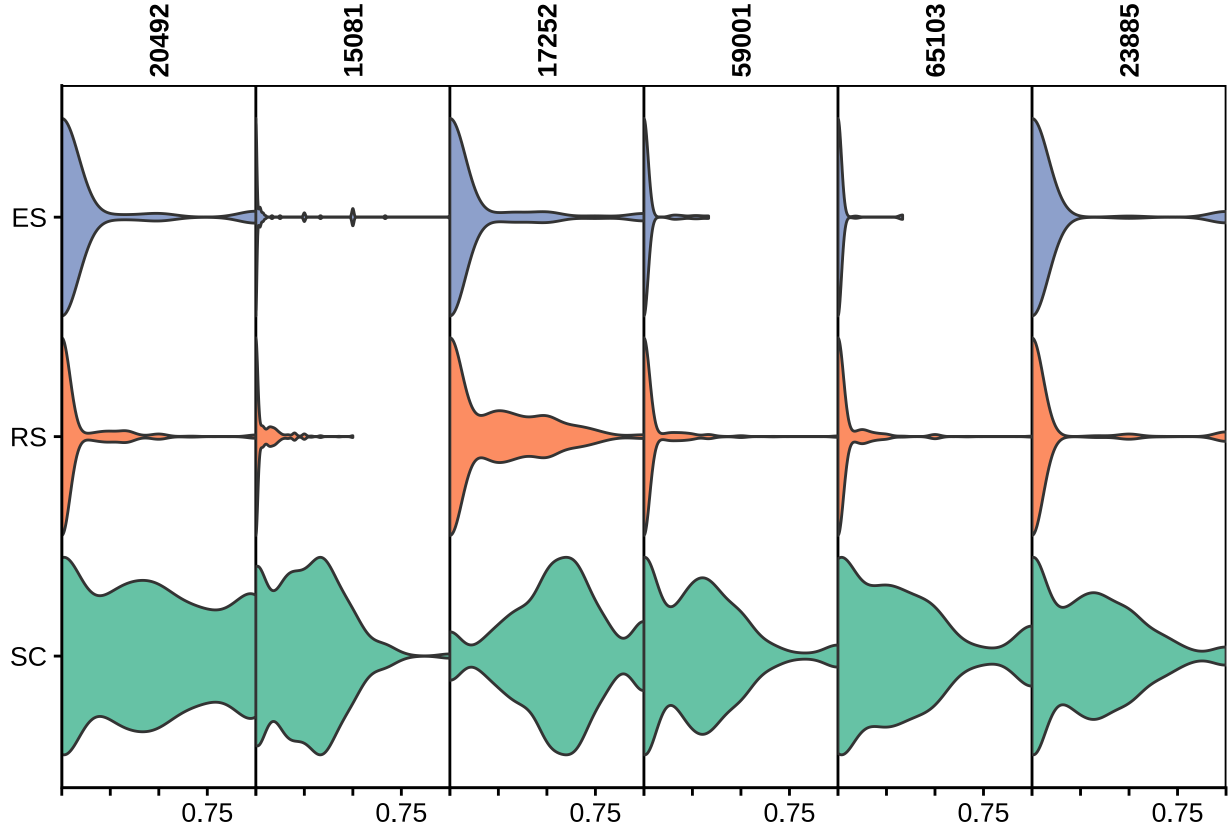


```
# Plot a heatmap for APA markers
vizAPAMarkers(iPACds, group='celltype', markers=m$rowid[1:6],
              figType = 'heatmap')
```

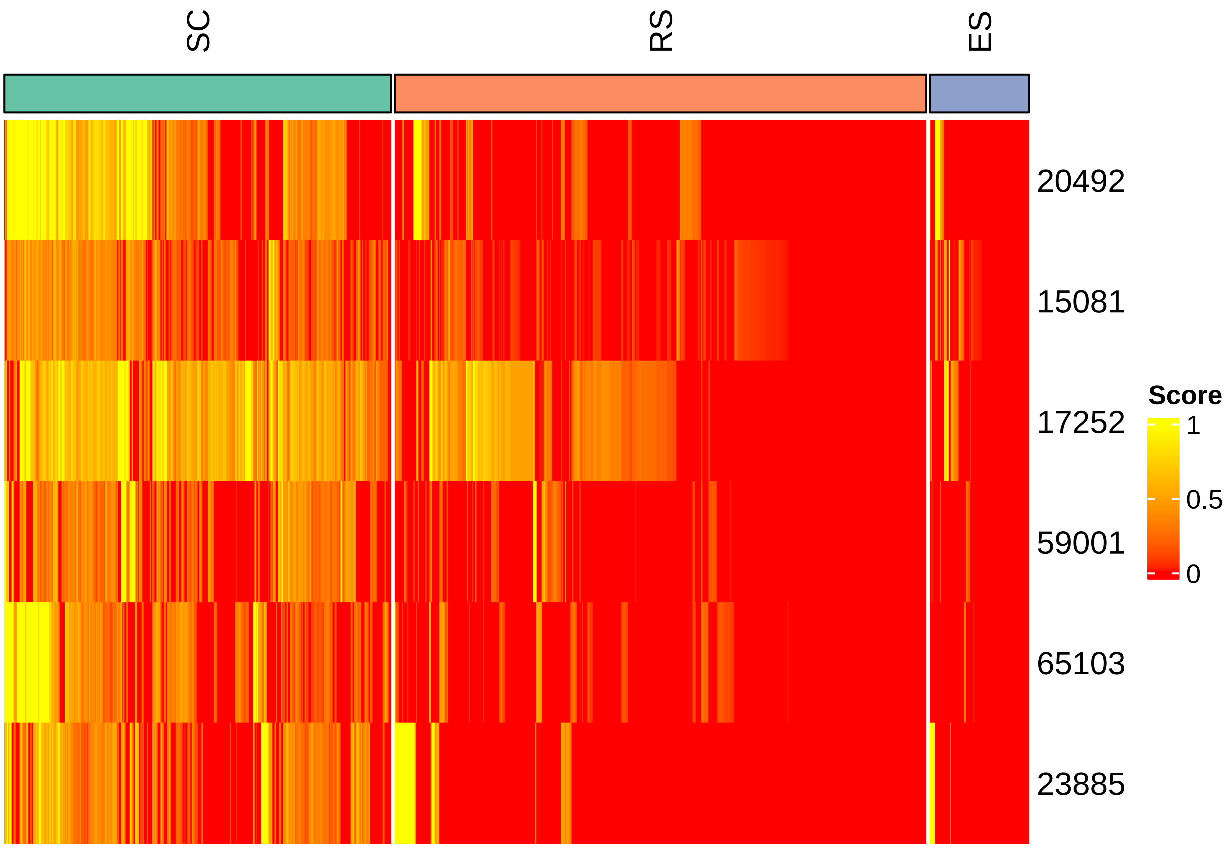


```
# Plot a heatmap and cluster markers and show dendrograms
vizAPAMarkers(iPACds, group='celltype', markers=m$rowid[1:6],
              figType = 'heatmap',
              statTheme=list(hm.cluster.markers=TRUE, hm.cluster.dend=TRUE))
```

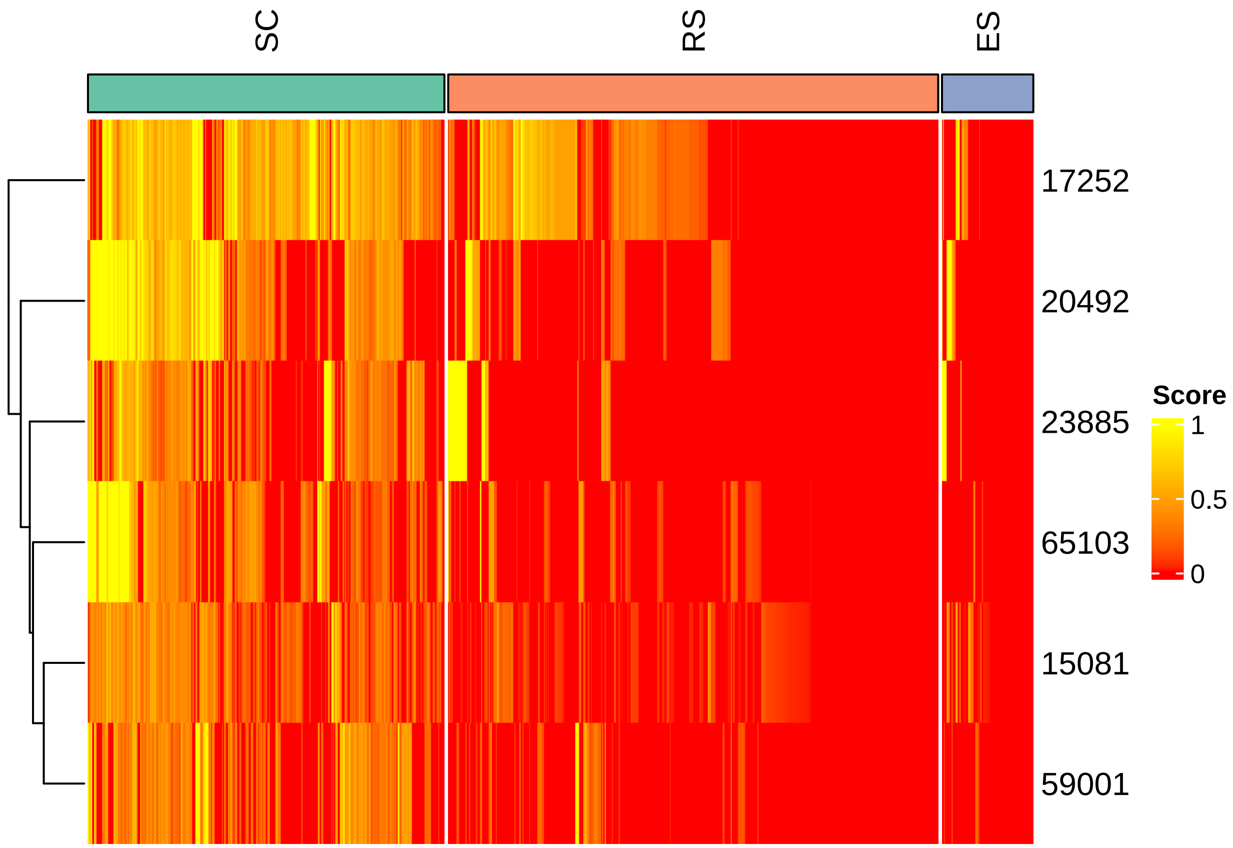


```
# Plot a bubble plot for markers
vizAPAMarkers(iPACds, group='celltype', markers=m$rowid[1:6],
              figType = 'bubble')
```

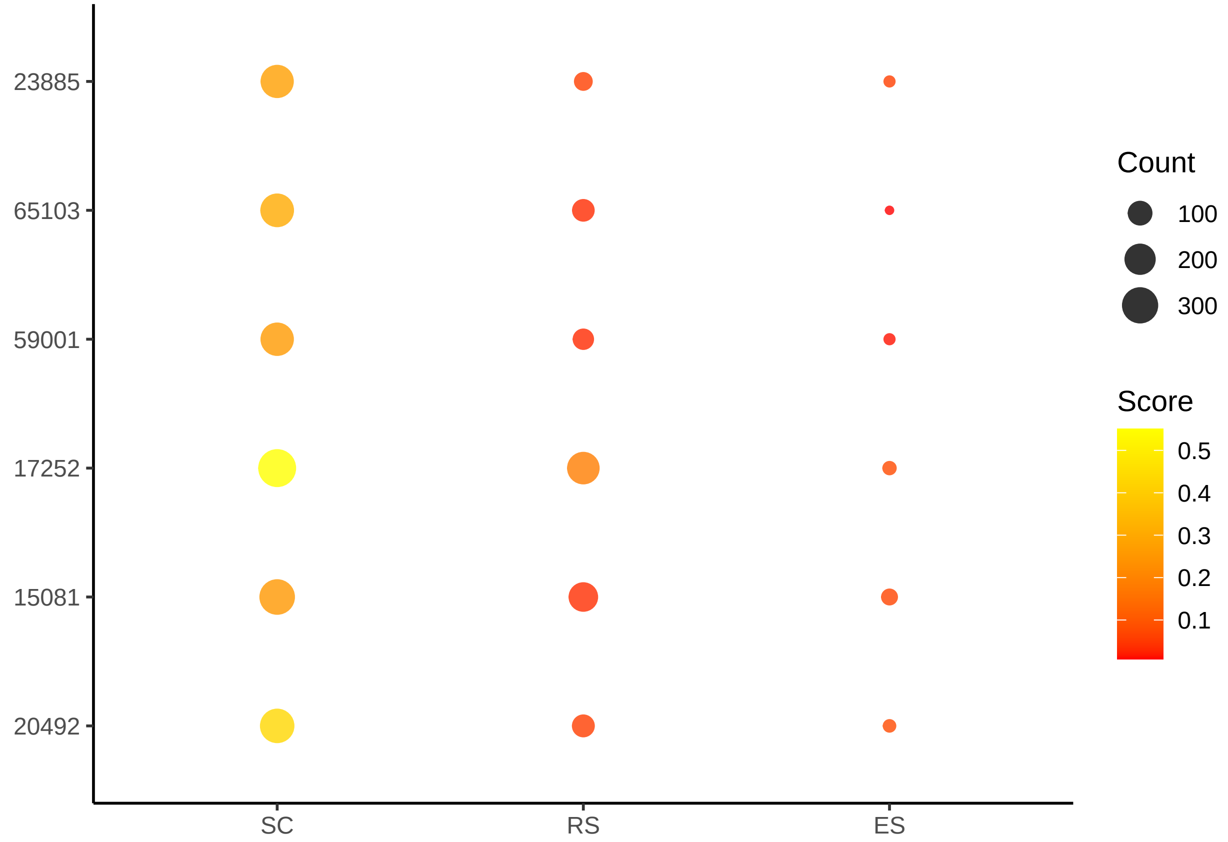


```r
# Switch the x/y axis of the bubble plot
vizAPAMarkers(iPACds, group='celltype', markers=m$rowid[1:6],
              figType = 'bubble', statTheme=list(xgroup=FALSE))
```

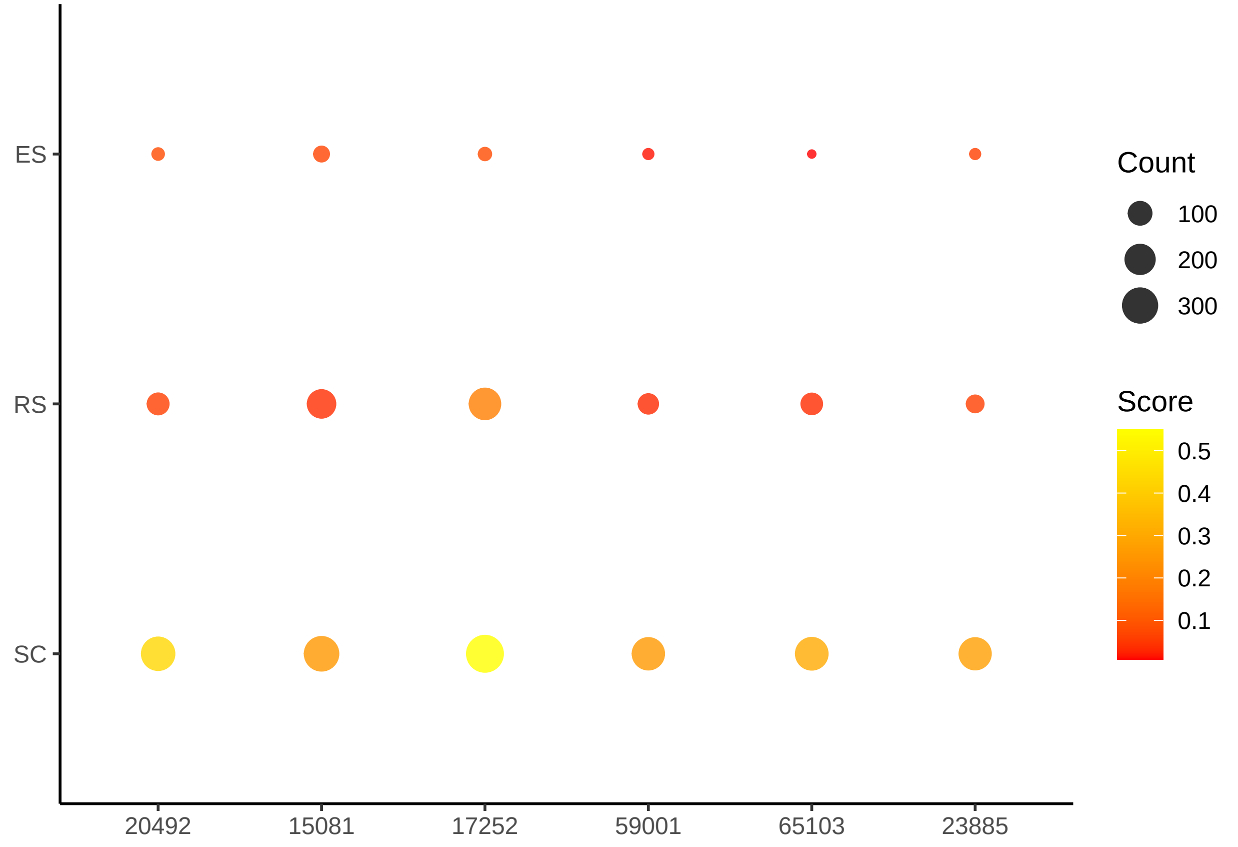

UMAP plot for APA markers

Set `figType='umap'` would plot UMAP plot for the given APA maker(s).

```
# Plot the UMAP plot
vizAPAMarkers(iPACds, group='celltype', markers=m$rowid[1:6],
              figType="umap", umap.x='UMAP_1', umap.y='UMAP_2')
```

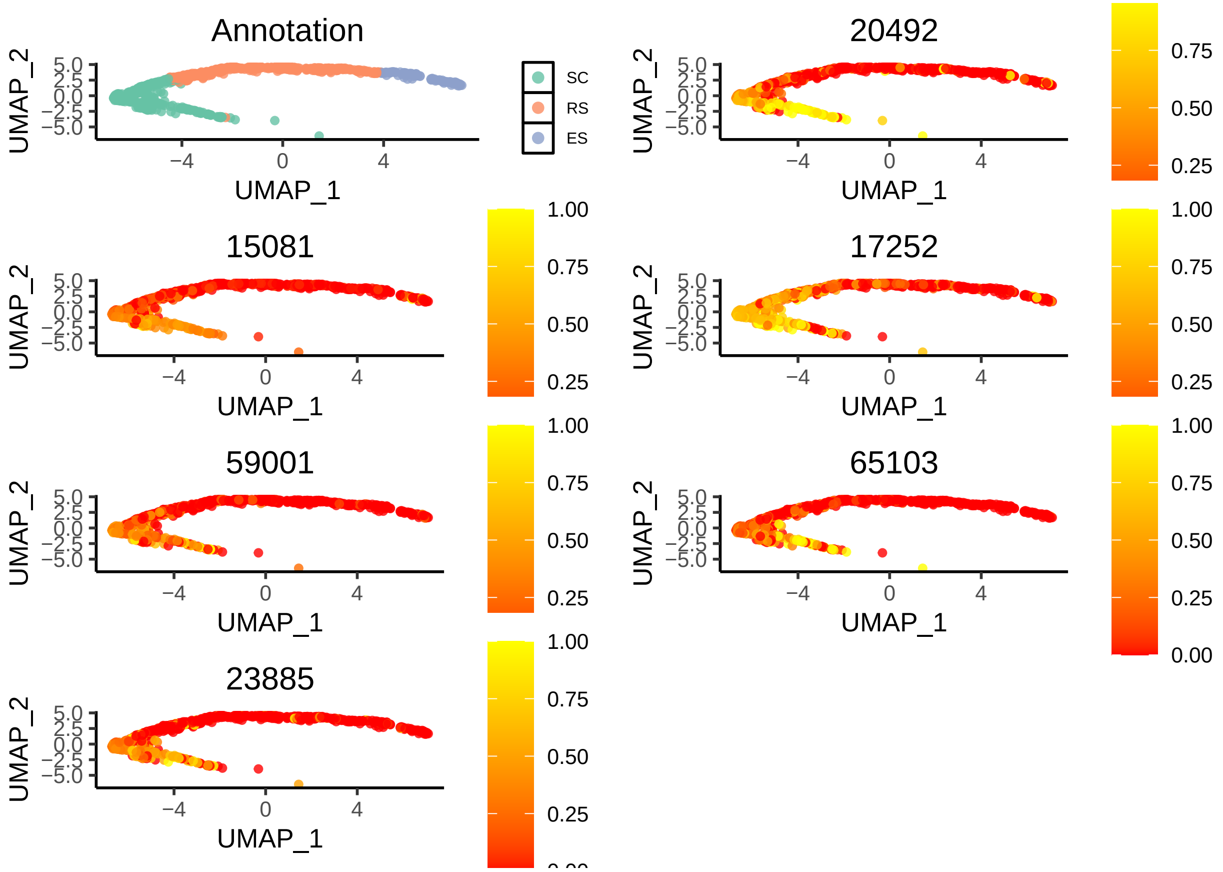


```r
# Do not show the UMAP embeddings
vizAPAMarkers(iPACds, group='celltype', markers=m$rowid[1:6],
              figType="umap", umap.x='UMAP_1', umap.y='UMAP_2', annoUMAP=F)
```

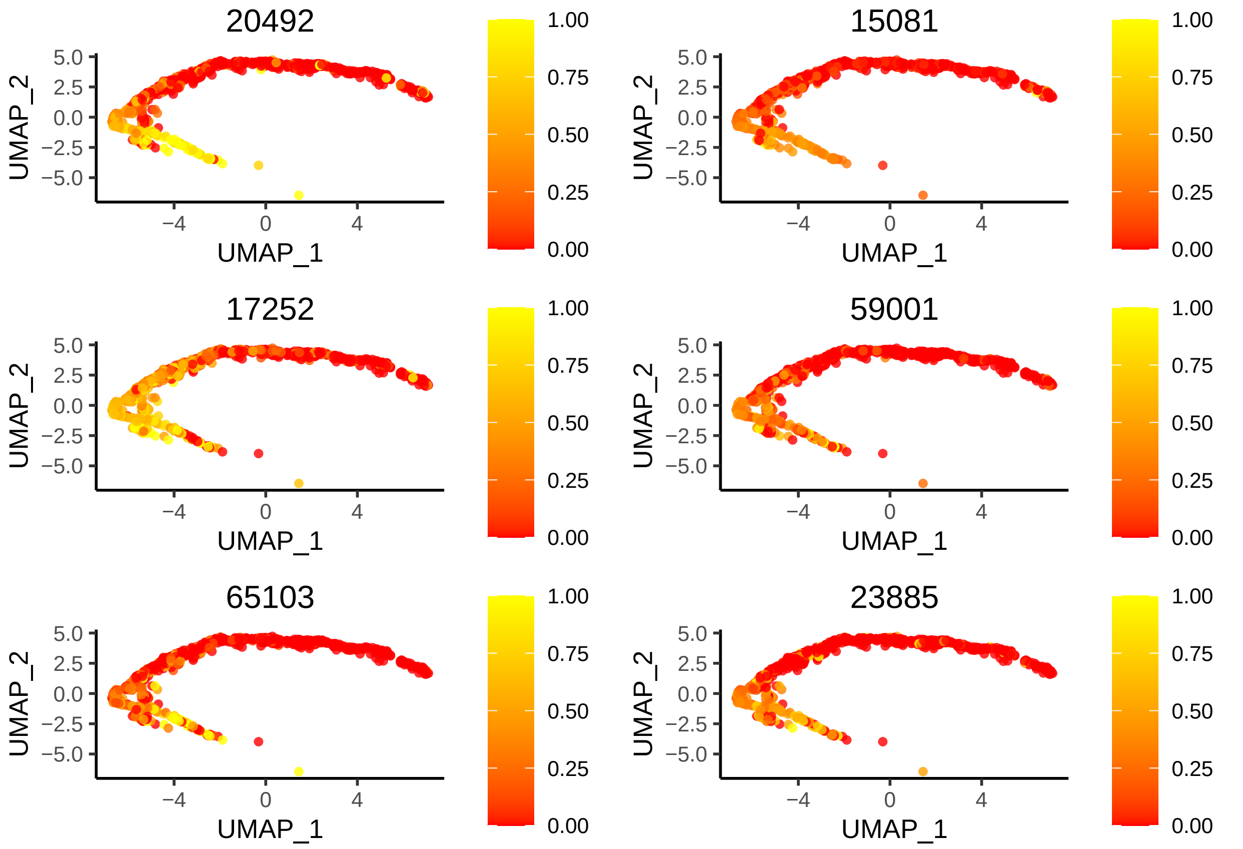

Get APA markers based on other APA index

Here we use movAPA to calculate another APA index called smartRUD, which is a more robust index than RUD. First proximal and distal pAs are chosen by `get3UTRAPApd` in a smarter way. Then the index can be obtained, and converted to PACdataset format.

```
# first, get smartRUD APA index by movAPA
pd=movAPA::get3UTRAPApd(pacds=scPACds,
                        minDist=50, maxDist=5000,
                        minRatio=0.05, fixDistal=FALSE,
                        addCols='pd')
```

```
## get3UTRAPApd: filtering by minRatio: gene# before: 413 ; after: 172 ; remove: 241
## get3UTRAPApd: filtering pd (dist between): gene# before: 172 ; after: 149 ; remove: 23
## get3UTRAPApd: add four columns to pacds@anno: pdWhich, pdScore, pdRatio, pdDist
```

```
srud=movAPA::movAPAindex(pd, method="smartRUD", sRUD.oweight=FALSE)
head(srud[, 1:10])
```

```
## 6 x 10 Matrix of class "dgeMatrix"
##        AAACCTGAGCTTATCG AAACCTGGTTGAGTTC AAACCTGTCAACGAAA AAACGGGCACAGGTTT
## 106369        0.0000000        0.3750000        0.2500000        0.2222222
## 107566        0.8181818        0.8571429        0.2500000        0.2222222
## 109232        1.0000000        0.8461538        0.9000000        0.9090909
```

```
## 11421          0.0000000        0.2500000        0.0000000        0.0000000
## 114641         0.0000000        0.0000000        0.0000000        0.0000000
## 12226          1.0000000        1.0000000        0.4545455        0.4210526
##        AAACGGGTCATTTGGG AAACGGGTCCTCATTA AAAGATGAGGTACTCT AAAGCAAAGACAGACC
## 106369       0.00000000        0.2000000       0.09090909       0.38235294
## 107566       0.20000000        0.1764706       1.00000000       0.95348837
## 109232       0.87500000        1.0000000       0.92857143       0.91139241
## 11421        0.00000000        0.0000000       0.00000000       0.00000000
## 114641       0.00000000        0.0000000       0.00000000       0.07142857
## 12226        0.03846154        0.1666667       1.00000000       1.00000000
##        AAAGCAAAGGTACTCT AAAGCAACAAAGTCAA
## 106369       0.05000000        0.1250000
## 107566       0.09433962        1.0000000
## 109232       0.85714286        0.9333333
## 11421        0.00000000              NaN
## 114641       0.16666667        0.0000000
## 12226        0.26666667        1.0000000
```

```
# convert to PACds
iPACds=APAindex2PACds(srud, colData=pd@colData)

# get APA markers
m=getAPAmarkers(iPACds,  group='celltype', everyPair = TRUE)
```

```
## PACds row = gene, PACds dataType = ratio
## It seems that PACds is APA ratio,
## will apply FindMarkers (default is wilcox-test) on the APA index
## to get DE APA events (each row is an APA gene).
```

```
# visualize top APA markers
vizAPAMarkers(iPACds, group='celltype',
              markers=m$rowid[1:6],
              figType = 'violin')
```

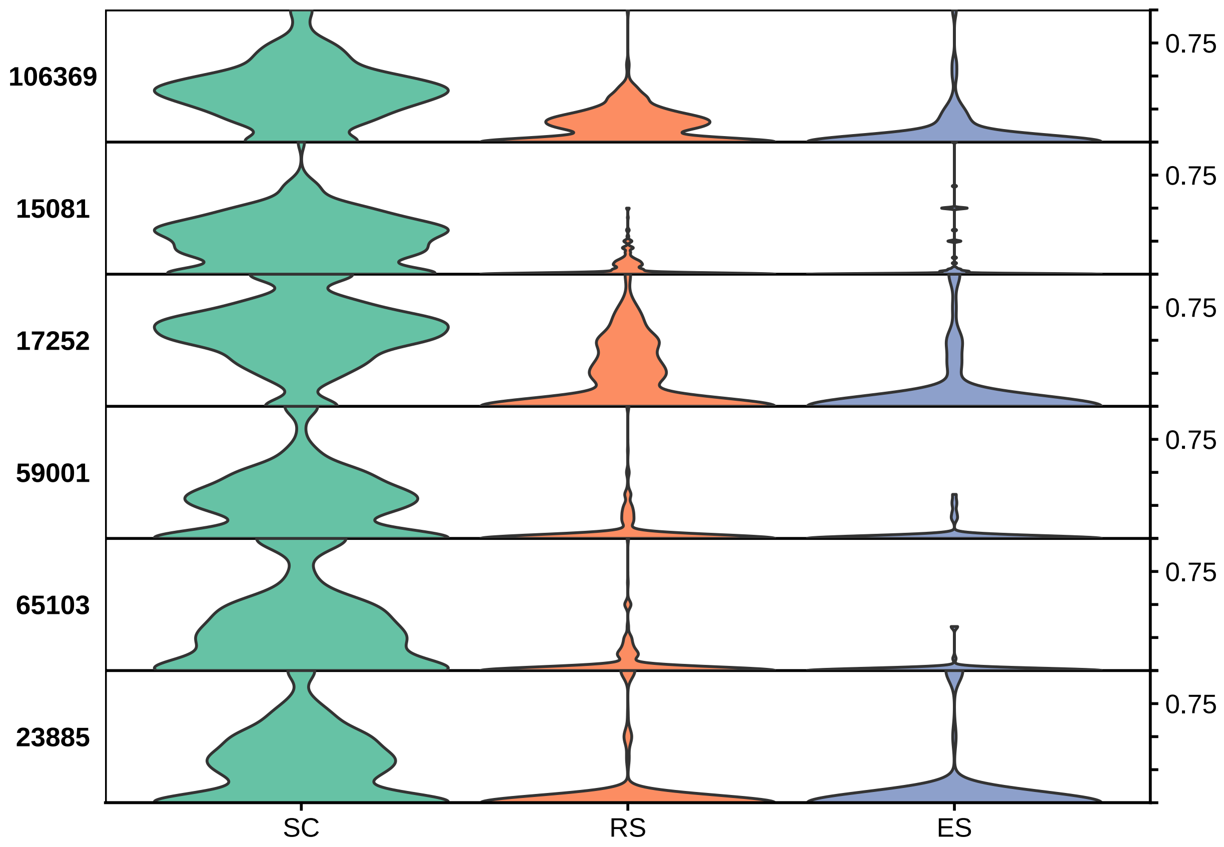

Get APA markers by read counts

We recommend to convert the pA expression matrix to APA index matrix for detecting APA markers. However, it is also possible to detect differential expression for each pA using only the pA counts.

```
# The PACds is a PA-count dataset,
# in which each row is the read count for each pA.
# Here we detect differential expression for each pA as markers.
m=getAPAmarkers(scPACds,  group='celltype', everyPair = TRUE)
```

```
## PACds row = PA, PACds dataType = count
## Warning: it seems that PACds is pA count, will get DE pAs (each row is a pA).
## If you want APA markers, please use getAPAindexPACds() to transform your PACds to APA ratio.
```

```
head(m)
```

```
##              p_val avg_log2FC pct.1 pct.2     p_val_adj cluster1 cluster2
## 462 4.622163e-132   3.015683 0.992 0.366 4.501987e-129       RS       SC
## 463 1.693718e-129   2.951508 0.978 0.259 1.649681e-126       RS       SC
## 464 9.601512e-126   2.543974 0.990 0.427 9.351873e-123       RS       SC
## 465 1.504412e-125   3.249244 0.996 0.590 1.465297e-122       RS       SC
## 466 6.822939e-125   2.599823 0.982 0.325 6.645543e-122       RS       SC
## 467 6.835973e-121   2.099977 0.950 0.176 6.658238e-118       RS       SC
##       rowid direction
```

```
## 462  PA1392  positive
## 463 PA17947  positive
## 464  PA4365  positive
## 465  PA1591  positive
## 466 PA14630  positive
## 467  PA4971  positive
```

```
# Show the read count difference of the top 10 markers
vizAPAMarkers(scPACds, group='celltype',
              markers=m$rowid[1:10],
              figType="violin")
```

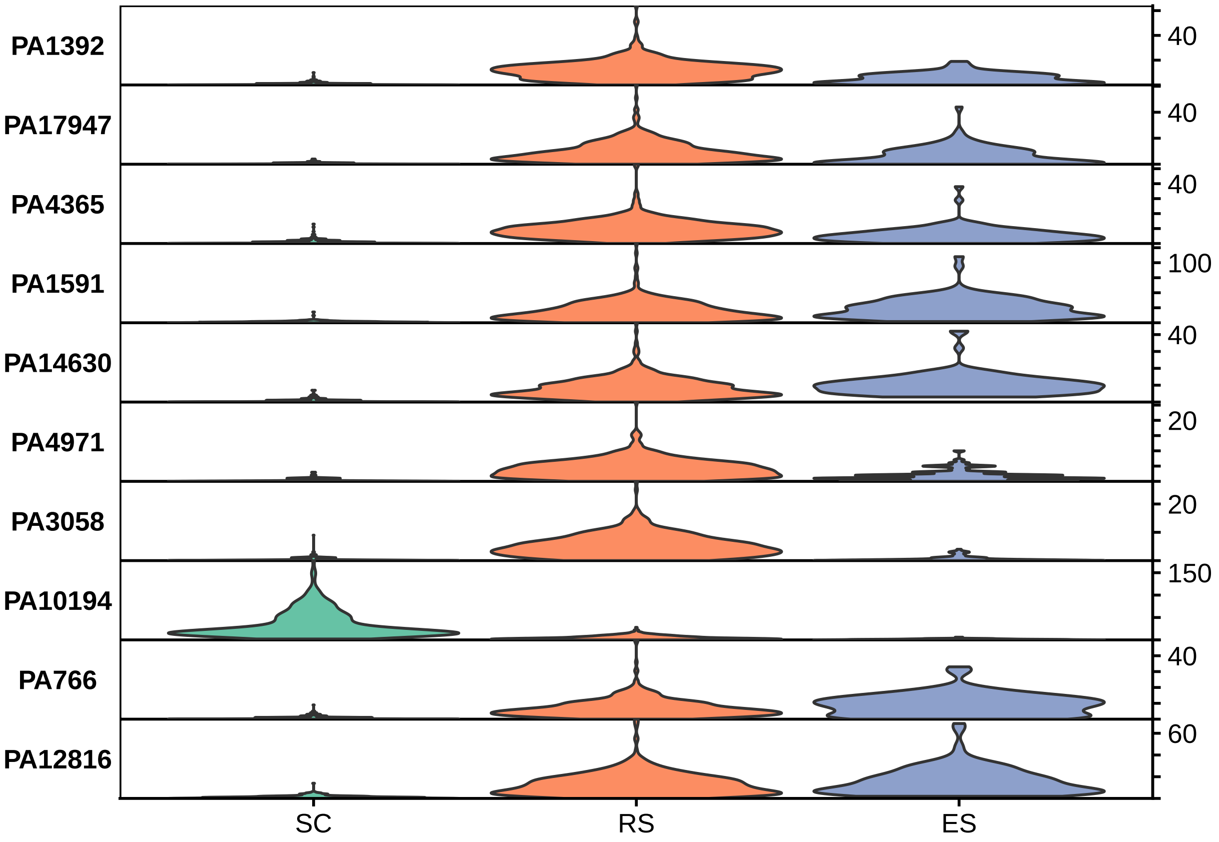


```
# Switch the x-y axis to show pA in columns and cell type in rows.
vizAPAMarkers(scPACds, group='celltype',
              markers=m$rowid[1:10],
              figType="violin",
              statTheme=list(xgroup=FALSE))
```

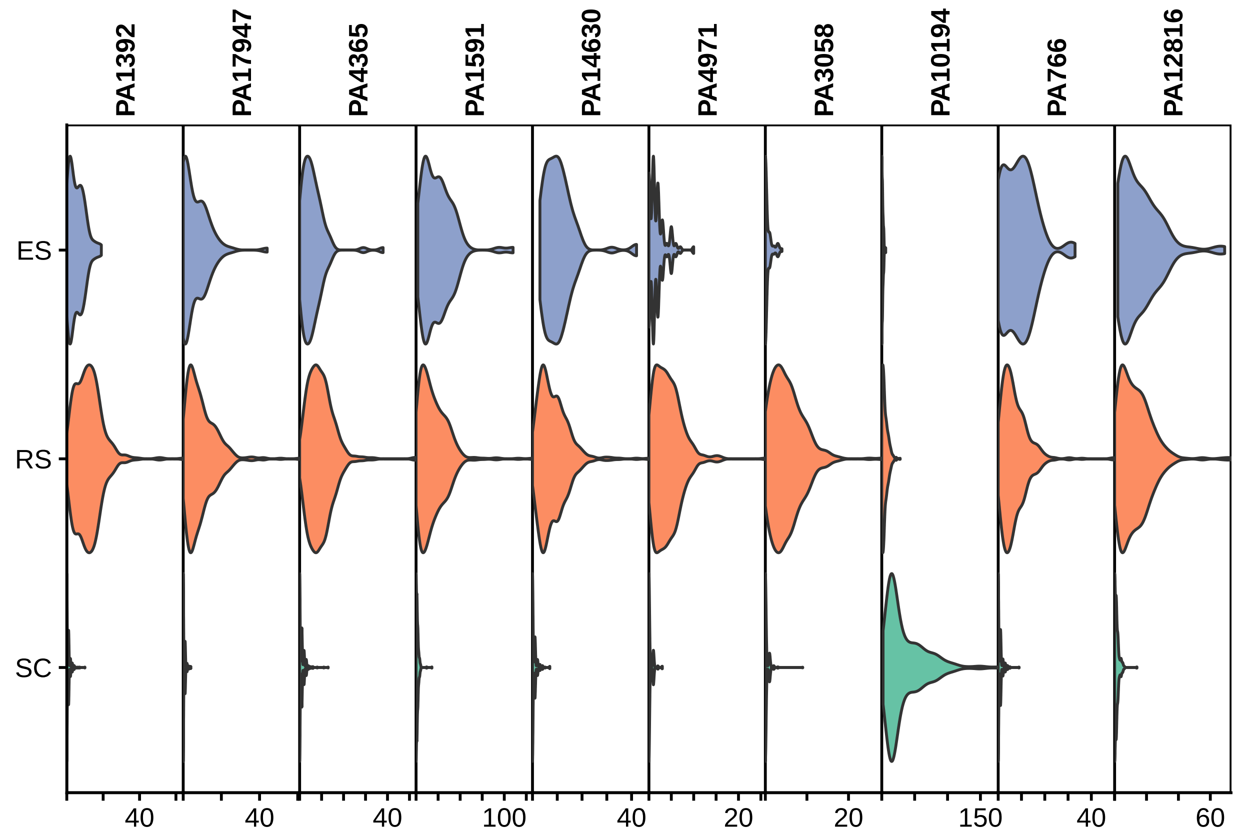

Get APA markers for each cell type

The above examples detect markers between every pair of cell types. It is also possible to compare one cell type with all other cells.

```
# Detect markers between ES and all other cells.
m=getAPAmarkers(scPACds,  group='celltype', cluster1='ES')
```

```
## PACds row = PA, PACds dataType = count
## Warning: it seems that PACds is pA count, will get DE pAs (each row is a pA).
## If you want APA markers, please use getAPAindexPACds() to transform your PACds to APA ratio.
```

```
table(m$cluster1, m$cluster2)
```

```
##
##      non-ES
##   ES    567
```

Similarly, we can plot top markers. Here we used violin plot to show the top 5 markers for demonstration. It can be seen that these markers are all with higher RUD scores in SC.

```
vizAPAMarkers(scPACds, group='celltype',
              markers=m$rowid[1:5],
              figType = 'violin')
```

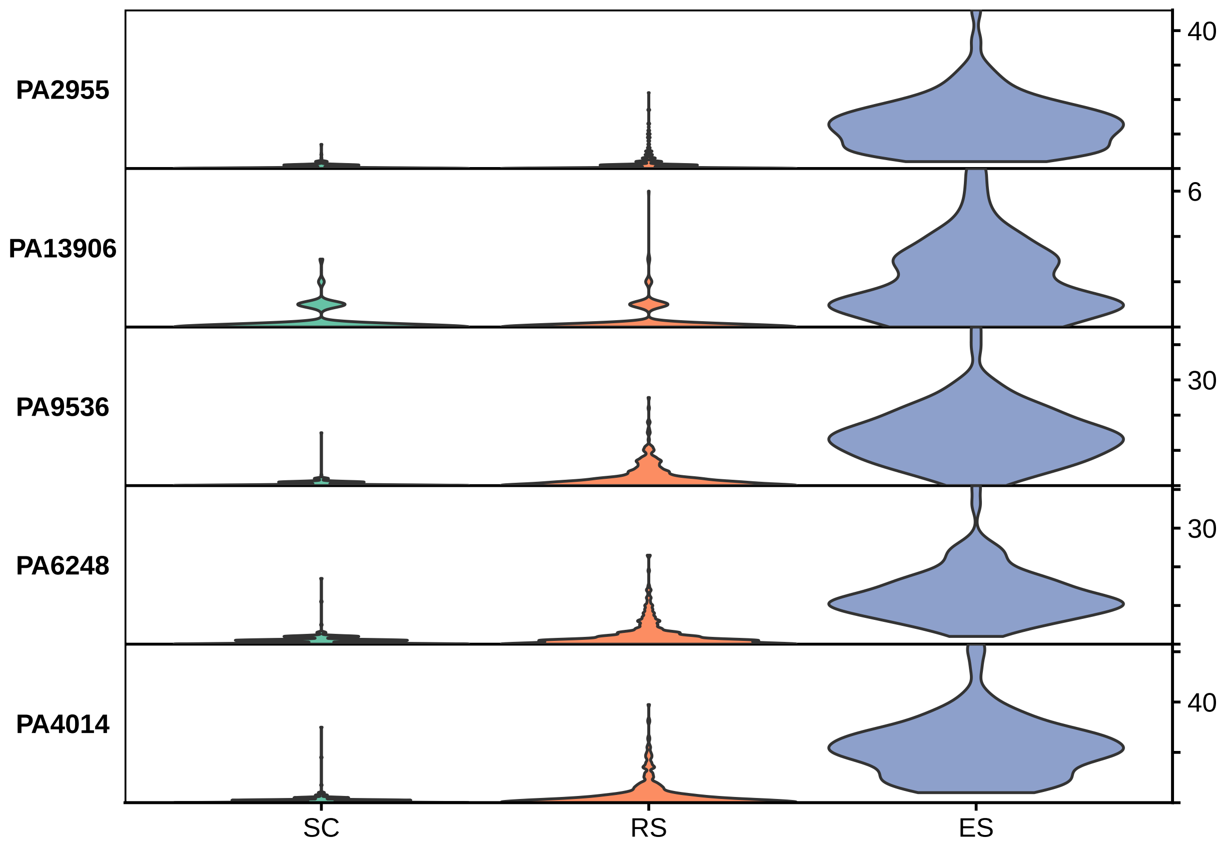

We can also compare exact two cell types.

```
m=getAPAmarkers(scPACds,  group='celltype', cluster1='ES', cluster2='RS')
```

```
## PACds row = PA, PACds dataType = count
## Warning: it seems that PACds is pA count, will get DE pAs (each row is a pA).
## If you want APA markers, please use getAPAindexPACds() to transform your PACds to APA ratio.
```

```
table(m$cluster1, m$cluster2)
```

```
##
##       RS
##   ES 461
```

We can also compare one cell type to each of all other cell types.

```
m=getAPAmarkers(scPACds,  group='celltype',
                cluster1='ES', cluster2=NULL,
                everyPair = TRUE)
```

```
## PACds row = PA, PACds dataType = count
## Warning: it seems that PACds is pA count, will get DE pAs (each row is a pA).
## If you want APA markers, please use getAPAindexPACds() to transform your PACds to APA ratio.
```

```r
table(m$cluster1, m$cluster2)
```

```
##
##        non-ES
##   ES      567
```

Similarly, we can plot top markers.

```r
vizAPAMarkers(scPACds, group='celltype',
              markers=m$rowid[1:5],
              figType = 'violin')
```

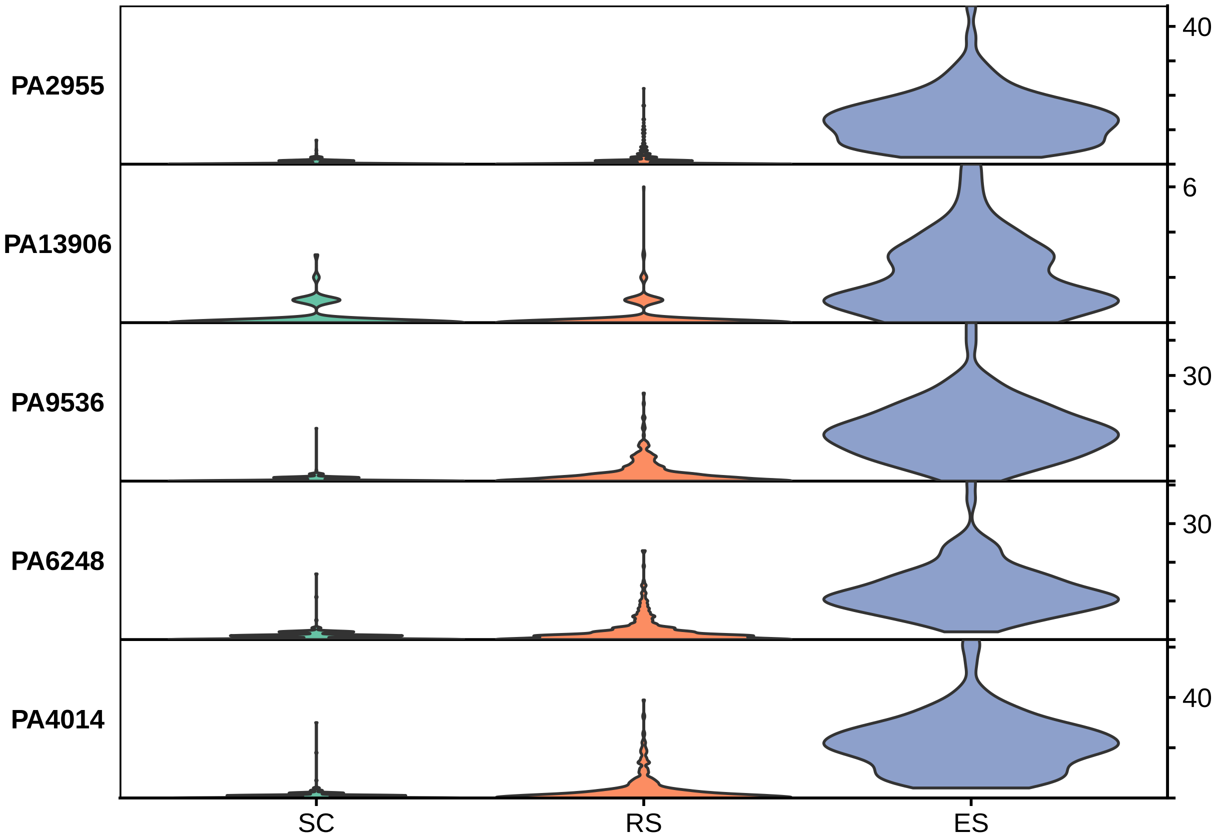

Session information

The session information records the versions of all the packages used in the generation of the present document.

```r
sessionInfo()
```

```
## R version 4.2.2 (2022-10-31 ucrt)
## Platform: x86_64-w64-mingw32/x64 (64-bit)
## Running under: Windows 10 x64 (build 22621)
```

```
##
## Matrix products: default
##
## locale:
## [1] LC_COLLATE=Chinese (Simplified)_China.utf8
## [2] LC_CTYPE=Chinese (Simplified)_China.utf8
## [3] LC_MONETARY=Chinese (Simplified)_China.utf8
## [4] LC_NUMERIC=C
## [5] LC_TIME=Chinese (Simplified)_China.utf8
##
## attached base packages:
## [1] stats4    stats     graphics  grDevices utils     datasets  methods
## [8] base
##
## other attached packages:
##  [1] Mus.musculus_1.3.1
##  [2] TxDb.Mmusculus.UCSC.mm10.knownGene_3.10.0
##  [3] org.Mm.eg.db_3.16.0
##  [4] GO.db_3.16.0
##  [5] OrganismDbi_1.40.0
##  [6] GenomicFeatures_1.50.2
##  [7] GenomicRanges_1.50.1
##  [8] GenomeInfoDb_1.34.9
##  [9] AnnotationDbi_1.60.0
## [10] IRanges_2.32.0
## [11] S4Vectors_0.36.0
## [12] Biobase_2.58.0
## [13] BiocGenerics_0.44.0
## [14] vizAPA_0.1.0
##
## loaded via a namespace (and not attached):
##   [1] rappdirs_0.3.3            rtracklayer_1.58.0
##   [3] scattermore_0.8           GGally_2.1.2
##   [5] SeuratObject_4.1.3        tidyr_1.2.1
##   [7] ggplot2_3.4.0             bit64_4.0.5
##   [9] knitr_1.41                irlba_2.3.5.1
##  [11] DelayedArray_0.24.0       data.table_1.14.6
##  [13] rpart_4.1.19              KEGGREST_1.38.0
##  [15] RCurl_1.98-1.9            AnnotationFilter_1.22.0
##  [17] doParallel_1.0.17         generics_0.1.3
##  [19] movAPA_0.2.0              cowplot_1.1.1
##  [21] RSQLite_2.2.18            RANN_2.6.1
##  [23] future_1.30.0             bit_4.0.5
##  [25] spatstat.data_3.0-0       xml2_1.3.3
##  [27] httpuv_1.6.6              SummarizedExperiment_1.28.0
##  [29] assertthat_0.2.1          xfun_0.35
##  [31] hms_1.1.2                 evaluate_0.18
##  [33] promises_1.2.0.1          fansi_1.0.3
##  [35] restfulr_0.0.15           progress_1.2.2
##  [37] dbplyr_2.2.1              igraph_1.3.5
##  [39] DBI_1.1.3                 htmlwidgets_1.5.4
##  [41] reshape_0.8.9             spatstat.geom_3.0-3
##  [43] purrr_1.0.2               ellipsis_0.3.2
##  [45] ggnewscale_0.4.8          dplyr_1.0.10
```

```
##  [47] ggpubr_0.5.0               backports_1.4.1
##  [49] biomaRt_2.54.0             deldir_1.0-6
##  [51] MatrixGenerics_1.10.0      vctrs_0.6.5
##  [53] Cairo_1.6-0                ensembldb_2.22.0
##  [55] ROCR_1.0-11                abind_1.4-5
##  [57] cachem_1.0.6               withr_2.5.0
##  [59] BSgenome_1.66.2            progressr_0.12.0
##  [61] checkmate_2.1.0            sctransform_0.3.5
##  [63] GenomicAlignments_1.34.0   prettyunits_1.1.1
##  [65] goftest_1.2-3              cluster_2.1.4
##  [67] lazyeval_0.2.2             crayon_1.5.2
##  [69] spatstat.explore_3.0-5     pkgconfig_2.0.3
##  [71] labeling_0.4.2             nlme_3.1-160
##  [73] ProtGenerics_1.30.0        nnet_7.3-18
##  [75] rlang_1.1.2                globals_0.16.2
##  [77] lifecycle_1.0.3            miniUI_0.1.1.1
##  [79] filelock_1.0.2             BiocFileCache_2.6.0
##  [81] dichromat_2.0-0.1          polyclip_1.10-4
##  [83] matrixStats_0.63.0         lmtest_0.9-40
##  [85] graph_1.76.0               Matrix_1.5-3
##  [87] carData_3.0-5              zoo_1.8-11
##  [89] base64enc_0.1-3            ggridges_0.5.4
##  [91] GlobalOptions_0.1.2        png_0.1-7
##  [93] viridisLite_0.4.1          rjson_0.2.21
##  [95] bitops_1.0-7               KernSmooth_2.23-20
##  [97] Biostrings_2.66.0          blob_1.2.3
##  [99] shape_1.4.6                stringr_1.4.1
## [101] spatstat.random_3.0-1      parallelly_1.33.0
## [103] jpeg_0.1-10                rstatix_0.7.1
## [105] ggsignif_0.6.4             scales_1.2.1
## [107] memoise_2.0.1              magrittr_2.0.3
## [109] plyr_1.8.8                 ica_1.0-3
## [111] zlibbioc_1.44.0            compiler_4.2.2
## [113] tinytex_0.43               BiocIO_1.8.0
## [115] RColorBrewer_1.1-3         clue_0.3-63
## [117] fitdistrplus_1.1-8         Rsamtools_2.14.0
## [119] cli_3.6.1                  XVector_0.38.0
## [121] listenv_0.9.0              patchwork_1.1.2
## [123] pbapply_1.6-0              htmlTable_2.4.1
## [125] Formula_1.2-4              MASS_7.3-58.1
## [127] tidyselect_1.2.0           stringi_1.7.8
## [129] highr_0.9                  yaml_2.3.6
## [131] latticeExtra_0.6-30        ggrepel_0.9.2
## [133] grid_4.2.2                 VariantAnnotation_1.44.0
## [135] tools_4.2.2                future.apply_1.10.0
## [137] parallel_4.2.2             circlize_0.4.15
## [139] rstudioapi_0.14            foreach_1.5.2
## [141] foreign_0.8-83             gridExtra_2.3
## [143] farver_2.1.1               Rtsne_0.16
## [145] digest_0.6.30              BiocManager_1.30.19
## [147] shiny_1.7.3                Rcpp_1.0.9
## [149] car_3.1-1                  broom_1.0.2
## [151] later_1.3.0                RcppAnnoy_0.0.20
## [153] httr_1.4.4                 ggbio_1.46.0
```

```
## [155] biovizBase_1.46.0          ComplexHeatmap_2.14.0
## [157] colorspace_2.0-3           XML_3.99-0.12
## [159] tensor_1.5                 reticulate_1.30
## [161] splines_4.2.2              uwot_0.1.14
## [163] RBGL_1.74.0                spatstat.utils_3.0-1
## [165] sp_1.5-1                   plotly_4.10.1
## [167] xtable_1.8-4               jsonlite_1.8.3
## [169] R6_2.5.1                   Hmisc_5.0-0
## [171] pillar_1.9.0               htmltools_0.5.3
## [173] mime_0.12                  glue_1.6.2
## [175] fastmap_1.1.0              BiocParallel_1.32.1
## [177] codetools_0.2-18           utf8_1.2.2
## [179] lattice_0.20-45            spatstat.sparse_3.0-0
## [181] tibble_3.2.1               curl_4.3.3
## [183] leiden_0.4.3               magick_2.7.3
## [185] interp_1.1-3               limma_3.54.0
## [187] survival_3.4-0             rmarkdown_2.18
## [189] munsell_0.5.0              GetoptLong_1.0.5
## [191] GenomeInfoDbData_1.2.9     iterators_1.0.14
## [193] reshape2_1.4.4             gtable_0.3.1
## [195] Seurat_4.3.0
```
